# Observed Hydroclimatic Trends and Their Implications Over Water‐Cycle Dynamics in Montérégie, Southern Quebec, Canada

**DOI:** 10.1002/gch2.202500564

**Published:** 2026-04-06

**Authors:** Jorge Mona, Christin Müller, Janie Masse‐Dufresne, Alexandra Mattei, Florent Barbecot

**Affiliations:** ^1^ Department of Earth and Atmospheric Sciences Université du Québec à Montréal ‐ UQAM Montréal Québec Canada; ^2^ GEOTOP ‐ Research Centre on the Dynamics of the Earth System Montréal Québec Canada; ^3^ Quebec Water Research Centre ‐ CentrEau, Pavillon Adrien‐Pouliot Université Laval Quebec Canada; ^4^ Hydrology Climate and Climate Change laboratory – École de technologie supérieure ‐ ÉTS Montréal Québec Canada; ^5^ Culletivita di Corsica Ajaccio France

**Keywords:** climate impacts, groundwater resources, hydroclimatic trends, piezometric levels, recharge processes, streamflow dynamics, sustainable management

## Abstract

A comprehensive understanding of groundwater recharge processes is essential for sustainable water resource management under climate change and increasing anthropogenic pressures. This study evaluates hydroclimatic trends (1980–2023) and their impacts on water‐cycle dynamics in Montérégie, a predominantly agricultural and forested region in southern Quebec, Canada, characterized by spring snowmelt and biannual baseflow discharge. Historical records of temperature, precipitation, streamflow, groundwater levels, and land cover are analyzed for 20 subwatersheds and 50 piezometers, with missing data addressed using Multiple Imputation by Chained Equations. Trends are assessed using Mann‐Kendall and Sen's Slope tests, while potential groundwater recharge is simulated with the GR4J hydrological model coupled with the CemaNeige snow module. Results indicate significant regional warming, particularly in summer and autumn, leading to longer growing seasons and more frequent winter melt events. Precipitation intensity has increased, contributing to slightly higher winter streamflow. Approximately 75% of annual GR4J percolation occurs during winter‐spring. Recharge increases in the Baie Missisquoi due to earlier snowmelt but declines elsewhere because of higher evapotranspiration and drier soils. Groundwater levels rise in central Richelieu but decline in central‐western Montérégie due to pumping and limited recharge capacity. These findings support adaptive groundwater management.

## Graphical abstract

In this study, we quantified the changes in different hydroclimatic parameters and evaluated their impacts on the groundwater resources across Montérégie. Using the GR4J+CemaNeige model, and multi‐decadal hydroclimatic records, the study shows the watershed's variability and identifies seasons and land cover scenarios where groundwater recharge variability is occurring, providing baselines for water resources planning under climate and land cover changes.

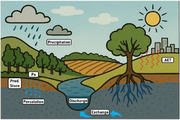

## 1 Introduction

Ecosystems and human societies are increasingly threatened by the impacts of climate change on water resources, as evidenced by global climate records from weather stations, satellites, and radar, altering all components of the global water cycle [1]. Rising temperatures and changes in precipitation patterns are influencing evapotranspiration processes and soil moisture dynamics [2, 3, 4, 5, 6, 7, 8]. Additionally, the shrinking of glaciers, ice, and snow cover is drastically affecting the hydrogeological cycle in many regions [9, 10, 11]. These climatic shifts, combined with anthropogenic activities, are driving changes in low and high‐streamflow patterns observed in watersheds worldwide [12, 13, 14].

Groundwater represents the largest global reservoir of freshwater; it supplies about 36%, 42%, and 27% of water for domestic, agricultural, and industrial uses, respectively [15, 16]. While groundwater in arid regions often shows long response times, buffering these regions against climate change, shallow aquifers in humid regions respond rapidly and cannot always sustain dependent ecosystems such as wetlands. These ecosystems are therefore less resilient to both climate variability and human pressures, including land cover change, extraction, and irrigation [17]. This reduced resilience amplifies impacts on groundwater recharge dynamics, affecting both the timing and magnitude of recharge events [18, 19]. Studies have documented increases as well as decreases in groundwater recharge in response to climate and land cover changes, with projections indicating continued variability [16, 20, 21]. Given groundwater's important role in water supply, such variability brings challenges not only for ecosystem sustainability but also for human water security.

High latitude regions (>30°) have experienced significant increases in precipitation, intensity, and ice/snow melt, leading to greater runoff, groundwater recharge, and river discharge [1]. These regions, which include watersheds fed by glaciers and snowmelt, are home to more than one‐sixth of the global population [22]. The seasonality and recharge mechanisms of these watersheds are particularly vulnerable to climate and land cover changes [11, 18, 20, 23, 24, 25].

A prominent example in North America is the Great Lakes‐St. Lawrence River system, which contains approximately 20% of the world's freshwater and supports one‐third of Canada's population and 10% of the U.S. population [26]. This system drains northeast, converging with the Outaouais River before reaching the Gulf of St. Lawrence in the province of Quebec, in eastern Canada. About 10% of the surface of Quebec is covered by water, of which 40% is in the St Laurent watershed [27]. The Montérégie region, located between Montréal and the U.S. border, is a key agricultural area and a hub for Quebec's population that exemplifies temperate, humid, high‐latitude environments with transboundary watersheds, where water resources, vegetation, and agricultural production are increasingly affected by climate change and anthropogenic activities [18, 28, 29, 30, 31]. Projected changes include altered discharge patterns, interannual variability in groundwater recharge, and shifts in land cover [23, 24, 32].

Groundwater recharge results from complex interactions between precipitation, evapotranspiration, land cover, and streamflow dynamics [33]. These processes are highly sensitive to seasonal and interannual variability, especially in snow‐influenced aquifers [23, 32, 34]. For this reason, the detection of long‐term trends in hydroclimatic parameters is crucial to quantify changes in the magnitude, timing, and direction of water fluxes, and evaluate their impact on groundwater recharge and baseflow contributions to rivers, lakes, and ecosystems [13, 32, 35, 36]. Non‐parametric statistical tests such as the Mann–Kendall test and Sen's‐Slope estimator are commonly used to identify monotonic trends in non‐normally distributed time‐series [37, 38, 39]. Supported by p‐value assessments, these methods provide statistical solid basis to detect significant changes over time and distinguish between natural variability and persistent changes. However, for a reliable trend analysis, it is important to have a continuous time‐series, which is often challenging due to the usual technical problems of measurement. By recognizing the different trends that exist in a study area, it is possible to identify vulnerable regions where recharge may be decreasing or where it has great variability, and to provide information for better territorial planning and better management of water resources.

In this study, we compiled and analyzed long‐term hydroclimatic datasets from multiple watersheds across Montérégie, including air temperature, precipitation, streamflow, land cover, and piezometric levels. Missing values from different records were addressed using a sequential multiple imputation technique to ensure a reliable trend analysis. Hydroclimatic trends were assessed using non‐parametric statistical tests, while land cover was evaluated using satellite data to explore its influence on recharge dynamics. The percolation in the non‐saturated zone was simulated with GR4J+Cemaneige to estimate potential recharge for each watershed. Furthermore, hydroclimatic anomalies were cross‐correlated with the ONI index to explore teleconnection between global ENSO signals and regional water dynamics.

This study offers a novel, regionally integrated regional assessment linking long‑term climate, streamflow, and groundwater dynamics over several decades in a cold‑temperate agricultural region. By connecting observed streamflow and groundwater‑level trends with climate data and hydrological model outputs, it is possible to show how groundwater recharge is changing over time and space. This approach clarifies how hydroclimatic changes impact through surface and subsurface systems, supporting climate‑resilient groundwater management in Montérégie.

## 2 Materials And Methods

### 2.1 Study Area

**FIGURE 1:**
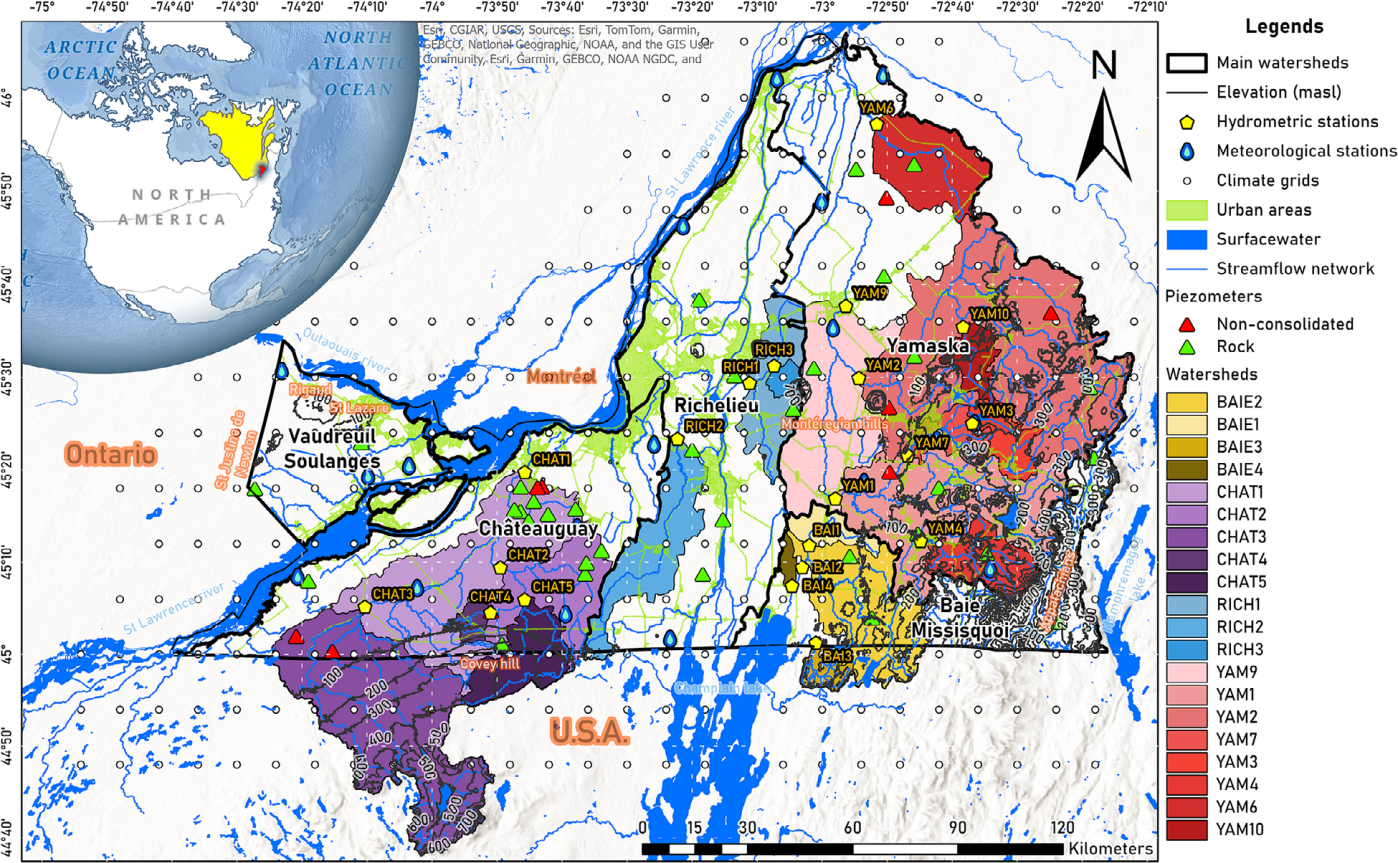
Map of Montérégie, in southwestern Québec, illustrating the main watersheds (black straight boundaries), their sub‐watersheds (color‐shaded), and elevation contours (every 100 m a.s.l.). The location of the climatic grids (white points), meteorological stations (blue drops), hydrometric stations (yellow pentagons), and piezometers (triangles) with records used in this study are shown. Urban areas and main highways are shown in green, while surface water and the river network are shown in blue.

The Montérégie aquifers region (∼12 000 km^2^), located in southern Quebec, Canada, is recognized as the province's pantry, contributing 32% of its agricultural income [40]. Situated between Montréal and the U.S. border (Figure 1), it is the second most populated region in Quebec, with ∼1.6 million inhabitants across 186 municipalities [41]. The region has a cold, humid continental climate (Dfb, Köppen‐Geiger classification), characterized by cold winters, warm summers, and no dry season [42].

Montérégie is divided into five main watersheds (Figure 1): Vaudreuil‐Soulanges (VS), Châteauguay (CHAT), Richelieu (RICH), Baie Missisquoi (BAIE), and Yamaska (YAM) [43, 44, 45, 46, 47]. YAM is the only watershed located entirely in Quebec; the rest of the watersheds have more than half of their surface outside the province [30]. Natural groundwater recharge and discharge are primarily driven by snowmelt and rainfall, while discharge is modulated by evapotranspiration and baseflow dynamics. The most pronounced seasonal variations in recharge and discharge typically occur in late‐winter/early‐spring and in autumn (Figure 10). Recharge generally occurs in upland areas, whereas discharge is directed toward the St. Lawrence River and its tributaries, especially in areas underlain by sedimentary rocks or coarse‐grained unconsolidated sediments [48, 49, 50]. Also, the eskers (permeable moraine deposits remaining from the last glacial epoch that overlies the bedrock) are considered preferential areas for recharge.

The regional aquifer transitions from unconfined to confined conditions as it flows from recharge zones toward lowland areas covered by fine sediments [43, 48, 49, 50]. In these lowlands, discontinuous granular lenses and shallow deposits create local aquifers with potential use [43, 49]. Regional numerical studies in southern Québec suggest that groundwater circulation is often dominated by local flow systems concentrated in the shallow fractured bedrock, while intermediate pathways and topographic features can locally moderate groundwater‐surface water exchanges and associated baseflow [51, 52]. This configuration aligns with observed water quality patterns, where the regional aquifer evolves from fresh conditions in recharge zones to brackish in lowlands, reflecting the influence of underlying marine or lacustrine sediments [48]. Baseflow and surface water quality are highly associated with groundwater and its resurgence zones [53]. Surface runoff and subsurface lateral flow from shallow aquifers (more vulnerable to anthropogenic activities with high pollution potential, e.g., urban development, agriculture, industry, etc.) can significantly alter water quality.

#### 2.1.1 Geological Bedrock

The bedrock geology of Montérégie is divided into two provinces: the St. Lawrence platform and the Appalachians [54]. The St. Lawrence platform, dating to the Middle Cambrian and Upper Ordovician periods, consists of quartzitic sandstones, conglomerates, dolomites, limestones, calcareous shales, and molassic red beds deposited in a marine‐lake environment. The Appalachian region, located in the southeastern corner, includes the Humber and Dunnage zones, which are composed of basalts, arkoses, sedimentary rocks, shales, phyllites, and mafic volcanic rocks. Cretaceous igneous intrusions, known as the Montérégiennes Hills, are also present in the eastern part of the region (Figure 1). Groundwater flow in the bedrock aquifer is controlled by a heterogeneous fracture network, with transmissivities that are often underestimated and decrease substantially with depth, such that shallow fractured zones tend to transport most of the groundwater flow [55].

#### 2.1.2 Quaternary Sediments

**FIGURE 2:**
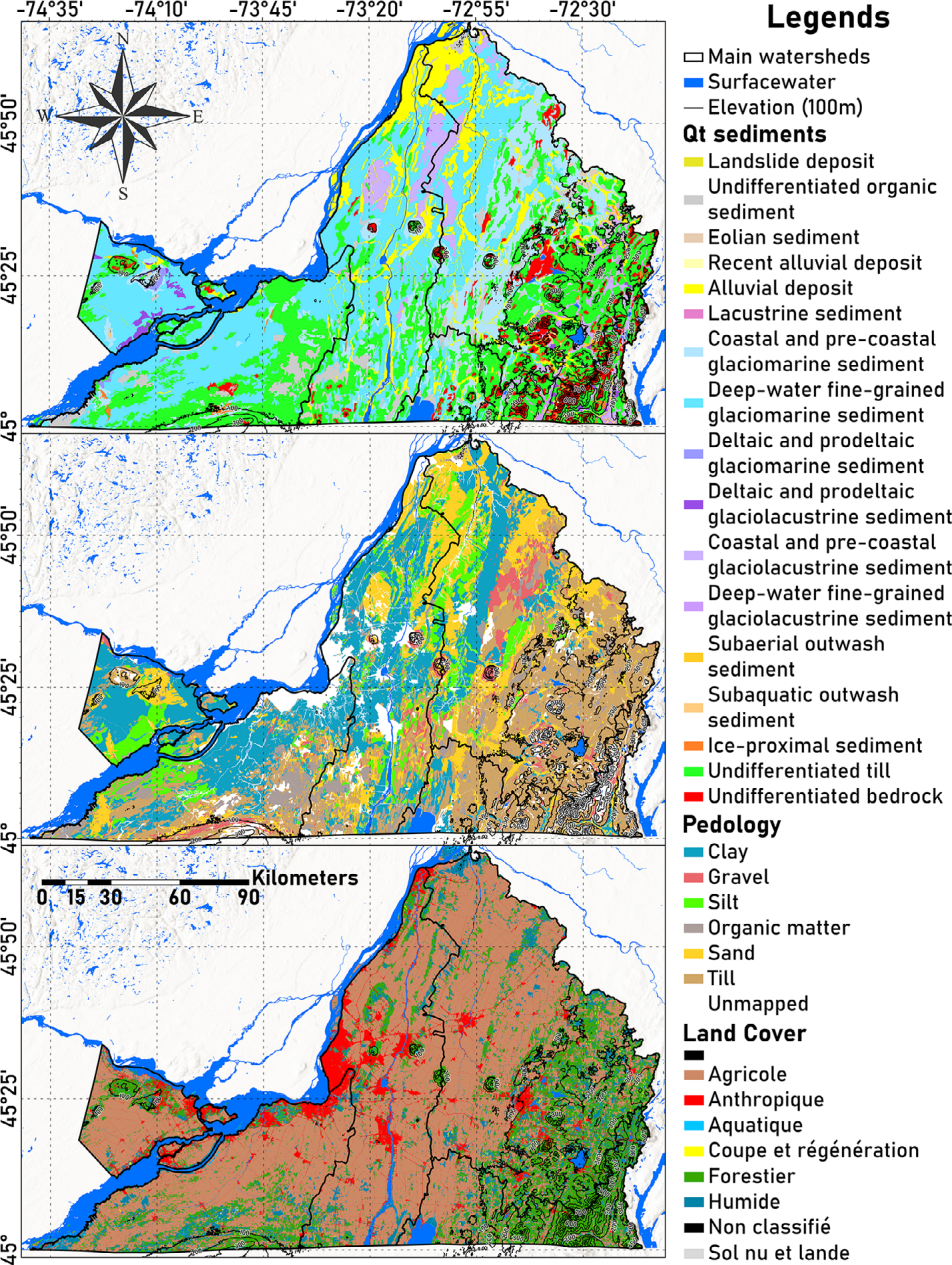
Maps of Montérégie showing its Quaternary sediments (top), pedology (middle), and land cover (bottom). Quaternary data were sourced from the Service de Systèm d'Informations Géominières (SIGÉOM) of Quebec's Government, pedological data were provided by the Institut de Recherche et de Développement en Agroenvironnement (IRDA), and land cover data were obtained from the Quebec's Ministry of Environment [56, 57, 58].

Overlying the bedrock is a discontinuous layer of Quaternary sediments (Figure 2, top), primarily consisting of tills and fluvio‐glacial deposits from the Wisconsin glaciation (∼23–10 ka) [59]. These sediments were shaped by glacial advances and retreats, followed by marine and fluvial sedimentation. The Champlain Sea, which occupied the region until approximately 10 ka, left behind glaciomarine sediments (∼41%), glacial sediments (∼28%), alluvial sediments (∼14%), glaciolacustrine sediments (∼7%), and glaciofluvial sediments (∼1%). These, along with bedrock (∼5%), organic sediments (∼4%), eolian sediments, and landslide deposits (<0.2%), compose the Quaternary sediments [55, 56, 60, 61]. Based on a regional 3D geological model developed for the south of Quebec, the Quaternary sediments can be grouped into three main hydrostratigraphic units: sand, clay, and till [62]. Their respective thickness varies considerably across the region. Sandy sediments are typically shallow (1–10 m), but deltaic deposits can reach 20–40 m. Clay‐rich sediments (e.g., Champlain sea clays) are more widespread and on average twice as thick, with localized thicknesses up to 100 m. The basal till and sub‐till units are the most extensive and may locally exceed 30 m, with a maximum recorded thickness of ∼150 m in buried valleys.

#### 2.1.3 Pedology

Soil types in Montérégie (Figure 2, middle), derived from weathering of Quaternary sediments and bedrock, include till (∼28%), clay (∼24%), sand (∼16%), silt (∼7%), organic matter (∼5%), and gravel (∼4%). The remaining area consists of outcrops, urban zones, and unclassified soils (∼16%) [57, 63].

#### 2.1.4 Recent Land Cover

The recent land cover in Montérégie (Figure 2, bottom) is dominated by agriculture (∼54%), forest (∼21%), wetland (∼9%), anthropogenic (∼9%), surface water (∼6%), logging and regeneration (∼0.5%), and bare soil (<0.1%). The main agricultural crops are corn (∼22%) and soybeans (∼15%), as well as perennial crops and pastures (∼11%), while maple trees (∼13%) are the main forest occupation [58].

#### 2.1.5 Topography

The topography of Montérégie is mostly flat near the St. Lawrence River (below 100 m a.s.l.), with elevations reaching approximately 1000 m a.s.l. in the southeastern Appalachians and around 400 m a.s.l. near Covey Hill in the southwest (Figure 1). In the west, we can find the hills of Rigaud (∼220 m a.s.l.), St Lazare (∼120 m a.s.l.), and St Justine de Newton (∼100 m a.s.l.). The Montérégiennes Hills, with elevations ranging from around 220 to 550 m a.s.l., are prominent features in the central‐east region [64].

#### 2.1.6 Water Supply

Surface water is mainly sustained by groundwater discharges, and supplies 72.5% of Montérégie's population, while groundwater extraction provides the remaining 27.5%, coming from municipal networks (9.5% of total consumption) and private wells (18%) [27, 53, 65]. There are 34 surface water filtration plants in Montérégie, responsible for making it potable for the population's consumption. Agriculture, however, remains the dominant water user in the region, representing 53% of total pumping (∼68 million m^3^ year^−1^). Irrigation accounts for roughly 85% of agricultural demand, especially for vegetable crops, orchards, and vineyards, while livestock watering and farm operations contribute the rest. Most of this consumption relies on groundwater, with surface water serving as a seasonal complement [66].

### 2.2 Data Compilation

#### 2.2.1 Climate Data (Temperature and Precipitation)

Daily temperature (minimum and maximum) and precipitation data (1961–2017) were obtained from 302 climate grids from the Quebec Climate Monitoring Network [67]. Recent data (2018‐present) were sourced from 19 meteorological stations within Montérégie (Figure 1). Missing data were imputed using a sequential Multiple Imputation by Chained Equations (MICE) algorithm (see Section 2.3).

Quebec's Climate Monitoring Network was created from weather station data (automatic and with observers) of different institutions, collected during four different periods (1961–1990, 1971–2000, 1981–2010, and 1991–2017), and passed through different validation steps to control the data quality. The spatial variability of each grid was estimated with the Kriging interpolation method, with variogram parameters daily estimated with a spherical model for total precipitation and temperature, considering the ten closest weather stations and a maximum separation distance of 200 km between each point [67]. The spatial domain of the network extends over the entire Quebec, as well as parts of the Outauais and RICH watersheds along its southern and southwestern borders (55° to 81.5° W, 43° to 63° N), with a resolution of 0.1° (∼10 km) between each data point.

#### 2.2.2 Streamflow and Groundwater Data

Daily records of streamflow from 20 hydrometric stations and groundwater levels from 50 piezometers (Figure 1) were used [68]. Data before 1980 (streamflow) and 2000 (groundwater) were excluded due to poor spatio‐temporal resolution. Missing streamflow data were imputed using sequential MICE (see Section 2.3).

The earliest streamflow record dates back to 1928 (station YAM1), and there are currently 26 stations recording daily streamflows. The records from six stations were discarded: five from sub‐watersheds with records shorter than ten years (four in YAM and one in RICH), and the main RICH watershed, whose surface area is mostly in the USA (∼75%), and it has the highest discharge recorded within the Montérégie region. Due to the poor spatial and temporal resolution of discharge records before 1980, only records after this date were considered in this study. The remaining 20 stations are located in the outflows of sub‐watersheds of varying sizes (26–3334 km^2^), situated at different elevations (upstream, midstream, and downstream), and covering diverse land cover types. It should be noted that these stations do not represent the entire surface of the main watersheds shown in Figure 1. Instead, each hydrometric station reflects only its specific contributing sub‐watershed, delineated by the color‐shaded polygons in Figure 1. Specifically, five stations are in CHAT watershed, three in RICH, four in BAIE, and eight in YAM. Upslope sub‐watersheds have more recent discharge records (beginning in the 2000's), while the mid‐downslope sub‐watersheds have the oldest records (beginning in the late 1960's; Table 1).

**TABLE 1:** Main physiographic and hydroclimatic features of the gauged watersheds. Elevation (Elev) is the weighted spatial average obtained from the DEM [69]. Mean temperature (*T*_avg_) is the daily average. Potential evapotranspiration (PET) and precipitation (Prcp) are long‐term means of yearly totals [70]. Precipitation intensity (Intns) corresponds to the Simple Daily Intensity Index (SDII), expressed as the long‐term mean of yearly values derived from monthly SDII (see Section 2.4). Specific discharge (Q) is the daily average normalized by watershed area. Watersheds are ordered from top to bottom by elevation (Figure 1). Color gradients highlight relative magnitudes within each column (darker = higher values; lighter = lower values).

Watershed	1st discharge record	Area (Km^2^)	Elev (m a.s.l.)	*T*_avg_ (°C day^−1^)	PET (mm year^−1^)	Prcp (mm year^−1^)	Intns (mm day^−1^)	Q (mm day^−1^)
BAIE2	Nov/2001	584	111	6.7	631	1151	6.0	1.5
BAIE3	Nov/2001	73	79	7.0	643	1126	6.2	1.3
BAIE1	Jul/1999	94	68	6.8	636	1139	6.2	1.4
BAIE4	Nov/2001	26	44	6.8	640	1096	6.3	1.5
CHAT5	Jul/2005	246	113	6.4	622	951	5.7	1.3
CHAT4	Jul/2005	47	102	6.9	642	979	5.9	1.2
CHAT2	Oct/1973	642	79	6.7	633	950	5.6	1.2
CHAT1	Apr/1970	2492	67	6.6	631	951	5.7	1.3
CHAT3	Sep/2004	1051	65	6.0	614	997	5.7	1.5
RICH3	Jul/2006	35	56	6.7	639	1064	6.4	1.8
RICH2	Aug/1979	367	53	6.8	639	973	5.6	1.1
RICH1	Oct/1973	308	49	6.7	639	1065	6.2	1.4
YAM4	Aug/1968	214	264	6.2	614	1270	6.7	1.8
YAM3	Apr/1968	131	225	6.0	608	1174	6.6	1.9
YAM1	Aug/1965	1231	194	6.2	617	1162	6.2	1.7
YAM7	Nov/1983	235	190	6.2	618	1175	6.5	1.9
YAM2	Aug/1965	1505	147	6.2	617	1125	6.1	1.5
YAM9	Oct/1994	3334	147	6.3	622	1130	6.0	1.6
YAM10	Jun/2000	102	121	5.8	603	1094	6.4	1.6
YAM6	Apr/1969	323	55	6.1	621	1023	6.2	1.4

The earliest recorded groundwater level date back to 1974, and until the 1990s, only about 10 piezometers recorded groundwater levels, typically at monthly or lower resolution. Starting in the 2000s, around 30 piezometers resumed monitoring, with an additional 30 being incorporated after 2010. Due to their limited spatial and temporal resolution, records before 2000 were discarded, as were piezometers with less than 10 years of data or more than 40% missing values or FMI entries (see Section 3.1). We used available records from 50 piezometers: two located in VS, 20 in CHAT, five in RICH, seven in BAIE, and 16 in YAM (Figure 1). According to MELCCFP, these piezometers measure groundwater levels from the rocky regional aquifer or from non‐consolidated sediments above it [71].

#### 2.2.3 Historical Land Cover Data

Historical land cover data (1985–2020) were obtained from the GLC_FCS30D dataset, at a resolution of 30 m per pixel [72]. For clarity, land cover was reclassified into five categories: croplands, forests, open vegetation (including shrublands, grasslands, and low‐density vegetation), urban and bare areas, and aquatic areas (including wetlands and water bodies) (Table 2).

**TABLE 2:** Percentage of main land cover categories in each watershed in 2020 [72]. Categories were reclassified from the GLC_FCS30D dataset into croplands, forests, open vegetation, urban and bare areas, and aquatic areas. Watersheds are ordered from top to bottom by elevation (Figure 1). Color gradients highlight relative magnitudes within each column (darker blue = higher values; lighter blue = lower values).

Watershed	Croplands	Forests	Open vegetation	Urban and bare areas	Aquatic areas
BAIE2	39.4	56.6	1.8	1	1.2
BAIE3	49	48.1	2.5	0.3	0.14
BAIE1	56.7	40.6	1.6	1	0.04
BAIE4	89	10	0.1	0.9	0
CHAT5	15.4	81.4	2.7	0.3	0.17
CHAT4	25.2	68.5	4.4	1.7	0.28
CHAT2	27	68.8	3.3	0.8	0.17
CHAT1	35.5	60.8	2.1	0.9	0.78
CHAT3	22.1	74.5	1.8	0.2	1.34
RICH3	79.3	17.8	2.8		0.02
RICH2	64.7	30.1	1.8	3.3	0.08
RICH1	76.5	17	0.5	5.8	0.16
YAM4	8.8	88.1	1.5	1	0.61
YAM3	17.1	75.1	1.5	1.8	4.38
YAM1	21.7	71.7	1.5	3	2.09
YAM7	19.7	68	1.6	7.4	3.31
YAM2	40.3	56.8	0.9	1.7	0.32
YAM9	39.8	55.2	1.1	2.9	1
YAM10	29.3	69.7	0.6	0.3	0.04
YAM6	68.7	29.2	0.4	1.2	0.04

This dataset was developed using a combination of continuous change detection, locally adaptive updating models, and a spatiotemporal optimization algorithm applied to dense time‐series data from Landsat imagery. It provides a global record of land cover during the period between 1985 and 2022, divided into 35 subcategories. The land cover data were extracted for the 20 watersheds over five‐year period between 1985 and 2020.

### 2.3 Missing Data Imputation (MICE)

Missing data is a common issue in long‐term time‐series datasets due to technical problems that generate gaps in the monitoring. Traditional approaches, such as the substitution of missing values with averages or zeros, can disrupt natural patterns, reduce the power of statistical analyses, and lead to biased estimates of trends, correlations, or predictions. These issues are particularly problematic when time‐series data are used for trend analyses and hydrological modeling, as missing values can degrade the quality of model calibration and simulation predictions. To address this issue, advanced statistical tools have been developed to estimate missing values using historical records from the same station or from nearby stations monitoring related parameters, producing imputed values with lower associated uncertainties.

For meteorological, hydrometric, and piezometric stations with missing data, sequential Multiple Imputations by Chained Equations (MICE) were performed using the “mice” package in R to estimate missing values [73]. Predictive mean matching (PMM) was applied to each time‐series, with five imputations and 150 iterations to ensure robust estimates. The sequential imputation process began with the station having the oldest records and proceeded one‐by‐one to the most recent station. For each station, the imputation used climatic information as predictor variables (minimum and maximum temperature, and precipitation), and the results were incorporated into the predictor matrix for subsequent stations. This iterative sequential approach ensured that the imputation of the most recent station relied on both, its climatic data and the imputed values from preceding stations.

For hydrometric and piezometric stations, imputations were divided into two periods: before and after 2017. Before 2017, climate grid values were used as predictors, while post‐2017 imputations relied on data from the closest meteorological stations. To quantify the uncertainty associated with each time‐series imputations, a pooling (combination) of the univariate estimates from the five imputations was applied according to Rubin's rules [74]. This pooling was obtained from the coefficients of a linear mixed model that predicts the imputed values from the predictor variables (minimum and maximum temperature, precipitation, and other meteorological, hydrometric or piezometric stations). For each time‐series, the third imputation was selected to replace the missing values.

The application of MICE in filling missing data in different scientific fields has shown that performance decreases as the percentage of missing data increases, which has led to the establishment of different thresholds above which the use of MICE is discouraged [75, 76, 77]. Some studies suggest a low tolerance of missing values between 5% and 10%, while others report good performance when there are less than 60%–70% missing values [75, 77, 78, 79]. However, it has also been shown that, regardless of the proportion of missing data, the use of MICE is always beneficial in terms of bias reduction and efficiency improvement [80]. For this reason, the Fraction of Missing Information (FMI), derived from Rubin's rules, is considered a better guide‐parameter to determine the efficiency obtained from the set of five imputations [74, 80]. The FMI can be interpreted as the fraction of the total variance (both, between and within imputations) obtained from the estimation of the intercept of the mixed linear regression that exists between the five performed imputations, thus allowing to quantify the loss of information due to missingness (e.g. missing at random), while considering the information provided by the predictor variables during imputation. Thus, there may be imputation models with different proportions of missing data that produce similar FMI values with approximately the same empirical standard errors [80].

### 2.4 Statistical Data Treatment

The R programming language was used to process the hydroclimatic database and to statistically analyze the GR4J+CemaNeige model outputs (described in detail in Section 2.5) [81, 85, 86]. Climate grids located within the boundaries of the sub‐watersheds containing hydrometric gauges were spatially averaged to generate time‐series for each watershed (1961–2017). For CHAT4, the only sub‐watershed without climate grids inside its boundaries, data from the nearest grid was used. The specific climate time‐series of each watershed were completed up to 2023 with data from the nearest available meteorological station.

Daily temperature values were used to compute potential evapotranspiration (PET) by applying the Oudin (2004) model [70]. For comparison between watersheds, daily specific discharge (Q) was calculated by normalizing streamflow with watershed area. For precipitation, PET, and Q, accumulated monthly values were computed, while monthly average values were computed for temperature and groundwater levels. Monthly precipitation intensity was represented using the Simple Daily Intensity Index (SDII), defined as the total monthly precipitation divided by the number of wet days in that month [82]. In this study, wet days were considered those with precipitation ≥ 0.2 mm, and the resulting values are expressed in mm day^−1^. For simplicity, monthly values were used to define seasonal values: winter (Dec–Feb), spring (Mar‐May), summer (Jun‐Aug), and autumn (Sep‐Nov).

Hydroclimatic data and GR4J simulation outputs were also processed in R to evaluate the annual timing of key hydroclimatic events across the watersheds. These included the start and end of the non‐freezing and growing seasons, the day of maximum annual precipitation, the timing of annual maximum and minimum values of discharge, as well as the peaks of groundwater levels, and GR4J‐derived effective precipitation (Ps) and percolation. Due to the annual bimodal hydrological pattern observed in these last variables (Q, groundwater levels, Ps, and percolation), i.e. they have two peaks per year, their timings were determined separately for the winter‐spring and summer‐autumn periods.

#### 2.4.1 Historical Anomalies and Trends

To identify long‐term changes and trends in the data, anomalies were calculated to represent deviations from typical seasonal patterns. These anomalies eliminate recurring seasonal cycles, enabling a more detailed analysis of changes over an extended period. Monthly and seasonal anomalies were computed by subtracting the climatological mean (e.g., the average value of each month or season over a reference period) from the observed value. For climate, streamflow data, and GR4J simulation results, the considered climatological period was from 1980 to 2023, corresponding to the best spatio‐temporal resolution of hydrometric stations. For groundwater levels, only records after 2000 were considered; their climatological periods extended from the beginning of their records to 2023. To evaluate historical trends in the anomalies, the Mann–Kendall tau (MK(τ)) and *p*‐values, as well as Sen's‐Slope (SS) tests were applied to the time series of each month and season [37, 38, 39]. Throughout this study, trends derived from monthly anomalies are referred to as overall trends, and those based on seasonal anomalies as seasonal trends. These trend tests were also performed on the annual timing results (e.g. start/end of non‐freezing and growing seasons, and annual hydrological peaks/minimums) to assess their evolution over time. The Mann–Kendall test is widely used to detect monotonic trends in non‑normally distributed data, but it has limitations. Its assumption of serial independence is not always satisfied in hydroclimatic time series, where autocorrelation can skew trend significance, and it cannot capture non‑monotonic or stepwise changes. Even so, it remains a standard non‑parametric tool in hydroclimate analyses. To better interpret trend magnitude, it was complemented with Sen's Slope.

#### 2.4.2 Extreme Precipitation, Streamflow, and Recharge Events

To assess historical trends in the annual distribution of precipitation and its intensity, we computed, for each year, the 70th (P70) and 98th (P98) percentiles of all daily values (including 0 mm days). These are the thresholds below which 98% and 70% of all recorded daily precipitation values fall during certain year. In our records, non‐zero daily values arise around P70, making it a practical threshold for low‐intensity precipitation and consistent with evidence of a distributional breakpoint near this percentile [83]. We also identified the day of the year with the highest amount of precipitation. To account for the annual bimodal pattern of streamflow discharge, the values were divided into winter‐spring and summer‐autumn periods. We then calculated the annual values for high‐flow (Q95) and low‐flow (Q5) discharges in each period. Q95 and Q5 represent the 95th and 5th percentiles, respectively, that is, the daily discharge that is less than or equal to 95% and 5% of all recorded daily discharge values. These indices (Q95 and Q5) were used to evaluate long‐term trends in streamflow magnitudes. Additionally, we identified the dates on which the highest and lowest discharges occurred in each of these two periods (Figure 10), and these were used to analyze trends in the timing of extreme streamflow events throughout the year. In a similar way, daily groundwater levels from piezometers were analyzed to determine the timing of peak water table levels during each period (Figure 10). These dates were then used to evaluate long‐term changes in the occurrence of groundwater level events related to recharge.

#### 2.4.3 Non‐Freezing Season

When temperatures drop below the freezing point (<0°C), frozen pore water reduces soil porosity, causing precipitation to accumulate on the surface as snow or ice, which impedes runoff and infiltration. Consequently, infiltration and groundwater recharge primarily occur during periods with temperatures above 0°C. To examine changes in the length of the non‐freezing season across different watersheds, the last spring frost was defined as the first day of the year following seven consecutive days with an average temperature above 0°C. Similarly, the first autumn frost was defined as the first day of autumn after seven consecutive days with an average temperature below 0°C. The length of the non‐freezing season of each year was determined as the number of days between the last spring frost and the first autumn frost.

#### 2.4.4 Growing Season

Evaluating the length of the growing season is important for planning and managing agricultural production. Agriculture demands substantial water resources, which has an impact on the region's water balance and quality. The growing season is defined as the annual period in which the climatic conditions are favorable for crop growth.

According to Belanger et al. (2006) [84], in Montérégie, the beginning of the growing season (BGS) after winter is defined as the fifth day in a sequence of five consecutive days during which the weighted moving average of daily mean temperatures (WMAT5) exceeds 5.5°C:

$$\mathrm{BGS}=\min\;\left(j|\mathrm{WMAT}5_{j}>5.5^{\circ }C\right)$$

Similarly, the end of the growing season (EGS) is defined as the day when the WMAT5 permanently falls below 5.5°C:

$$\mathrm{EGS}=\max\;\left(j\;|\;\mathrm{WMAT}5_{j}\;>\;5.5^{\circ }C\right)+1$$

where the WMAT5 is calculated as:

$$\begin{array}{ccc} & & \mathrm{WMAT}5_{j}=\\ & & \left[T_{\mathrm{avg}\left(j-2\right)}+4T_{\mathrm{avg}\left(j-1\right)}+6T_{\mathrm{avg}\left(j\right)}+4T_{\mathrm{avg}\left(j+1\right)}+T_{\mathrm{avg}\left(j+2\right)}\right]/16 \end{array}$$

j=1,…,365

Thus, the length of the growing season (LGS) is defined as the number of days between the beginning and the end of the growing season:

LGS=EGS−BGS

### 2.5 Hydrological Modeling (CemaNeige+GR4J)

We used the airGR library of R to implement the GR4J (*Modèle du Génie Rural à 4 paramètres Journalier*) hydrological model, coupled with the CemaNeige snow accounting routine and historical streamflow data, to simulate and quantify precipitation‐percolation processes and calibrate the watershed‐specific parameters, to understand their spatio‐temporal behavior in Montérégie [85, 86, 87, 88, 89]. The simulated values of effective precipitation (Ps), which reach the soil moisture production store, and the percolation that leaks from it, were statistically treated for each subwatershed in a similar way as the hydroclimatic variables. Anomalies were computed, and trend tests were applied to their monthly series, as well as to seasonal series aggregated by hydrological periods (winter–spring and summer–autumn). In addition, the annual timing of Ps and percolation peaks was determined for each period to evaluate long‐term changes in their occurrence, following a similar approach as for streamflow and groundwater levels.

The GR4J model is a daily lumped, four‐parameter, hydrological model used to simulate watershed‐scale streamflow using precipitation and PET records as inputs. The four parameters that need to be calibrated are: (1) Production Store capacity (mm), representing the maximum water content that the soil moisture store can hold before excess becomes runoff. It is related to infiltration and recharge potential, making it important to assess how land cover and soils constrain groundwater recharge across watersheds. (2) Groundwater Exchange coefficient (mm day^−1^), representing the net exchange flux between the catchment and groundwater. Positive values indicate groundwater discharge to streams, whereas negative values indicate recharge to the aquifer. It allows to identify recharge and discharge zones, which is important for evaluating the role of different subwatersheds in sustaining aquifers or streams. (3) Routing Store capacity (mm), representing the response capacity of the watershed once water leaves the production store, accounting for delayed transfer through storage and release. High values indicate buffering by lakes or reservoirs, while low values show rapid responses in steep or small watersheds, relating this parameter to geomorphology and infrastructure influences on hydrological timing; and (4) Unit hydrograph time base (UH1, days), representing the time needed to convert effective rainfall into streamflow. It shows the watershed response speed, highlighting differences in watershed response to climate variability (Figure 9).

CemaNeige is a degree‐day‐based model designed to simulate snow accumulation and melt dynamics within a watershed, capable of simulating and quantifying lumped infiltration processes for specific watersheds [89]. It is a semi‐distributed model that divides the watershed into five elevation bands of equal area to account for altitudinal variability in snow processes. It requires only two free parameters, calibrated using historical meteorological data: (1) Snowpack coefficient (dimensionless), which represents the snowpack's thermal inertia, this is the fraction of solid precipitation retained in the snowpack per degree of cold content; and (2) Degree‐day melt coefficient (mm °C^−1^ day^−1^), which controls the rate of snowmelt.

#### 2.5.1 Model Inputs and Initialization

The coupled CemaNeige+GR4J model is forced using our daily gap‐filled precipitation and temperature databases, and PET is estimated using Oudin's method [70]. The partitioning of precipitation into rain and snow is based on a temperature threshold approach from CemaNeige's model, which is suitable for watersheds located below 1500 m a.s.l. This method uses daily minimum and maximum air temperatures to determine the fraction of precipitation falling as snow [90]. To avoid parameter biases due to initial conditions, a model warm‐up period was defined starting on 01/01/1961 (the beginning of the meteorological records) and continuing up to the first available streamflow records for each watershed. This ensured that the watershed‐specific parameters and snowpack conditions are stabilized before model calibration.

#### 2.5.2 Model Calibration

To estimate watershed‐specific parameters, the model is calibrated against historical streamflow using the local, gradient‐free optimizer of Michel et al. (2003) [91]. The algorithm explores a broad parameter space and refines possible sets via stepwise, performance‐based adjustments, with the objective of maximizing the Nash‐Sutcliffe Efficiency (NSE) between observed and simulated discharge. Since the primary objective of this study is to estimate hydrological parameters and analyze their regional trends, rather than simulate streamflow for predictive purposes, no separate validation period was defined. Although this calibration‐only approach is adequate for parameter estimation, it should be noted that the absence of independent validation may bias the model performance, often leading to an overestimation. To assess the reliability of estimated parameters, the NSE coefficient was computed for each watershed to verify that the model satisfactorily reproduces observed streamflow. A representative comparison of observed and simulated streamflow for watershed YAM9 is provided in Figure S4. We acknowledge that alternative efficiency metrics focusing on low‐flow conditions, such as the logarithmic NSE (NSElog), could provide complementary insight into baseflow and percolation dynamics, which are particularly relevant for groundwater recharge assessments. The use of NSElog is identified as a valuable extension for future work aimed at refining the evaluation of recharge‐related processes and low‐flow behavior.

### 2.6 Global Climate Change Impacts

In this study, we considered El Niño‐Southern Oscillation (ENSO) as a global reference of climate variability to assess its influence on the climate of Montérégie. ENSO is a global oscillatory climatic pattern (i.e. every 2–7 years) that occurs between ocean surface temperature (El Niño/La Niña) and atmospheric pressure (Southern Oscillation) in the equatorial region of the Pacific Ocean (120–170°W) [92]. As the largest ocean on earth, the Pacific exerts a global influence on temperature and precipitation patterns worldwide [93, 94].

The Oceanic Niño Index (ONI), established by NOAA, is used to track and recognize fluctuations in the oceanic part of the ENSO by recording the three‐month moving average of sea surface temperature anomalies in the central equatorial Pacific [95]. An ONI value above +0.5°C is associated with warm‐phase conditions (El Niño), while a value below −0.5°C is associated with cold‐phase conditions (La Niña). A teleconnection between the ENSO and the North Atlantic Oscillation (NAO) has been demonstrated [96]. According to NOAA, during El Niño winters, there is a high likelihood of warmer‐than‐normal temperatures in northwestern North America and increased rainfall in the southeastern region [95]. During La Niña phase, the opposite effects are usually observed, often favoring conditions for Atlantic hurricane formation. However, these climatic patterns become less consistent at higher latitudes, as the tropical effects of ENSO are attenuated by additional atmospheric and oceanic factors.

A cross‐correlation analysis between the monthly ONI values and the monthly anomalies of six hydrometeorological parameters (temperature, precipitation, intensity, streamflow discharge, simulated effective precipitation (Ps), and simulated percolation) was conducted to evaluate the impact of ENSO on the regional hydrological variability in Montérégie. Pearson's correlation coefficients (r) were calculated for different time lags between two time series. The maximum correlation coefficient and its associated lag, identified within an 84‐month window (corresponding to the typical ENSO cycle of approximately seven years), were extracted to determine the climatic teleconnection between this global phenomenon and the regional climate of Montérégie.

## 3 Results

### 3.1 MICE Imputations Results

Missing data were imputed using the MICE method for all hydroclimatic and piezometric records. Time series with Fraction of Missing Information (FMI) > 40% were excluded from further analysis due to high deviation between pre‐ and post‐imputation statistics. This included most groundwater level series before 2000 and a few records after 2000. In contrast, meteorological and hydrometric records showed low missing data rates (3%–4%) and acceptable FMI values (average <10%), and were retained in the analysis. Detailed results and quality metrics are provided in Supplementary Information.

### 3.2 Climate Data

#### 3.2.1 Temperature

Based on average daily climate records from 1980 to 2023 (Table 1), central Montérégie shows the highest temperatures, particularly in BAIE (between 6.7 and 7°C day^−1^) and RICH (between 6.7 and 6.8°C day^−1^) watersheds. On the contrary, YAM watershed shows the lowest temperatures (between 5.7 and 6.3°C day^−1^), while CHAT has the largest temperature variability (between 6 and 6.9°C day^−1^). A comparison between temperature and percentage of land cover (Table 2) suggests that higher proportions of cropland are generally related to higher temperatures (e.g., BAIE: 40%–90%; RICH: 65%–79%), whereas watersheds dominated by forest cover (55%–88%) show lower temperatures. However, this relationship is not unidirectional, while crops and vegetation adapt to the regional climate, they also modify local climate through changes in albedo, evapotranspiration, and soil moisture. The CHAT watershed (61%–81% forest) and the sub‐watershed YAM6 (69% cropland) do not fully follow this pattern and show no clear relationship between land cover and temperature.

#### 3.2.2 Precipitation

Yearly accumulated precipitation ranges between 950–1270 mm year^−1^, and the intensity varies between 5.6 and 6.7 mm day^−1^ (Table 1). Precipitation amount generally increases from west to east, being higher near the Appalachians. CHAT watershed receives the lowest precipitation (950–997 mm year^−1^), followed by RICH (973–1065 mm year^−1^) and BAIE (1096–1151 mm year^−1^), while YAM registers the highest values (1023–1270 mm year^−1^), decreasing from upstream (Appalachians) to downstream (St. Lawrence lowlands). Precipitation intensity follows a similar pattern, with lower values in CHAT (5.6–5.9 mm day^−1^) and higher values in YAM (6–6.7 mm day^−1^). A notable exception is RICH3 (6.4 mm day^−1^), located in central Montérégie, at the foothills of Mount Rougemont.

### 3.3 Climate Trends

#### 3.3.1 Temperature Trends

**FIGURE 3:**
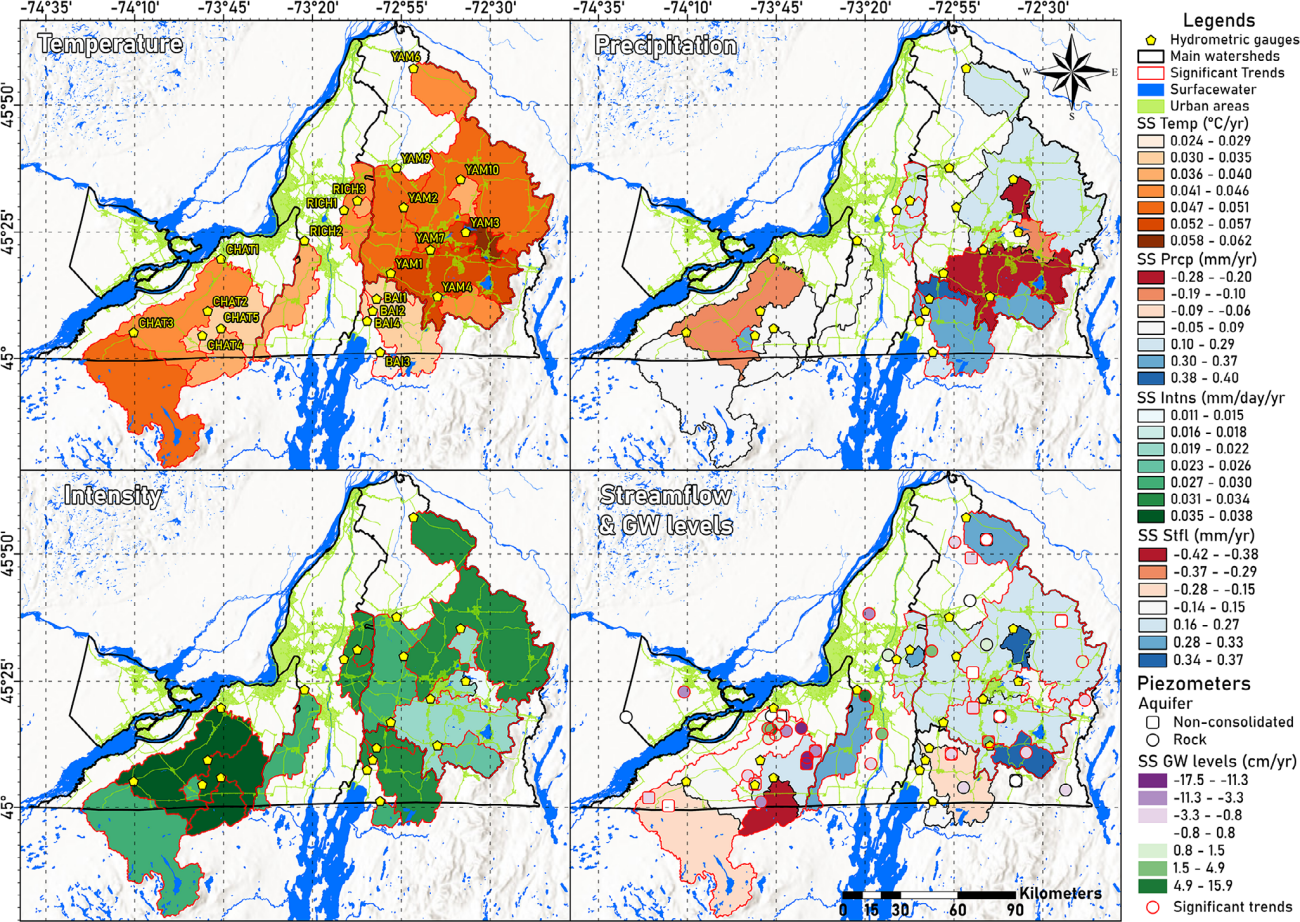
Results of the trends of the historical monthly anomalies (1980–2023) of temperature (upper‐left), precipitation (upper‐right), intensity (lower‐left), and discharge in the different gauged watersheds, as well as of the piezometric levels (lower‐right) in Montérégie. Watersheds (polygons) and piezometers (circles) showing significant trends (*p* < 0.05) are outlined in red. Ranges of calculated Sen's‐Slopes (SS) values are presented in color gradients.

**FIGURE 4:**
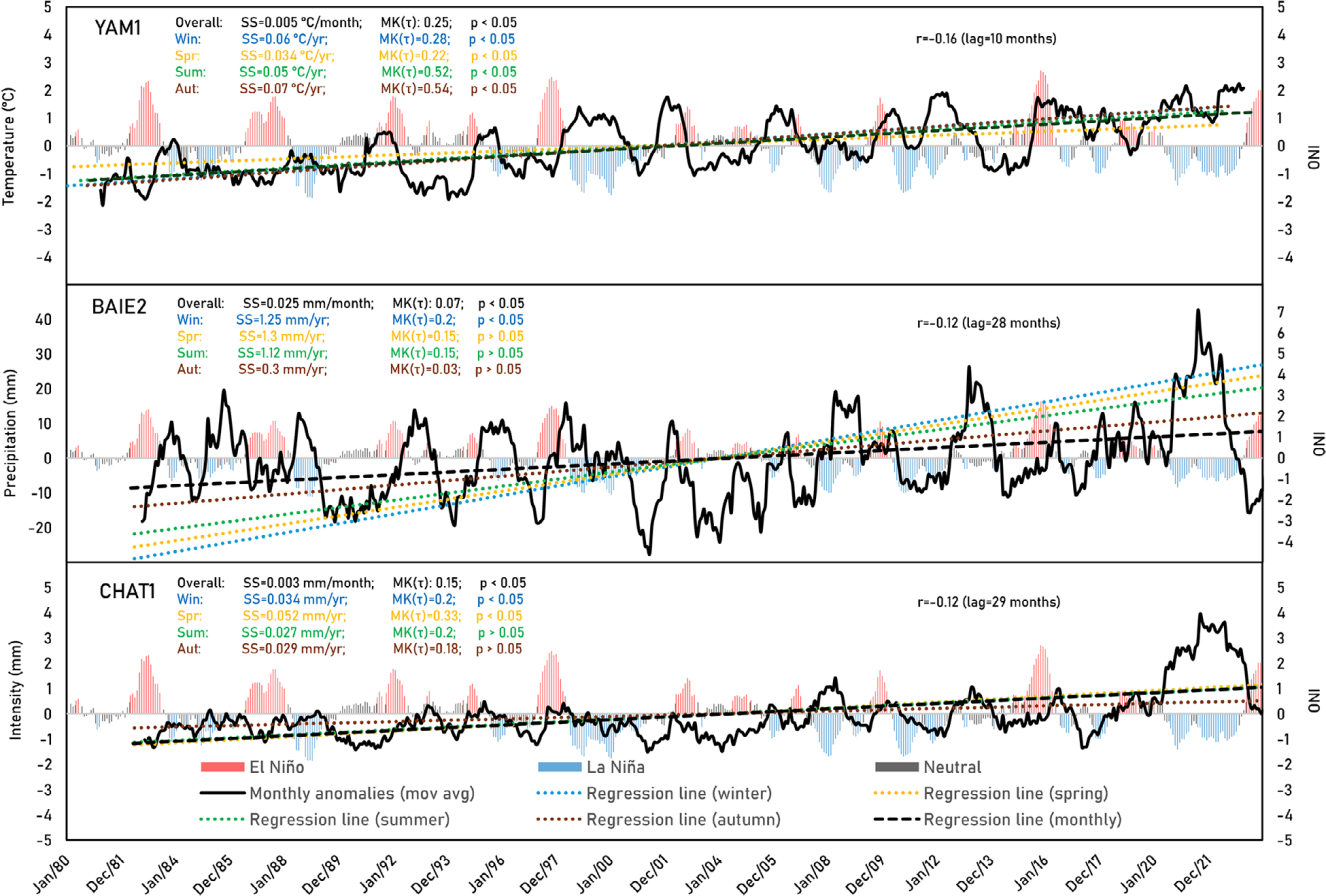
Monthly and seasonal anomalies of temperature (top), accumulated precipitation (middle), and precipitation intensity (bottom), for the watersheds with the highest SS in Montérégie (see Figure 3). For each watershed, the yearly moving average of the monthly anomalies (black straight line) and its linear regression (black dashed line); the seasonal anomalies and their linear regressions (colored dotted‐lines); as well as the magnitudes of their SS, MK(τ), and p values, are shown and compared with the monthly values of the Oceanic Niño Index (ONI). Each time‐series is phase‐shifted by the lag that produces the highest Pearson correlation (r).

Monthly temperature anomalies show significant (*p* < 0.05) increasing overall trends across Montérégie (Figure 3, top‐left), with low‐intermediate magnitudes: MK(τ) values ranging from 0.1 to 0.3 (Table S2.1) and Sen's Slope (SS) estimates between 0.024 and 0.06°C per year (Table S2.2). The highest SS values are upslope and midslope of YAM watershed (Figure 4, top), as well as upslope of CHAT watershed, while BAI and the east of CHAT watersheds have the smallest SS.

**FIGURE 5:**
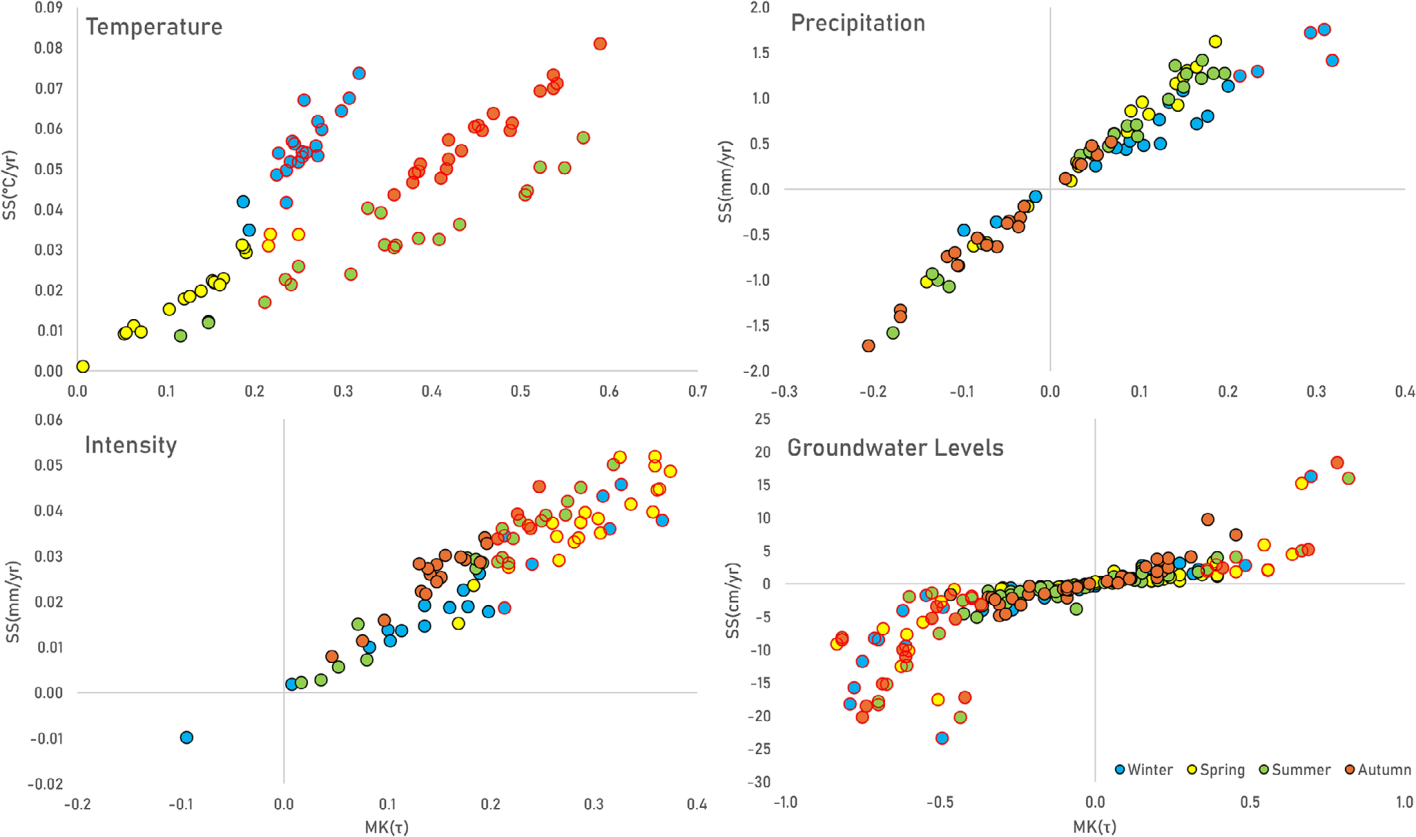
Relationships between MK(τ) and SS computed from seasonal anomalies of temperature (top‐left), precipitation (top‐right), intensity (bottom‐left), and groundwater levels (bottom‐right) across Montérégie watersheds (1980–2023). Each circle represents a watershed, colored by season. Circles outlined in red indicate significant trends (*p* ≤ 0.05).

Analysis of seasonal temperature anomalies shows that most watersheds do not present significant warming trends during spring (Figure 5 (top‐left), Tables S2.1 and S2.2). In contrast, the most significant warming trends with an intermediate‐strong magnitude (highest MK(τ) values) are observed during autumn and summer (Figure 4 (top), green and brown lines; Tables S2.1 and S2.2). BAIE watershed is an exception, showing weaker and non‐significant (*p* > 0.05) temperature trends during summer (*n* < 23). During winter, most watersheds show significant temperature trends (*p* < 0.05) with high SS values (Table S2.2). However, these trends have weaker magnitudes (lower MK(τ) values) compared to autumn and summer (Figure 5, top‐left). This indicates increasing temperature fluctuations in winter, which have a significant impact on the dynamics of snow and ice melt and affect the processes of infiltration and surface runoff. PET (directly dependent on temperature) shows similar seasonal patterns, with strong and significant trends during autumn and summer in all watersheds except BAIE (Tables S2.3 and S2.4). However, PET trends remain non‐significant and very weak during winter and spring, likely due to persistent freezing temperatures and vegetation dormancy, which limit evapotranspiration processes during these colder periods. Subwatersheds CHAT3, YAM1, YAM3, YAM7, and YAM9 are exceptions, as they are the only ones that show significant increasing trends (*p* < 0.05) of weak‐intermediate magnitude for both temperature and PET in spring (Tables S2.3 and S2.4).

In contrast to the last frost of the year (in winter‐spring), the first frost (in autumn‐winter) shows a significant and stronger trend to occur later across Montérégie (Figure 10 (top), Tables S2.3, and S2.4). This delayed onset of frost has significantly lengthened the duration of the non‐freezing season, particularly in CHAT3, YAM1, YAM2, and YAM3 watersheds (MK(τ): 0.2–0.3; SS: 0.45–0.6 days year^−1^). Regarding the vegetation growing season, only its end (in autumn‐winter) tends to occur later in the year (Figure 10, top), and this shift is mainly observed in gauged subwatersheds of the eastern part of Montérégie, such as YAM, RICH1, and RICH3 (Tables S2.3 and S2.4). The YAM3 subwatershed is the only one showing a significant increasing intermediate trend in the vegetation growing season length (MK(τ): 0.2; SS: 0.5 days year^−1^; *p* < 0.05).

#### 3.3.2 Precipitation Trends

Although the overall trends of monthly precipitation anomalies show only a slight increase in the amount of precipitation during the last decades (Figure 4, middle), they show significant trends (*p* < 0.05) in some of Montérégie's gauged watersheds (Figure 5, top‐right). Weak increasing trends are found in BAI, CHAT4, RICH1, RICH3, and YAM4 watersheds (MK(τ): 0.06–0.1; SS: 0.24–0.36 mm year^−1^). In contrast, weak decreasing trends are found in the upslope region of YAM1 (MK(τ): ‐0.07; SS: ‐0.24 mm year^−1^). The seasonal anomalies show that moderate increasing trends of precipitation only occur in winter (Figure 5, top‐right), particularly in the catchment areas of BAIE, CHAT4, and YAM6 gauging stations (MK(τ): 0.2–0.3; SS: 1.3–1.8 mm year^−1^). Weak decreasing trends, although not statistically significant (*p* > 0.05), predominantly appear during summer and autumn (Tables S2.5 and S2.6).

Monthly precipitation intensity anomalies show significant weak increasing trends across most of Montérégie (MK(τ): 0.1‐0.2; SS: 0.002–0.003 mm day^−1^), except in the smaller upslope watershed YAM3 (Figure 3, bottom‐left). The seasonal anomalies also show significant weak‐intermediate increasing trends in intensity (Figure 4 (bottom), and Figure 5 (bottom‐left)), especially in spring (MK(τ): 0.2–0.4; SS: 0.03–0.05 mm year^−1^), although some significant trends also occur in summer in the BAIE, upslope CHAT regions, and eastern RICH and YAM watersheds (MK(τ): 0.2–0.3; SS: 0.03–0.05 mm year^−1^). A few watersheds show significant increasing intensity trends in winter and autumn (Tables S2.5 and S2.6).

**FIGURE 6:**
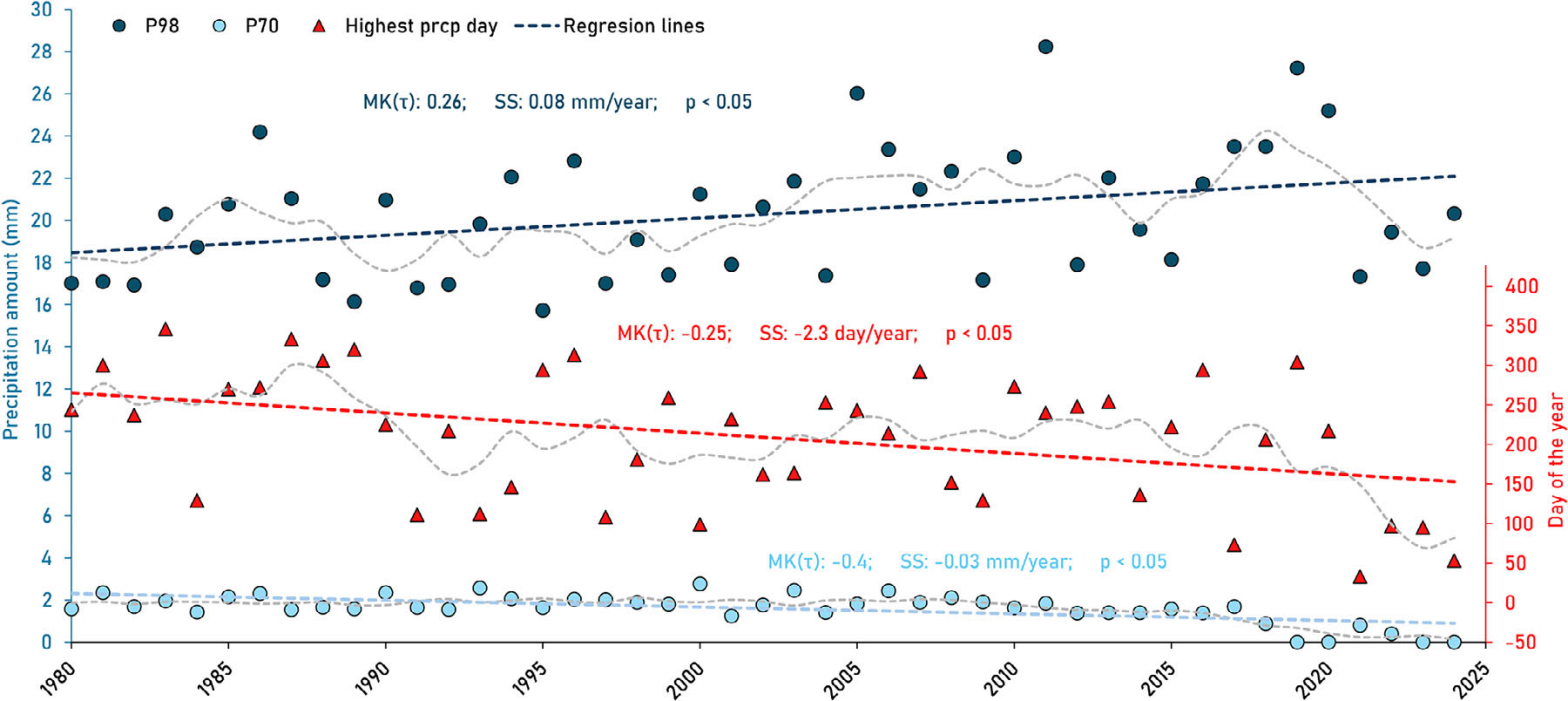
Yearly percentiles of high (P98) and low (P70) precipitation amounts for watershed CHAT1 (circles). The days with the highest amount of precipitation per year are also indicated (triangles). Linear regressions and trend statistics (SS, MK(τ) and *p*) are also shown. Gray dashed‐lines represent five‐years moving averages.

Yearly values of P70 show significant weak‐intermediate decreasing trends (*p* < 0.05) in roughly half of the gauged watersheds, mainly in CHAT, RICH2, and mid‐upstream YAM (MK(τ): ‐0.4 to ‐0.2; SS: ‐0.03 to ‐0.01 mm year^−1^). In contrast, the yearly values of P98 show significant weak‐intermediate increasing trends in most of the watersheds, mainly in BAIE, CHAT, RICH, mid‐downstream YAM (MK(τ): 0.2–0.4; SS: 0.08–0.14 mm year^−1^) (Tables S2.5 and S2.6). This suggests an ongoing shift toward fewer moderate‐intensity precipitation events (potentially reducing infiltration) and a rise in higher‐intensity events (potentially enhancing runoff). The analysis of the timing of the annual highest precipitation shows a significant tendency to occur earlier in CHAT1, CHAT3, and CHAT4 watersheds (MK(τ): ‐0.3 to ‐0.2; SS: ‐3.1 to ‐2.2 days year^−1^) (Figure 6). BAIE watershed also presents a slight tendency to occur earlier, while the rest of the watersheds show slight tendencies to occur later, although not significantly (*p* > 0.05). Overall, anomalies in climate variables (temperature, precipitation amount, and intensity) have notably intensified since the 1990s (Figure 4).

### 3.4 Streamflow Characteristics

Historical averages of daily Q (mm day^−1^) show higher discharge (>1.5 mm day^−1^) in watersheds with steep slopes (Table 1). YAM watersheds show the highest average discharge (1.4–1.9 mm day^−1^), followed by BAIE (1.3–1.5 mm day^−1^), RICH (1.1–1.8 mm day^−1^), and CHAT (1.2–1.5 mm day^−1^). Notably, RICH3 watershed, despite its small area (35 km^2^), shows particularly high discharge (1.8 mm day^−1^), likely related to runoff from steep slopes around Mount Rougemont (Table 1 and Figure 1). Although spatial negative correlations between mean annual Q and the percentage of wetland+surface‐water area have been reported in southern Quebec, this pattern does not occur in Montérégie [97]. Our results show that both high and low Q values (Table 1) are found in watersheds with high percentages of wetlands and surface water bodies (Table 2) [97].

#### 3.4.1 Streamflow Trends

Monthly Q anomalies show weak but statistically significant (*p* < 0.05) increasing trends in half of Montérégie's gauged watersheds (Figure 3, bottom‐right), mainly in mid‐downstream section of CHAT, central RICH, and YAM (MK(τ): 0.06 – 0.12; SS: 0.12 – 0.36 mm year^−1^). Weak decreasing trends occur only in the CHAT upslope watersheds (CHAT3, CHAT4, CHAT5) and in the BAI2 upslope watershed, but these are not significant (MK(τ): ‐0.08 to ‐0.02; SS: ‐0.48 to ‐0.12 mm year^−1^). The findings highlight notable spatial contrasts, with downslope gauges in CHAT showing significant increases in discharge, despite decreasing trends observed at upstream locations. Although previous studies have shown decreasing trends in mean annual streamflow across southern Quebec, more recent analyses reveal both increasing and decreasing trends, with increasing trends being more dominant, in agreement with the overall trends observed in this study (Figure 3, bottom‐right) [97, 98]. These analyses also report a significant upward trend at the downstream station CHAT1, which reinforces our obtained trends.

**FIGURE 7:**
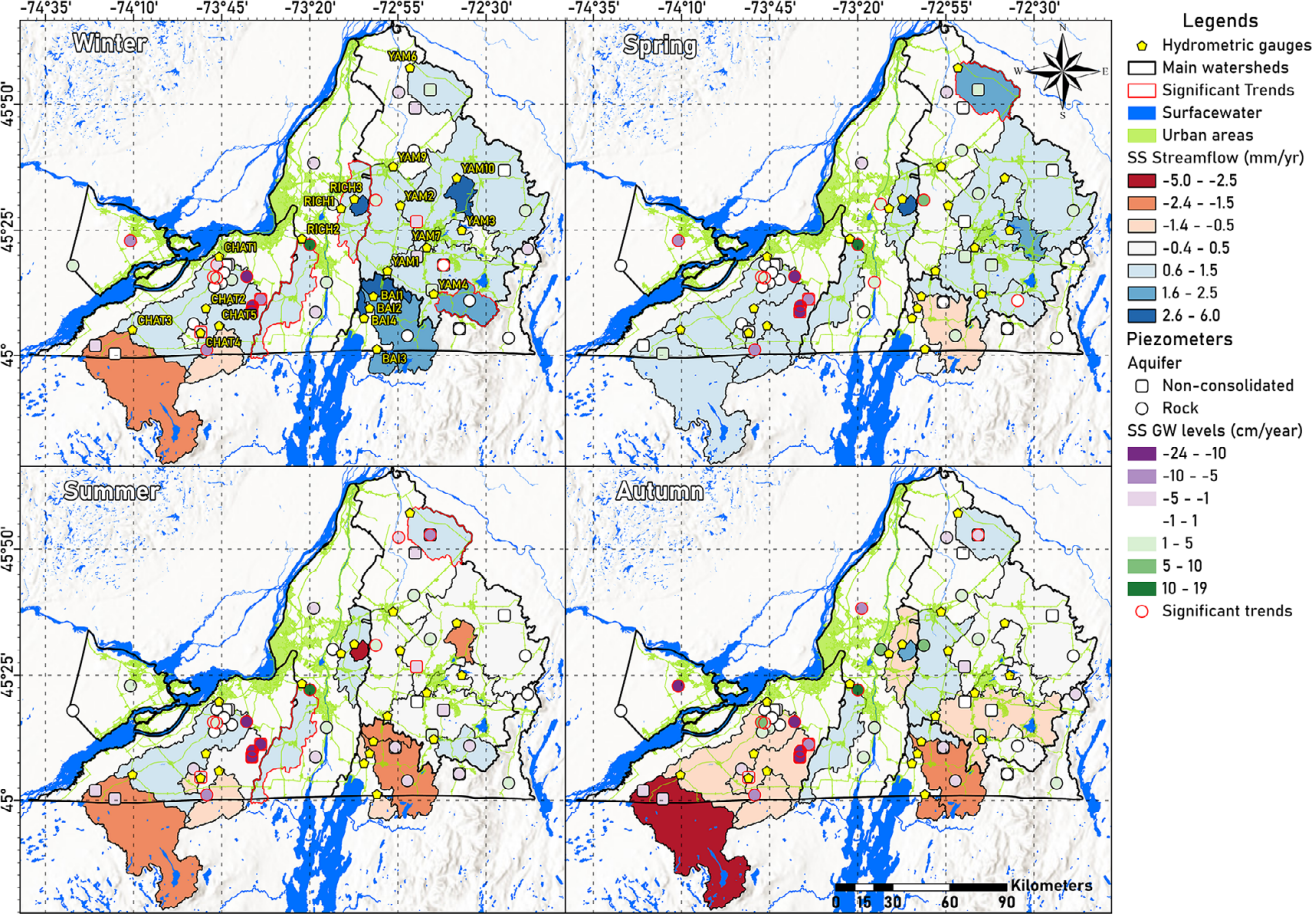
Historical seasonal trends for streamflow and groundwater levels in Montérégie, based on MK(τ) values. Watershed's streamflow trends (polygons) are represented by red‐blue color gradients, while piezometer's (symbols) level trends are represented by purple‐green color gradients. Piezometers are differentiated between those that measure levels from rocky (circles) or non‐consolidated (squares) aquifers, as well as those in which a short or constant anthropic influence is observed (red outline), or undetermined (orange outline).

**FIGURE 8:**
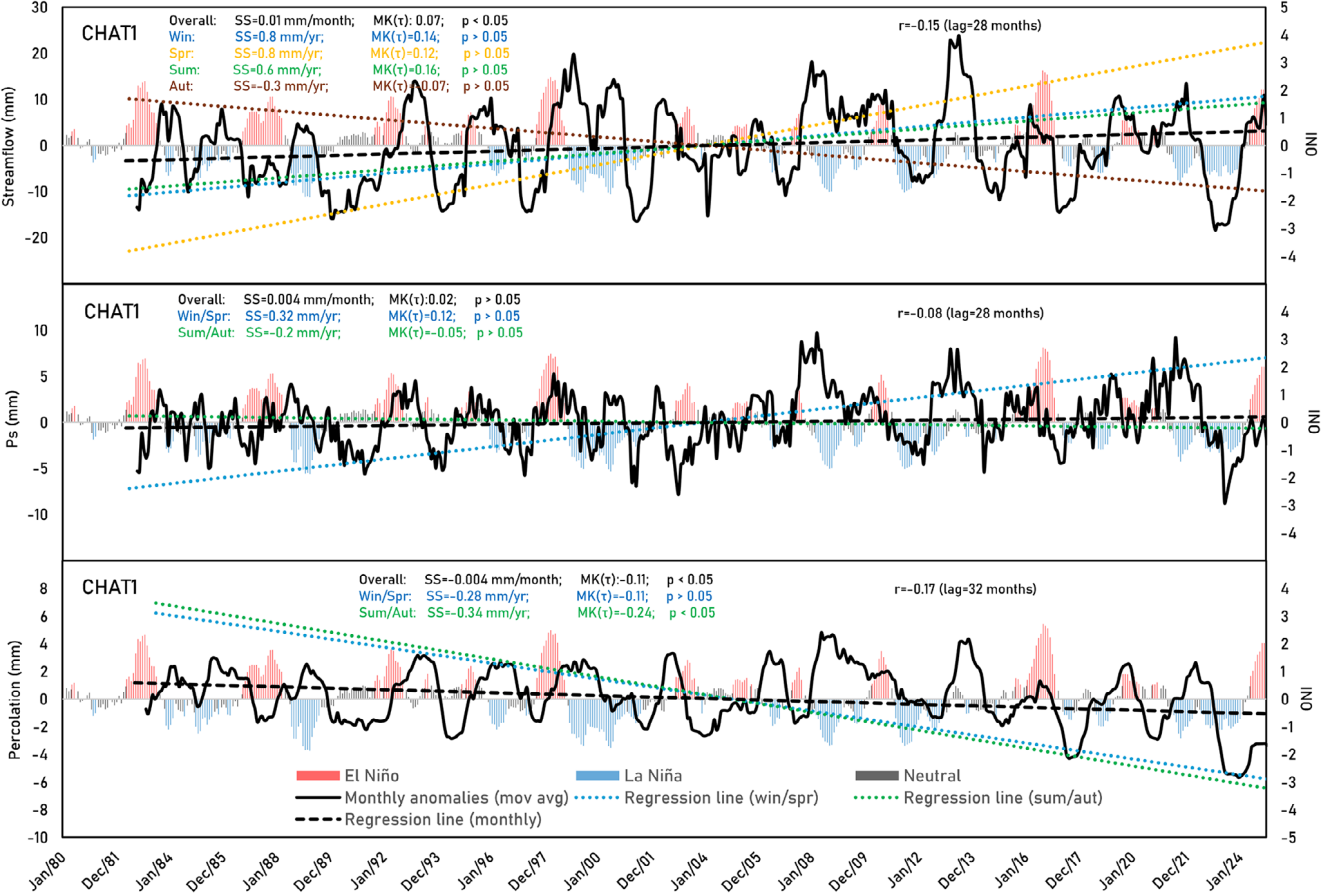
Monthly and seasonal anomalies of streamflow (top), simulated GR4J accumulated Ps (middle), and percolation (bottom), for one of the watersheds with the longest records (CHAT1). The yearly moving average of the monthly anomalies (black straight line) and its linear regression (black dashed line); the seasonal anomalies and their linear regressions (colored dotted‐lines); as well as the magnitudes of their SS, MK(τ), and *p* values, are shown and compared with the monthly values of the Oceanic Niño Index (ONI). Each time‐series is phase‐shifted by the lag that produces the highest Pearson correlation (r).

The seasonal Q anomalies show regionally varying trends (Figure 7), with some subwatersheds showing significant overall (*p* < 0.05), but no seasonal (*p* > 0.05), trends (Figure 8, top). Winter shows the highest regional increase in Q (Figure 7, top‐left), significantly in RICH1, RICH2, and YAM4 (MK(τ): 0.2–0.3; SS: 1.2–2 mm year^−1^). Only upslope catchments, CHAT3, CHAT4, and CHAT5 show non‐significant decreasing trends during winter. In spring, most watersheds show increasing Q trends, but they are only significant in downstream YAM6 (MK(τ): 0.2; SS: 1.8 mm year^−1^). Only upstream BAIE watersheds show non‐significant decreasing trends during spring (Figure 7, top‐right).

During the winter‐spring period, Q95 significant trends increase with a weak magnitude in RICH2 and YAM8 (MK(τ): 0.2; SS: 0.6 mm year^−1^), while the same trends are found for Q5 in CHAT1, CHAT2, RICH2, and YAM4 (MK(τ): 0.2–0.4; SS: 0.002–0.007 mm year^−1^). Non‐significant (*p* > 0.05) and weak decreasing trends for both Q95 and Q5 appear mostly in upslope gauged watersheds. During this period, upslope YAM4 is the only subwatershed that shows a significant delay in the time of the lowest annual discharge (MK(τ): 0.3; SS: 1.1 days year^−1^) (Tables S2.7 and S2.8).

During the summer‐autumn period, significant (*p* < 0.05) and gentle increasing overall trends of Q are observed only in downstream watersheds RICH2 and YAM6 (MK(τ): 0.25; SS: 0.84–0.88 mm year^−1^), while most decreasing trends in BAIE, upstream CHAT, and YAM10 are not statistically significant (MK(τ): ‐0.3 to ‐0.03; SS: ‐4.8 to ‐0.1 mm year^−1^) (Figure 7, bottom). Summer‐autumn Q95 significant trends weakly increase only in YAM6 (MK(τ): 0.2; SS: 0.04 mm year^−1^), while Q5 significant trends weakly increase only in RICH1 (MK(τ): 0.2; SS: 0.006 mm year^−1^). Additionally, CHAT1, RICH1, and RICH2 show a delayed timing of the lowest discharge events (MK(τ): 0.3–0.4; SS: 0.9–1.2 days year^−1^) (Tables S2.7 and S2.8).

Previous studies have reported increasing and decreasing trends in streamflow and in the timing of extreme flow events across southern Quebec [12, 13, 25, 98, 99]. Similarly, our results show that such variable trends are also observed in Q, Q5, and Q95 at the regional scale of Montérégie, although not all are statistically significant (*p* > 0.05). Our precipitation analysis shows no significant regional increase in total precipitation over time, but rather an increase in precipitation intensity (Figure 3, bottom‐left), which appears to be more closely related to the observed Q trends, along with other factors such as snow melt due to warmer winters, land cover, soil type, and topographic slope.

According to these trends, warming temperatures, altered precipitation patterns, and shifting streamflow regimes are reshaping the regional water cycle in Montérégie. Strongest warming occurs in summer, autumn, and also in winter, increasing snow and ice melting, reducing snowpack storage, and altering spring recharge. The delayed start of frost and a longer non‐freezing season extend evapotranspiration and modify recharge timing. While total precipitation shows only weak increases, intensity trends are significant, especially in spring, favoring more extreme events. This enhances runoff and reduces infiltration potential, limiting groundwater recharge efficiency. These changes can be noticed in streamflow behavior, since downstream watersheds show significant increases related to intensified precipitation and warmer winters, while some upslope catchments show stable or declining flows.

### 3.5 Groundwater Levels Trends

Monthly groundwater level anomalies show both significant (*p* < 0.05) increasing and decreasing overall trends across Montérégie (Figure 3, bottom‐right), many of them being strong (|MK(τ)| > 0.5). However, seasonal anomalies show that, although overall trends are either increasing or decreasing, some of these patterns are not consistent across all seasons of the year (Figures 5 and 7).

The eastern region of CHAT shows the strongest decreasing trends of all of Montérégie. In contrast, the northern part of the watershed shows strong increasing trends (Figure 3, bottom‐right). In both regions, the trends are constant during all seasons of the year. Meanwhile, the southwestern upslope region of CHAT shows increasing trends during spring but decreasing trends during the rest of the year, although not significant (Figure 7).

The central watershed of RICH shows the strongest increasing overall trends of all Montérégie (Figure 3, bottom‐right). Seasonal analysis reveals that these increases are especially prominent during spring (Figures 5 and 7). In contrast, the northern RICH area shows significant overall decreasing trends, with declines observed during all seasons of the year, particularly intensified in autumn. Similarly, significant overall decreasing trends occur in the southern RICH area, with pronounced seasonal decreases in winter and summer, but slight increasing in the autumn (Tables S2.11 and S2.12).

The BAIE watershed shows significant overall decreasing trends in the east and west regions (Figure 3, bottom‐right). Seasonal trends show that the groundwater levels in the western area decline mainly in winter, while in the eastern region, the decline is most notable in autumn. However, both regions show increasing trends in spring (Figure 7).

In the YAM watershed, significant overall increasing trends are observed in the eastern, western, and southern regions (Figure 3, bottom‐right). These increases are particularly strong in winter and spring, notably significant in the western region (Figure 5 (bottom‐right) and Figure 7). The remaining piezometers in YAM generally show significant decreasing trends. In the southern region, the only piezometer with significant declines during spring is observed. Central areas experienced significant decreases primarily in winter and summer, while the downstream northern area shows decreasing trends during summer and autumn. Spring is the season least affected by declines and in some cases even shows an upward trend.

In the VS watershed, one piezometer located in the unconfined sandy aquifer to the east showed an overall significant decreasing trend (Figure 3, bottom‐right). The seasonal trends indicate significant decreases in winter and autumn, which are even more pronounced in spring, while there are no significant increasing trends in summer. To the west, slightly increasing trends can be observed, mainly in winter, although these are not statistically significant (Figure 7).

Streamflow and groundwater levels trends results show that, when baseflow is used as a proxy for groundwater recharge, streamflow trends (i.e., baseflow) sometimes diverge from groundwater level trends recorded by piezometers in the same watershed (Figure 7) [19, 35]. This mismatch highlights the spatial variability of recharge within a single catchment, potentially driven by local topography, geology, and human activities, as has been demonstrated by studies showing the strong influence of shallow/local flow systems on stream‐aquifer exchanges [51, 52]. For example, gentle slopes limit lateral flow and promote localized recharge areas; variability in sediment thickness and aquifer confinement controls storage capacity and response times; and anthropogenic impacts like pumping or land cover changes can locally modify recharge–discharge dynamics.

We also evaluated trends in the timing of groundwater level peaks. Only one piezometer located in the central part of the CHAT watershed (CHAT9) shows a significant trend toward later occurrence during the winter‐spring period (0.49 days year^−1^). In the summer‐autumn period, two piezometers located in the central‐northern (YAM4) and northern (YAM11) regions of the YAM watershed show significant trends toward later occurrence, with rates of 0.52 and 0.64 days year^−1^, respectively (Tables S2.11 and S2.12).

### 3.6 Effective Precipitation and Percolation in the GR4J Model

#### 3.6.1 Simulation Parameters, Effective Precipitation (Ps), and Percolation

The NSE values range from 0.46 to 0.8 (Table 3), which, according to Motovilov et al. (1999), indicate satisfactory to good simulation performance [100]. The lowest NSE value corresponds to the YAM3 subwatershed, which contains the “Barrage Choinière” dam. Since streamflow discharge in this watershed is primarily controlled by human operations rather than rainfall‐runoff processes, the GR4J model is less capable of reproducing accurately the observed flow dynamics.

**TABLE 3:** Simulation results of the GR4J model after Michels' calibrations [91]. The Nash‐Sutcliffe Efficiency (NSE) criterion is shown to evaluate the accuracy of the simulations, as well as the calibrated modeling parameters for each watershed: maximum capacity of the production store (mm), groundwater exchange coefficient (mm), one day ahead maximum capacity of the routing store (mm), and the time base of unit hydrograph UH1 (days). Also shown are the average accumulated yearly outputs of simulated effective precipitation (Ps) and percolation results for the winter‐spring, summer‐autumn, and full year periods. For a clearer comparison of values, color gradients have been applied by columns (red=positive, blue=negative).

Watershed	NSE	Production store	GW exchange	Routing store	UH1	Percolation (mm)			Ps (mm)		
						Yearly	Win/Spr	Sum/Aut	Yearly	Win/Spr	Sum/Aut
BAIE2	0.73	242	−0.40	62	1.91	164	123	42	464	168	295
BAIE3	0.61	299	−1.05	24	1.52	171	128	43	484	180	302
BAIE1	0.66	174	−0.94	49	1.50	136	101	33	428	147	280
BAIE4	0.57	153	−0.29	32	1.42	122	92	30	418	137	280
CHAT5	0.78	187	0.36	46	2.19	107	85	21	394	135	256
CHAT4	0.74	209	−0.35	53	1.36	121	98	22	423	147	273
CHAT2	0.75	162	−0.01	40	2.19	101	79	21	371	122	248
CHAT1	0.78	217	0.33	51	2.29	115	92	24	391	139	252
CHAT3	0.72	523	0.62	56	2.10	169	123	47	480	204	276
RICH3	0.65	67	0.43	23	1.46	70	52	18	338	97	240
RICH2	0.66	154	−0.20	26	2.21	101	80	21	374	122	252
RICH1	0.72	144	−0.03	27	1.62	110	84	25	387	124	262
YAM4	0.75	198	−0.06	30	1.95	161	116	45	434	150	284
YAM3	0.46	380	0.13	215	2.22	205	145	59	493	196	296
YAM1	0.77	240	0.03	55	2.13	161	119	42	436	159	278
YAM7	0.68	340	0.44	67	1.61	189	137	51	483	186	297
YAM2	0.78	169	−0.01	33	2.22	128	96	32	395	132	264
YAM9	0.80	206	0.04	36	2.29	143	110	33	427	149	277
YAM10	0.61	112	0.00	30	1.52	99	75	25	362	116	246
YAM6	0.67	151	0.33	40	2.08	108	82	26	380	120	260

**FIGURE 9:**
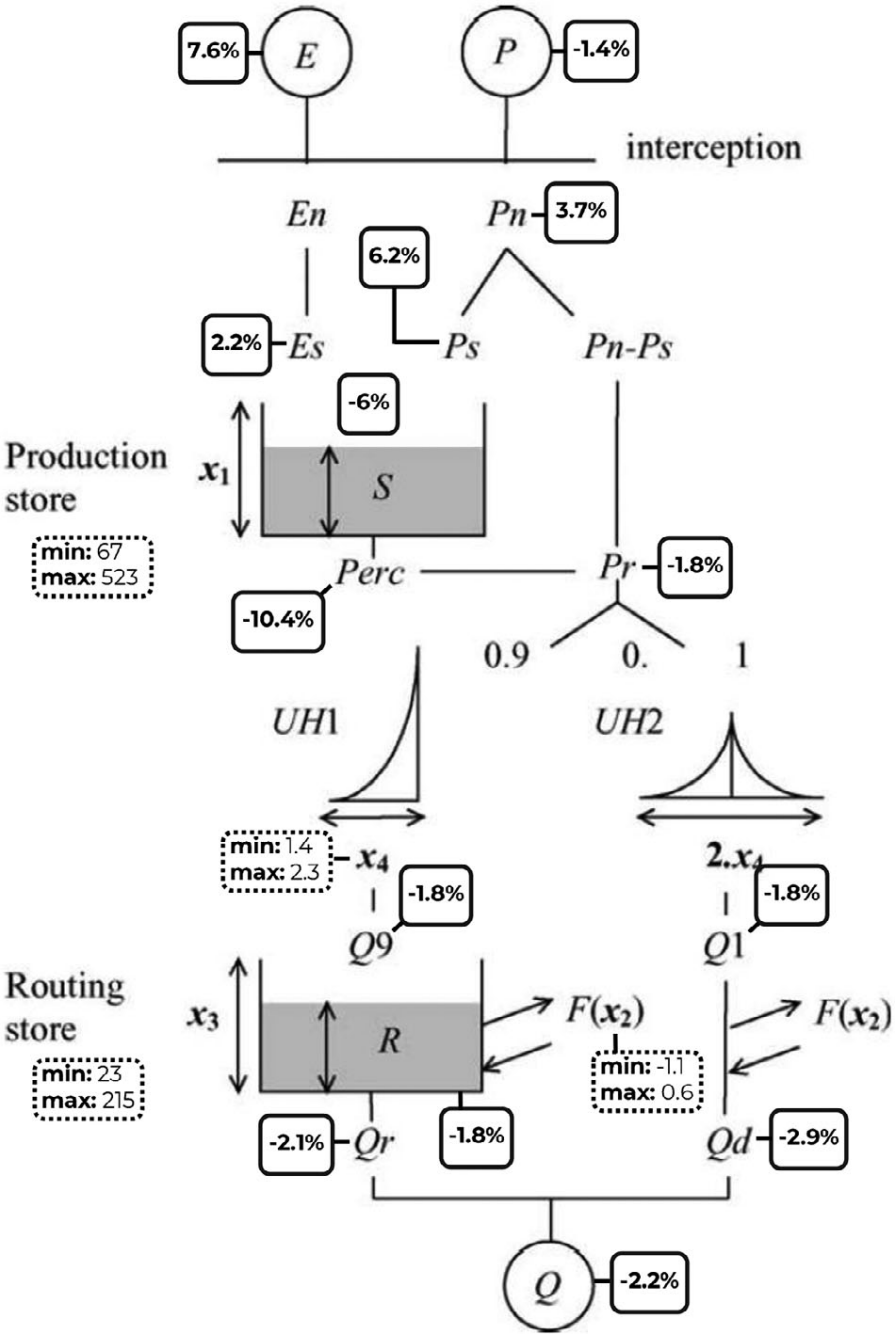
Modified GR4J model flow diagram (adapted from Perrin et al., 2003), illustrating regional decadal changes in simulated hydrological variables over the instrumented watersheds domain of Montérégie. Changes correspond to the percentage difference between area‐weighted decadal mean values for 1980–1989 and 2013–2022, computed using non‐overlapping instrumented watersheds. Fluxes are based on yearly totals, whereas the production (S) and routing (R) stores are based on yearly mean values. Dotted‐line boxes show the range (min‐max) of calibrated GR4J parameters (x1–x4; Table 3).

The Production Store parameter, which represents the maximum soil moisture capacity in the catchment, shows substantial variability across Montérégie, with values ranging from 67 to 523 mm (Table 3 and Figure 9). The highest values (> 200 mm) are found in the upslope subwatersheds of CHAT, BAIE, and YAM, where the land cover is predominantly forested, soils are primarily till, and Quaternary sediments consist mainly of undifferentiated till (Figure 2 and Table 2). The GW exchange parameter, representing the net flux between the watershed and the underlying aquifer, ranges from −1.05 to 0.62. Considerably negative values (<−0.1) are observed in the BAIE watershed and in subwatersheds CHAT4 and RICH2, suggesting significant groundwater flow from the watershed to the aquifer. Conversely, most other subwatersheds show strongly positive values (>0.1), suggesting constant groundwater discharge into the streamflow. Values near zero (between −0.1 and 0.1) are found in CHAT2, RICH1, and the upslope of YAM, showing limited exchange.

The Routing Store parameter, which represents the capacity of the routing storage and thus the delay in water transfer once it leaves the production store, ranges between 23 and 67 mm, except in YAM3, where an exceptionally high value (215 mm) is observed (Table 3 and Figure 9), attributed to delayed water storage and release caused by the dam management. High values (> 40 mm) are also observed in the CHAT watershed, in upslope regions with lakes or dams (e.g., upstream BAIE and YAM), and in YAM6, showing delayed streamflow response to baseflow. Conversely, lower values are found in the central zone of RICH and in small, steep subwatersheds, where streamflow responds faster to baseflow changes. The UH1 parameter, which is the unit hydrograph time base and shows how quickly effective precipitation is converted to streamflow, ranges from 1.4 to 2.3 days. Faster responses to precipitation are seen in the BAIE watershed, near Mont Rougemont (RICH1 and RICH3), and in small, steep subwatersheds such as CHAT4, YAM4, YAM7, and YAM10. The remaining CHAT and YAM subwatersheds show slower precipitation responses (> 2 days).

Simulated yearly averages of effective precipitation (Ps) reaching the Production Store range from 338 to 493 mm, with higher values (> 400 mm) in the BAIE watershed, the upslope regions of CHAT, and the mid‐upslope regions of YAM (Table 3). Seasonally, Ps values are approximately twice as high in summer/autumn season (240–303 mm) compared to winter/spring season (97–204 mm). Simulated yearly averages of percolation range from 70 to 205 mm, with higher values (> 120 mm year^−1^) occurring in the same regions where Ps is high. In contrast to simulated Ps, percolation is about three times greater during the winter/spring season (52–145 mm) than during the summer/autumn season (18–59 mm). This indicates that approximately 70% of Ps becomes percolation in winter/spring, whereas only about 12% do so in summer/autumn (Table 3).

#### 3.6.2 Simulated Percolation and Effective Precipitation Trends

Despite the simulated monthly Ps anomalies showing limited variability (Figure 8, middle), significant Ps trends are present in only a few subwatersheds. Statistically significant overall trends in Ps anomalies were found in just three subwatersheds, RICH1, YAM2, and YAM4, showing very weak increasing trends (MK(τ): 0.1; SS: 0.12 mm year^−1^). During the winter‐spring season, significant seasonal trends are present in three subwatersheds: BAIE3 and YAM4 showed intermediate increasing trends (MK(τ): 0.3; SS: 0.6–1.9 mm year^−1^), while RICH3 showed a strong decreasing seasonal trend (MK(τ): ‐0.5; SS: ‐2.2 mm year^−1^). In contrast, during the summer‐autumn season, only RICH1 and YAM2 showed significant increasing seasonal trends in Ps, with weak to intermediate magnitudes (MK(τ): 0.2; SS: 0.6–0.9 mm year^−1^). These results indicate that simulated Ps has remained stable across Montérégie, with only localized and season‐specific changes. This stability suggests that climate inputs to the hydrological system are more strongly controlled by interannual variability than by long term changes.

Simulated monthly percolation anomalies presented greater historical variability than Ps (Figure 8, bottom), with more than half of the subwatersheds showing significant overall trends. Increasing simulated percolation trends were observed in the BAIE watershed and in YAM4, with weak to intermediate magnitudes (MK(τ): 0.06–0.2; SS: 0.24–2.4 mm year^−1^). Decreasing trends were present across several CHAT and YAM subwatersheds, although with generally weak magnitudes (MK(τ): ‐0.16 to ‐0.07; SS: ‐0.012 to ‐0.12 mm year^−1^). Seasonal analysis of percolation anomalies showed that, in the winter‐spring period, significant increasing trends occurred only in the BAIE watershed, with intermediate to strong magnitude (MK(τ): 0.4; SS: 1.4–1.7 mm year^−1^). During the summer‐autumn season, significant decreasing percolation trends were identified in CHAT1, CHAT3, YAM1, YAM3, and YAM8, with magnitudes ranging from intermediate to moderately strong (MK(τ): ‐0.4 to ‐0.24; SS: ‐1.3 to ‐0.09 mm year^−1^) (Figure 8, bottom). These results suggest a seasonal redistribution of percolation, with increasing winter‐spring recharge in BAIE and decreasing summer‐autumn recharge in subwatersheds of CHAT and YAM.

### 3.7 Hydrology and ONI Correlation

The cross‐correlation analysis between the ONI and hydroclimatic parameters of Montérégie shows relatively low Pearson coefficients ranging between −0.23 and 0.23, indicating weak to moderate correlation (Table 4). The hydroclimatic parameters showing the highest correlations are temperature (−0.18–0.23), simulated percolation (−0.23–0.22), and streamflow (−0.17–0.18). Conversely, simulated Ps presents the lowest correlations (−0.11–0.07).

**TABLE 4:** Values of the strongest correlation (p), and the lag time (months), achieved between the monthly anomalies of the hydroclimatic parameters computed in this study and the ONI values, during a time window of 84 months. For each parameter's columns, positive correlations are shown in blue‐gradient colors, and negative correlations are shown in red‐gradient colors.

Watershed	Best correlation (p)						Lag (months)					
	Temp	Prcp	Intns	Stfl	Ps	Perc	Temp	Prcp	Intns	Stfl	Ps	Perc
BAIE2	0.21	−0.12	−0.11	−0.13	−0.08	−0.19	36	28	−24	29	28	−25
BAIE3	0.22	−0.12	−0.11	0.15	−0.09	0.19	36	28	−25	−17	30	−41
BAIE1	0.21	−0.13	0.11	−0.13	−0.10	−0.21	35	28	−42	26	28	25
BAIE4	0.22	0.12	0.11	−0.16	−0.06	−0.17	35	−42	−9	28	28	−24
CHAT5	−0.18	−0.11	0.12	0.18	−0.07	−0.22	12	−30	18	18	−30	12
CHAT4	0.18	−0.12	0.13	−0.15	−0.07	−0.23	35	−4	17	42	3	12
CHAT2	0.17	−0.10	−0.08	−0.15	0.07	−0.13	36	28	35	27	18	27
CHAT1	0.16	−0.11	−0.12	−0.15	−0.08	−0.17	36	33	29	28	28	32
CHAT3	0.18	−0.15	0.14	−0.16	−0.09	−0.23	35	−4	17	40	42	28
RICH3	0.22	−0.12	−0.10	−0.14	−0.09	−0.15	35	−31	3	28	−31	−27
RICH2	0.17	−0.10	0.07	−0.13	−0.06	−0.16	36	30	−10	28	34	32
RICH1	0.18	−0.14	−0.12	−0.17	−0.07	−0.16	36	30	33	28	30	32
YAM4	−0.15	−0.12	−0.09	−0.16	−0.06	−0.15	10	30	34	32	30	26
YAM3	−0.17	0.11	−0.11	−0.15	0.07	−0.15	10	15	33	30	−42	31
YAM1	−0.16	0.08	−0.11	−0.15	0.05	0.16	10	15	34	32	−8	−13
YAM7	−0.15	0.11	0.13	−0.12	0.06	0.14	10	15	−41	29	−42	15
YAM2	−0.16	−0.12	−0.12	−0.13	−0.07	−0.13	10	30	33	29	28	32
YAM9	0.22	−0.08	0.10	−0.14	0.06	−0.12	36	−3	−41	28	−21	−3
YAM10	0.23	−0.16	−0.10	−0.17	−0.11	0.22	35	−30	26	29	−31	−13
YAM6	−0.18	−0.11	−0.09	−0.16	−0.05	−0.15	10	28	27	29	28	26

The cross‐correlogram reveals that, during a time window of 7 years (the longest duration of an ENSO cycle), the peak negative correlation between ONI and temperature is observed with a lag of approximately 10–15 months, whereas the peak positive correlation occurs with a lag between 35–40 months (Table 4). When the negative correlation peak between ONI and temperature is observed, correlations between ONI and the other hydroclimatic parameters shift from negative to positive, except for the BAIE watershed, where negative correlation persists. Conversely, when the positive correlation peak between ONI and temperature is reached, the correlation between ONI and the other parameters attains a negative peak.

## 4 Discussion

### 4.1 Integrated Hydroclimatic Dynamics

**FIGURE 10:**
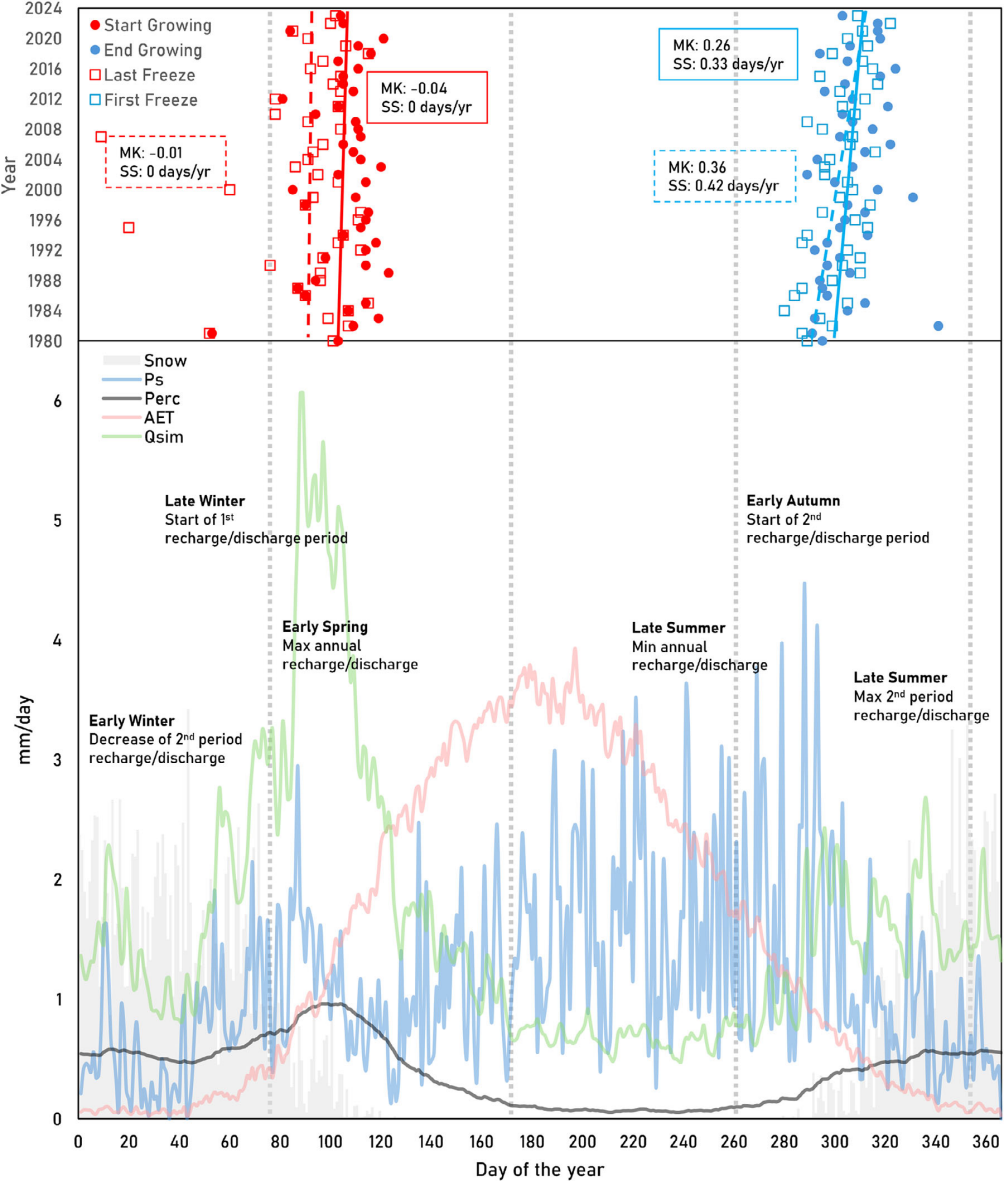
Yearly hydro‐meteorological cycle for YAM9. Curves show long‐term mean daily values (1994‐2023) of snow, effective precipitation (Ps), percolation (Perc), actual evapotranspiration (AET), and simulated discharge (Qsim) from GR4J–CemaNeige (mm day^−1^). Annual growing‐season dates (circles) and freeze dates (squares) are shown in the top: start of growing season and last freeze in red; end of growing season and first freeze in blue. Trend lines for growing‐season dates are straight; trend lines for the freeze dates are dashed. Sen's slope (SS) and Mann–Kendall τ (MK τ) summarize trends. Grey dotted lines mark astronomical seasons.

The observed trends across Montérégie reveal complex and seasonally variable interactions between climate and water‐cycle dynamics, controlled by regional large‐scale processes, watershed characteristics, and local site conditions. Temperature anomalies show a clear increasing trend (Figure 3, top‐left), with the strongest warming in summer‐autumn and the greatest variability during winter (Figure 5, top‐left), consistent with long‐term warming trends observed in southeastern Canada and other cold regions [101, 102]. This warming impact other hydrological processes, causing earlier snowmelt and extending the non‐freezing and growing seasons (Figure 10, top; Tables S2.3 and S2.4), which in turn affect the timing of streamflow discharge and groundwater recharge.

**FIGURE 11:**
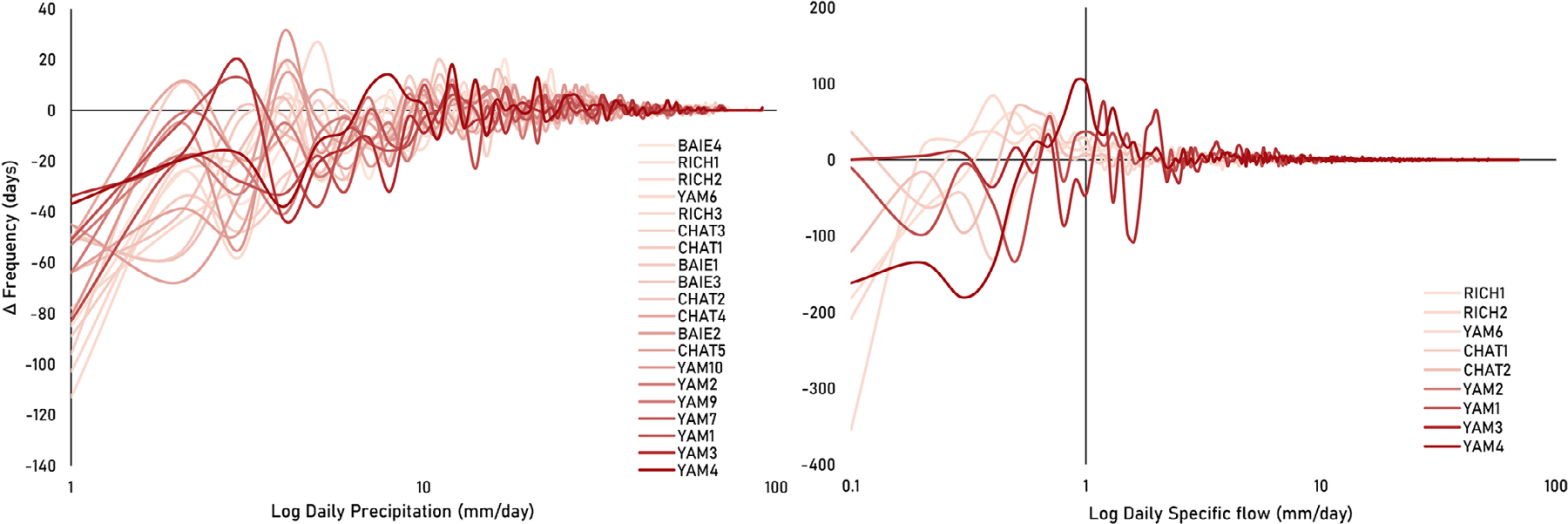
Comparison of daily precipitation (left) and daily specific flow (right) frequency distributions between 1980–1990 and 2010–2020 across Montérégie watersheds. The *x*‐axes show logarithmic daily values (precipitation rounded to integers and specific flow rounded to one decimal), and the y‐axes show changes in frequency (Δ days). Each line represents a watershed, with color intensity indicating elevation (light red = low, dark red = high). Only watersheds with records covering both decades are shown.

Precipitation intensity shows overall increases, especially during spring and summer (Figure 5 top‐right), while moderate‐intensity rainfall events have decreased (Figure 6). These changes are illustrated by the redistribution of daily precipitation frequencies between the 1980–1990 and 2010–2020 decades, with fewer low‐intensity events and an increased occurrence of higher daily precipitation amounts across most watersheds (Figure 11, left). This shift can reduce infiltration in low‐permeability soils and increase runoff, but may enhance recharge in areas with permeable soils or fractured outcrops. However, a decline in low‐intensity rainfall events can limit deep percolation in well‐drained, vegetated soils (e.g., over sandy or fractured substrates) where gentle, prolonged precipitation typically favors infiltration. These areas, often located in forested or agricultural zones with low slopes, may be especially vulnerable to more intense but shorter rainfall episodes, reducing their recharge potential despite stable or rising total precipitation. These changes are also reflected in the streamflow discharge of many watersheds, with rising winter‐spring flows due to earlier snowmelt and reduced frost, while summer‐autumn responses remain more variable, with many upslope watersheds showing decreasing discharge (Figure 7). Similar increases in winter‐spring streamflow have been documented at regional scales in southern Québec and across Canada, reflecting climate‐driven shifts in snowmelt timing and recharge processes [34, 35]. Consistent with this interpretation, daily specific flow distributions show a marked redistribution toward higher flow magnitudes in the recent decade, indicating a shift in hydrological regimes rather than a uniform increase in discharge across all flow conditions (Figure 11, right).

Groundwater level trends also follow a heterogeneous pattern, influenced by both surface water inputs and subsurface properties. Some areas show consistent increases in winter‐spring levels (e.g., central RICH and mid‐YAM), while others, particularly eastern CHAT and southern RICH, show declines during the same periods (Figure 5, bottom‐right). The combined interpretation of streamflow and piezometric trends suggests that climate‐induced changes are mediated by local hydrogeological conditions, including aquifer confinement, soil texture, and land use. This integration confirms that warming and changing precipitation regimes in Montérégie are already shifting water partitioning between surface and subsurface reservoirs, with contrasting outcomes depending on geomorphology and land cover.

### 4.2 Spatial and Temporal Variability in Recharge Indicators

The spatial and temporal variability of groundwater recharge in Montérégie is influenced by climatic, topographic, and land cover differences between watersheds (Figure 7). While GR4J simulated percolation and effective precipitation (Ps) provide indicators of potential recharge, their spatial patterns and trends highlight contrasting dynamics between surface and subsurface systems.

Recharge timing shows a strong seasonal contrast. Simulations indicate that winter‐spring percolation accounts for ∼70% of annual totals in most watersheds, related to snowmelt inputs and low evapotranspiration, whereas summer‐autumn percolation contributes only ∼12%, limited by an increasing evapotranspiration demand during longer growing seasons and higher temperatures (Table 3). This suggests a seasonal shift in recharge windows, consistent with broader observed hydroclimatic changes (Section 4.1).

Simulated recharge patterns generally follow regional climate signals, like the increasing percolation in BAIE, that reflects rising winter precipitation and earlier melt. However, inconsistencies arise when simulated percolation is compared against observed groundwater level trends, particularly in regions like CHAT and YAM. Such mismatches highlight the influence of local factors, like geological heterogeneity, pumping, and land cover conditions, which are not fully represented by the lumped GR4J model.

Streamflow percentiles show contrasting behaviors. Low‑flow conditions (Q5), usually related to baseflow and potential recharge, are increasing in many watersheds, while high‑flow percentiles (Q95) are also rising in some of them (Tables S2.7 and S2.8), likely reflecting more intense rainfall or rapid snowmelt. Similar interpretations have been reported in southern Québec and other cold, humid regions of Canada, where rising streamflow and baseflow indicators were related to increasing groundwater recharge under changing climatic conditions [34, 35, 103]. However, in this study, these increases do not consistently match with rising simulated percolation or groundwater level trends, which are declining in several regions. This indicates that higher streamflow may result from greater surface runoff rather than enhanced recharge. This highlights the need for caution when using streamflow metrics as proxies for recharge without corroborating evidence from groundwater levels or model outputs.

Comparisons between early (1980–1989) and recent (2013–2022) decades show increases in GR4J reservoir parameters across most watersheds, except for percolation, which has decreased in several regions (Figure 9). This pattern supports the idea that climate change is shifting the timing of recharge rather than uniformly boosting its magnitude. Overall, these results demonstrate that recharge in Montérégie reflects the combined influence of climate variability, land cover patterns, and subsurface properties, highlighting the need for integrated approaches that combine trend analysis, modeling, and groundwater observations.

### 4.3 Implications for Groundwater Sustainability

The pronounced spatial variability of groundwater levels across Montérégie, with both increasing and decreasing trends (Figure 7), reflects the strong influence of local hydrogeological conditions on recharge behavior, such as aquifer confinement, sediment texture, and land cover. Increases in winter‐spring groundwater levels in areas like central RICH and mid‑YAM suggest favorable recharge related to snowmelt and low evapotranspiration rates, whereas declines in eastern CHAT and southern RICH indicate potential stress on aquifer storage and resilience. The seasonal patterns reinforce this contrast, since, generally, winter‐spring levels tend to increase, while summer–autumn levels tend to decrease or stabilize. This is consistent with earlier snowmelt and extended growing seasons that shift recharge toward colder months while reducing potential infiltration during warmer periods.

Similarly, GR4J simulations also reinforce this seasonality, with most annual percolation occurring during winter‐spring and minimal contributions during summer–autumn (Table 3). However, decreasing simulated percolation in many watersheds contrasts with stable or increasing streamflow and groundwater levels, suggesting that surface runoff or snowmelt dynamics may be enhancing streamflow discharge without corresponding infiltration increases. In this context, increases in low‐flow (Q5) should be interpreted carefully, as they may reflect shallow processes rather than deep aquifer recharge.

These patterns likely reflect lithological contrasts between watersheds (Figure 2). For instance, areas covered by clayey marine sediments (e.g., eastern CHAT and southern RICH) may have low permeability and limited vertical flow, resulting in reduced recharge and shallow storage capacity. In contrast, coarser glaciofluvial deposits (e.g., central RICH and YAM) can support deeper infiltration and more sustained groundwater levels. Furthermore, where till layers dominate, percolation may be delayed or redirected laterally, affecting both recharge timing and surface‐groundwater connectivity. The GR4J‐calibrated parameters reflect these differences through variations in production and routing stores, as well as groundwater exchange coefficients (Figure 9). However, due to the lumped nature of the model, detailed lithological controls on recharge cannot be explicitly resolved, highlighting the value of coupling modeling with site‐specific geological data. Negative values from the GR4J‑calibrated groundwater exchange coefficients in BAIE suggest infiltration from stream to aquifers, while positive coefficients indicate groundwater discharge to rivers. These patterns are consistent with observed groundwater level trends and reinforce the importance of local hydrogeology in shaping recharge dynamics.

All together, piezometric observations, model outputs, and trend analyses provide a clearer understanding of groundwater sustainability in Montérégie. Recharge responses to climate change vary widely and cannot be inferred from climate forcing alone. Aquifer resilience depends on local geomorphology, land cover, and seasonality. Areas with declining groundwater levels, such as eastern CHAT and southern RICH, require closer monitoring and management, whereas regions with stable or rising levels may offer opportunities for recharge protection. In all cases, understanding the decoupling between surface indicators (streamflow, runoff) and subsurface behavior is essential for sustainable water governance under evolving climate regimes.

### 4.4 Regional Climate Signals and Global Drivers

Correlating regional hydroclimatic anomalies with the Oceanic Niño Index (ONI) reveals weak to moderate relationships (r = −0.23 to 0.23), indicating that ENSO plays a secondary role compared with regional and watershed‑scale conditions (Table 4). Temperature and streamflow anomalies show the strongest correlation with ONI, with lag times of 10–40 months, highlighting delayed mid‑latitude responses to global ocean‐atmosphere variability. Simulated percolation also correlates moderately with ONI, although patterns vary across watersheds, indicating spatial differences in ENSO sensitivity.

The alternation of negative correlations at short lags (∼10–15 months) and positive correlations at longer lags (∼35–40 months) suggests multi‑stage ENSO influences related to transitions between El Niño and La Niña phases and their delayed impacts on precipitation, snowmelt, and temperature regimes in southern Quebec. However, the low correlation strengths indicate that ENSO alone cannot account for the region's diverse recharge patterns. Watersheds with pronounced recharge signals (either in percolation or groundwater levels) do not necessarily coincide with high ONI correlations, implying that local geomorphology, land cover, and human activities modulate or dampen ENSO effects. Therefore, while ENSO contributes to regional climate variability, Montérégie's hydroclimatic behavior appears to be primarily influenced by regional atmospheric processes and watershed characteristics, limiting the predictive value of ENSO‑based forecasts unless combined with regional analyses.

### 4.5 Methodological Insights and Limitations

This study offers a detailed hydroclimatic evaluation of recharge dynamics in Montérégie, but several methodological considerations are essential for interpreting the results. MICE imputation was necessary to reconstruct long climate, streamflow, and piezometric records, and the iterative procedure improved internal consistency. Still, imputation introduces bias, especially in older or sparsely piezometric records. Missing data may coincide with extreme events such as droughts or floods, which are poorly represented in imputed series and may dampen or distort long‑term trend detection. This highlights the need for caution when interpreting groundwater‑level trends in data‑limited contexts.

Recharge estimates from GR4J+CemaNeige simulations provide useful watershed‑scale insights but remain proxies for potential recharge, as the lumped model cannot represent aquifer properties, vadose‑zone processes, or human influences such as pumping. Consequently, simulated percolation should be viewed as an indicator of potential recharge rather than a direct measure of infiltration into aquifers, an important distinction in landscapes where local conditions strongly modulate recharge. Mismatches between simulated percolation and observed hydrological responses highlight these constraints, since declining percolation alongside stable or rising streamflow and groundwater levels suggest that increased runoff or snowmelt may elevate flows without enhancing infiltration. Similarly, calibration‐derived exchange parameters do not always align with piezometric trends.

Model calibration was evaluated using the standard NSE, which emphasizes overall flow dynamics. While this metric is appropriate for regional comparisons, it is less sensitive to low‑flow conditions that are more directly related to baseflow and groundwater contributions. The use of alternative criteria, such as the logarithmic NSE (NSElog), which emphasizes low‑flow behavior, could provide additional insight into the representation of recharge‐related processes. Incorporating NSElog is therefore identified as a valuable extension for future work aimed at refining the evaluation of baseflow and percolation dynamics.

While the GR4J+CemaNeige model allows efficient simulation and calibration across multiple watersheds, it does not explicitly simulate 3D groundwater flow or subsurface heterogeneity. This limits its ability to capture recharge‐discharge interactions and vertical flow mechanisms. In contrast, Delottier et al. (2022) applied a fully coupled surface‐subsurface model (HydroGeoSphere) covering our study area, integrating both piezometric and streamflow data to simulate baseflow with an Iterative Ensemble Smoother [104]. Their results emphasize the importance of subsurface structure and uncertainty in parameter estimation for local groundwater‐surface water interactions, as supported by other regional studies showing that local flow systems can dominate groundwater circulation and streaflow exchanges [51, 52]. While our model emphasizes regional trend detection and inter‐watershed comparison, physically based approaches like this one offer complementary insights into the mechanisms governing recharge. Future work could explore coupling both strategies to improve predictive power at both local and regional scales.

Trend analyses (Mann–Kendall and Sen's Slope) add value, but also carry assumptions, such as independence and homogeneity, that may not fully apply to hydrological time series influenced by persistence, seasonality, or land cover changes. Their sensitivity to record length and data quality further complicates interpretation. Similarly, ONI correlations provide insight into global teleconnections, but their modest strengths and variable lag times reinforce the need to contextualize large‑scale climate signals within regional and local conditions. In general, results must be viewed within the limits of the data, model structure, and analytical tools. Integrating multiple evidence sources strengthens interpretation, but uncertainties remain, underscoring the need for hybrid, multi‑scale approaches in future work.

## 5 Conclusions

The sequential implementation of MICE significantly enhances the data integrity of climate, streamflow, and groundwater level records, reducing bias in historical analysis and trend detections. Our findings highlight a clear warming trend, with the strongest temperature increases in summer and the greatest variation during winter. This results in earlier snowmelt, shifting the timing of river discharge and infiltration, and causing shifts in groundwater recharge patterns. Precipitation intensities are rising, which may increase flood and runoff hazards in low‐permeability areas, while promoting infiltration in regions underlain by fractured or coarse‐grained deposits (e.g., glaciofluvial sand and gravel).

Streamflow data reveal increasing anomalies in winter and spring, indicating earlier snowmelt and the possibility of higher spring floods, related to the high temperature variability during winter. Declining or mixed streamflow trends during summer‐autumn seasons are potential constraints related to water availability and ecosystem health, specifically in upstream regions. The CHAT watershed is one of the regions showing alert signs, since its upslope gauges show decreases in seasonal streamflow anomalies and extreme values (Q95 and Q5). High seasonal contrasts observed at the RICH3 gauge (increased spring flow followed by summer declines) likely reflect the watershed's steep terrain and limited vegetation cover near the Mont Rougemont escarpment, which may amplify runoff responses. However, additional analyses would be needed to isolate the specific influence of watershed characteristics on streamflow variability.

Groundwater levels show high regional heterogeneity, suggesting a strong influence on local conditions. In some regions, there are rising winter‐spring levels, while others show declines, like northeastern CHAT, where groundwater level trends suggest closer scrutiny to protect aquifer resilience. Whereas central Montérégie shows potential increases that could benefit water availability.

Hydrological simulations realized with GR4J provided additional insights into water dynamics, revealing notable increases in most of the model reservoirs across watersheds, with a few exceptions (Figure 9). However, simulated percolation showed declining values in most watersheds, highlighting regional concerns for long‐term aquifer sustainability. These simulated fluxes are consistent with trends imputed from observed data, reinforcing the spatial variability of hydrological responses in Montérégie, likely driven by differences in climate and land cover conditions.

By combining long‑term observations with regional‐scale hydrological modeling, this study provides a comprehensive assessment of how ongoing hydroclimatic changes are affecting groundwater systems and offers a transferable framework for other cold‑temperate regions. The results provide essential insights for improving water resource management, identifying regions where monitoring should be enhanced, as well as highlighting regions most vulnerable to groundwater recharge changes. They also provide information for flood planning, reservoir management, and land cover conservation, emphasizing the need for strategic land management to support groundwater recharge under of changing climate conditions.

## Author Contributions

**Jorge Mona**: conceptualization, investigation, writing – original draft, writing – review and editing, methodology, validation, formal analysis, data curation. **Christin Müller**: conceptualization, writing – review and editing, supervision, project administration, validation. **Florent Barbecot**: conceptualization, writing – review and editing, methodology, supervision, project administration, resources, funding acquisition. **Janie Masse‐Dufresne**: project administration, research supervision. **Alexandra Mattei**: research supervision.

## Funding

This work has been funded by the MAMH (Ministère des Affaires Municipales et de l'Habitation) and the Natural Sciences and Engineering Research Council of Canada (NSERC) (discovery grant RGPIN‐2020‐ 05552).

## Conflicts of Interest

The authors declare no conflicts of interest.

## Supporting information




**Supporting File**: gch270098‐sup‐0001‐SuppMat.docx.

## Data Availability

The hydroclimatic and land cover datasets used in this study are publicly available from the references provided in the manuscript. Processed datasets and model outputs produced during the current study are available from the corresponding author on reasonable request.
