# Supplementary material for: Observed Hydroclimatic Trends and Their Implications Over Water‐Cycle Dynamics in Montérégie, Southern Quebec, Canada
Source: Glob Chall. 2026 Apr 6;10(4):e00564. doi: 10.1002/gch2.202500564 (PMC13053673; doi:10.1002/gch2.202500564)
Supplement: Supplementary file 1 — Supporting File: gch270098‐sup‐0001‐SuppMat.docx. [file GCH2-10-e00564-s001.docx]

SUPPORTING INFORMATION

*Jorge Mona*, Christin Müller, Jane-Masse Dufresne, Alexandra Mattei, and Florent Barbecot*

S1. Imputation Uncertainties

This section shows the pooling (combination) of the univariate estimates from the five imputations data set, according to Rubin's rules (Rubin 1987), obtained from the coefficients of a linear mixed model that predicts the imputed values from the predictor variables (Tmax, Tmin, Prcp, and other meteorological/hydrometric/piezometric stations).

The tables show the pooled estimate, the between, within and total variance, the relative increase in variance, the degrees of freedom for the t reference distribution and the fraction of missing information:

- 1st record: date of the first data record of each station (beginning of the imputation).
- missing: percentage of missing data during the imputation period (first record - 2023).
- estimate: pooled (combined) estimate of the model’s intercept, after fitting the model separately on each imputed dataset and combining the results.
- ubar: for each imputed dataset, there is an estimate of the variance for the intercept. ubar is the average of these variance estimates across all imputations.
- b: measures how much the intercept estimates vary across the multiple imputations.
- t: ratio of the pooled estimate of the intercept to its standard error.
- dfcom: degrees of freedom would be if there were no missing data.
- df: adjusted degrees of freedom for your pooled estimate, accounting for the extra uncertainty from imputation.
- riv: Relative Increase in Variance. Indicator of how much extra variance is introduced by having missing data that requires imputation.
- lambda: proportion of total variance that is attributable to the between-imputation variance
- fmi: Fraction of Missing Information. Is an indicator of how much of the variance in the estimate is due to missing data and imputation.

S1.1. Meteorological Stations

For imputation of missing climate records (maximum & minimum temperature, and precipitation) at the meteorological stations, the other climatic parameters of the same station (temperature(s) and/or precipitation), and the climatic parameters of older stations where imputations have already been made, are used as predictors.

S1.1.1. Minimum Temperature

**Table S1.1.** Uncertainty parameters from the minimum temperature intercept obtained after applying pooling to multiple imputations for each meteorological station.^[70]^ From top to bottom, the stations are presented according to their imputation order, from the one with the oldest records to the most recent.

| Stations | 1st record | missing | estimate | ubar | b | t | dfcom | df | riv | lambda | fmi |
| --- | --- | --- | --- | --- | --- | --- | --- | --- | --- | --- | --- |
| CEDR | 9/1/1913 | 2.1% | -8.13 | 7.8E-04 | 2.5E-05 | 8.1E-04 | 40356 | 2789 | 3.8% | 3.6E-02 | 3.7% |
| SORE | 7/1/1914 | 2.3% | -3.10 | 1.6E-03 | 5.0E-06 | 1.6E-03 | 40050 | 35216 | 0.4% | 3.7E-03 | 0.4% |
| FARN | 6/1/1917 | 11.6% | -0.95 | 1.7E-03 | 2.4E-04 | 2.0E-03 | 38981 | 188 | 17.0% | 1.5E-01 | 15.5% |
| MART | 8/1/1940 | 3.8% | -0.40 | 8.6E-04 | 1.1E-05 | 8.8E-04 | 30516 | 11225 | 1.5% | 1.5E-02 | 1.5% |
| GRAN | 6/1/1948 | 11.7% | -1.29 | 7.5E-04 | 2.1E-04 | 1.0E-03 | 27652 | 66 | 32.7% | 2.5E-01 | 26.9% |
| VERC | 5/14/1958 | 2.5% | -0.34 | 1.1E-03 | 1.8E-05 | 1.1E-03 | 24015 | 7193 | 2.0% | 2.0E-02 | 2.0% |
| RIGA | 10/1/1958 | 1.5% | -0.69 | 1.4E-03 | 3.7E-05 | 1.4E-03 | 23872 | 3469 | 3.2% | 3.1E-02 | 3.2% |
| PRAI | 10/1/1959 | 3.7% | 0.14 | 1.0E-03 | 4.3E-05 | 1.1E-03 | 23504 | 1686 | 4.9% | 4.7E-02 | 4.8% |
| HEMM | 7/1/1960 | 1.8% | 0.43 | 1.2E-03 | 4.2E-06 | 1.3E-03 | 23227 | 21143 | 0.4% | 4.0E-03 | 0.4% |
| ANIC | 12/1/1960 | 0.8% | 0.23 | 7.7E-04 | 6.6E-06 | 7.7E-04 | 23071 | 14302 | 1.0% | 1.0E-02 | 1.0% |
| ORMS | 11/1/1962 | 1.3% | 0.22 | 5.0E-04 | 6.4E-06 | 5.1E-04 | 22368 | 9789 | 1.5% | 1.5E-02 | 1.5% |
| HYAC | 10/1/1963 | 8.4% | 0.01 | 7.8E-04 | 1.3E-04 | 9.3E-04 | 22031 | 145 | 19.8% | 1.7E-01 | 17.7% |
| COTE | 7/1/1966 | 1.7% | -0.42 | 7.2E-04 | 2.7E-05 | 7.5E-04 | 21024 | 1992 | 4.4% | 4.3E-02 | 4.3% |
| FLEU | 10/1/1966 | 0.4% | 0.01 | 8.8E-04 | 5.7E-06 | 8.8E-04 | 20929 | 15855 | 0.8% | 7.7E-03 | 0.8% |
| BONS | 1/1/1967 | 1.8% | -1.02 | 1.7E-03 | 1.0E-05 | 1.7E-03 | 20834 | 16244 | 0.7% | 7.3E-03 | 0.7% |
| LACO | 6/1/1973 | 4.7% | 0.01 | 1.2E-03 | 1.2E-04 | 1.4E-03 | 18488 | 356 | 11.7% | 1.0E-01 | 11.0% |
| SUTT | 11/1/1978 | 2.8% | 0.53 | 9.8E-04 | 1.9E-05 | 1.0E-03 | 16506 | 5122 | 2.4% | 2.3E-02 | 2.3% |
| PIER | 11/1/1980 | 1.9% | -0.52 | 1.3E-03 | 1.3E-05 | 1.4E-03 | 15772 | 10435 | 1.1% | 1.1E-02 | 1.1% |
| CHOI | 9/25/2002 | 3.1% | 0.51 | 2.6E-03 | 5.3E-05 | 2.6E-03 | 7771 | 3581 | 2.5% | 2.4E-02 | 2.5% |

S1.1.2. Maximum Temperature

**Table S1.2.** Uncertainty parameters from the maximum temperature intercept obtained after applying pooling to multiple imputations for each meteorological station.^[70]^ From top to bottom, the stations are presented according to their imputation order, from the one with the oldest records to the most recent.

| Stations | 1st record | missing | estimate | ubar | b | t | dfcom | df | riv | lambda | fmi |
| --- | --- | --- | --- | --- | --- | --- | --- | --- | --- | --- | --- |
| CEDR | 9/1/1913 | 2.4% | 9.30 | 5.4E-04 | 2.5E-05 | 5.7E-04 | 40356 | 1396 | 5.5E-02 | 5.3E-02 | 5.4% |
| SORE | 7/1/1914 | 2.2% | 2.69 | 1.2E-03 | 3.0E-05 | 1.3E-03 | 40050 | 4508 | 2.9E-02 | 2.8E-02 | 2.8% |
| FARN | 6/1/1917 | 11.8% | 0.95 | 1.2E-03 | 3.2E-04 | 1.6E-03 | 38981 | 68 | 3.2E-01 | 2.4E-01 | 26.4% |
| MART | 8/1/1940 | 3.9% | 0.47 | 8.3E-04 | 3.2E-05 | 8.6E-04 | 30516 | 1942 | 4.6E-02 | 4.4E-02 | 4.5% |
| GRAN | 6/1/1948 | 11.4% | -0.18 | 7.7E-04 | 4.1E-04 | 1.3E-03 | 27652 | 26 | 6.4E-01 | 3.9E-01 | 43.2% |
| VERC | 5/14/1958 | 2.8% | 0.45 | 7.7E-04 | 1.6E-05 | 7.9E-04 | 24015 | 5106 | 2.5E-02 | 2.5E-02 | 2.5% |
| RIGA | 10/1/1958 | 1.4% | 0.08 | 8.7E-04 | 6.7E-06 | 8.8E-04 | 23872 | 15899 | 9.2E-03 | 9.1E-03 | 0.9% |
| PRAI | 10/1/1959 | 3.2% | 0.65 | 6.6E-04 | 2.4E-05 | 6.9E-04 | 23504 | 2100 | 4.3E-02 | 4.2E-02 | 4.2% |
| HEMM | 7/1/1960 | 1.8% | 0.36 | 7.4E-04 | 5.7E-06 | 7.5E-04 | 23227 | 15502 | 9.3E-03 | 9.2E-03 | 0.9% |
| ANIC | 12/1/1960 | 1.1% | 0.21 | 6.4E-04 | 6.6E-06 | 6.5E-04 | 23071 | 12244 | 1.2E-02 | 1.2E-02 | 1.2% |
| ORMS | 11/1/1962 | 1.2% | -0.49 | 3.5E-04 | 2.2E-05 | 3.7E-04 | 22368 | 765 | 7.6E-02 | 7.1E-02 | 7.3% |
| HYAC | 10/1/1963 | 8.4% | -0.12 | 4.8E-04 | 1.3E-04 | 6.3E-04 | 22031 | 66 | 3.3E-01 | 2.5E-01 | 26.7% |
| COTE | 7/1/1966 | 2.2% | -0.57 | 3.3E-03 | 1.1E-04 | 3.4E-03 | 21024 | 2392 | 4.0E-02 | 3.8E-02 | 3.9% |
| FLEU | 10/1/1966 | 0.4% | -0.31 | 4.6E-04 | 6.5E-06 | 4.7E-04 | 20929 | 8590 | 1.7E-02 | 1.6E-02 | 1.7% |
| BONS | 1/1/1967 | 1.4% | -0.51 | 1.1E-03 | 7.2E-06 | 1.1E-03 | 20834 | 15506 | 8.1E-03 | 8.0E-03 | 0.8% |
| LACO | 6/1/1973 | 4.6% | 0.88 | 9.5E-04 | 1.4E-04 | 1.1E-03 | 18488 | 183 | 1.7E-01 | 1.5E-01 | 15.6% |
| SUTT | 11/1/1978 | 2.9% | 0.47 | 7.2E-04 | 4.4E-05 | 7.7E-04 | 16506 | 796 | 7.4E-02 | 6.9E-02 | 7.1% |
| PIER | 11/1/1980 | 1.8% | -0.03 | 8.0E-04 | 6.2E-05 | 8.7E-04 | 15772 | 527 | 9.4E-02 | 8.6E-02 | 8.9% |
| CHOI | 9/25/2002 | 3.2% | 0.14 | 1.3E-03 | 7.0E-05 | 1.4E-03 | 7771 | 939 | 6.5E-02 | 6.1E-02 | 6.3% |

S1.1.3. Precipitation

**Table S1.3.** Uncertainty parameters from the precipitation intercept obtained after applying pooling to multiple imputations for each meteorological station.^[70]^ From top to bottom, the stations are presented according to their imputation order, from the one with the oldest records to the most recent.

| Stations | 1st record | missing | estimate | ubar | b | t | dfcom | df | riv | lambda | fmi |
| --- | --- | --- | --- | --- | --- | --- | --- | --- | --- | --- | --- |
| CEDR | 9/1/1913 | 2.5% | 3.60 | 4.7E-03 | 3.9E-04 | 5.2E-03 | 40356 | 485 | 9.9E-02 | 9.0E-02 | 9.4% |
| SORE | 7/1/1914 | 0.6% | 1.71 | 5.0E-03 | 4.9E-05 | 5.0E-03 | 40050 | 16733 | 1.2E-02 | 1.2E-02 | 1.2% |
| FARN | 6/1/1917 | 11.3% | 2.07 | 4.7E-03 | 9.5E-04 | 5.8E-03 | 38981 | 105 | 2.4E-01 | 2.0E-01 | 21.0% |
| MART | 8/1/1940 | 3.6% | 0.40 | 3.3E-03 | 5.3E-05 | 3.4E-03 | 30516 | 8160 | 1.9E-02 | 1.9E-02 | 1.9% |
| GRAN | 6/1/1948 | 1.2% | 0.99 | 4.4E-03 | 2.8E-04 | 4.7E-03 | 27652 | 794 | 7.5E-02 | 7.0E-02 | 7.2% |
| VERC | 5/14/1958 | 2.6% | 0.21 | 4.1E-03 | 1.5E-05 | 4.1E-03 | 24015 | 21463 | 4.4E-03 | 4.4E-03 | 0.4% |
| RIGA | 10/1/1958 | 0.8% | 0.06 | 4.8E-03 | 1.5E-06 | 4.8E-03 | 23872 | 23841 | 3.7E-04 | 3.7E-04 | 0.0% |
| PRAI | 10/1/1959 | 3.0% | -0.05 | 4.5E-03 | 2.4E-04 | 4.7E-03 | 23504 | 1059 | 6.4E-02 | 6.0E-02 | 6.2% |
| HEMM | 7/1/1960 | 1.5% | 0.80 | 4.7E-03 | 1.1E-04 | 4.8E-03 | 23227 | 4637 | 2.7E-02 | 2.6E-02 | 2.7% |
| ANIC | 12/1/1960 | 0.9% | 0.29 | 3.8E-03 | 4.0E-05 | 3.8E-03 | 23071 | 11940 | 1.3E-02 | 1.3E-02 | 1.3% |
| ORMS | 11/1/1962 | 0.0% | -0.13 | 2.7E-03 | 4.0E-06 | 2.7E-03 | 22368 | 21947 | 1.8E-03 | 1.8E-03 | 0.2% |
| HYAC | 10/1/1963 | 8.9% | -0.09 | 4.2E-03 | 1.0E-04 | 4.3E-03 | 22031 | 3871 | 3.0E-02 | 2.9E-02 | 3.0% |
| COTE | 7/1/1966 | 1.9% | 0.10 | 3.5E-03 | 1.5E-04 | 3.7E-03 | 21024 | 1531 | 5.2E-02 | 4.9E-02 | 5.0% |
| FLEU | 10/1/1966 | 0.5% | 0.01 | 4.6E-03 | 2.4E-05 | 4.6E-03 | 20929 | 17424 | 6.1E-03 | 6.1E-03 | 0.6% |
| BONS | 1/1/1967 | 2.1% | 1.30 | 7.8E-03 | 1.6E-04 | 8.0E-03 | 20834 | 5163 | 2.5E-02 | 2.4E-02 | 2.4% |
| LACO | 6/1/1973 | 3.6% | 0.31 | 7.3E-03 | 3.1E-04 | 7.7E-03 | 18488 | 1571 | 5.1E-02 | 4.8E-02 | 4.9% |
| SUTT | 11/1/1978 | 4.8% | 1.30 | 9.6E-03 | 4.7E-04 | 1.0E-02 | 16506 | 1197 | 5.9E-02 | 5.6E-02 | 5.7% |
| PIER | 11/1/1980 | 1.2% | 0.06 | 6.0E-03 | 9.3E-05 | 6.1E-03 | 15772 | 6756 | 1.9E-02 | 1.8E-02 | 1.9% |
| CHOI | 9/25/2002 | 6.0% | -0.41 | 9.4E-03 | 2.2E-04 | 9.7E-03 | 7771 | 3134 | 2.8E-02 | 2.7E-02 | 2.8% |

S1.2. Hydrometric Stations

For imputation of missing streamflow records at the hydrometric stations, the imputed climate data (maximum & minimum temperature, and precipitation), and the imputed streamflow data from older hydrometric stations, are used as predictors. Since the climate data come from two different sources, the streamflow imputation was divided into two periods: data from the climate grids within the boundaries of each sub-watershed were used for a first imputation (1961-2017), while data from the nearest meteorological stations were used for a second imputation (2017-2023).

S1.2.1. Before 2017

**Table S1.4.** Uncertainty parameters from the streamflow intercept obtained after applying pooling to multiple imputations for each hydrometric station before 2017.^[70]^ From top to bottom, the stations are presented according to their imputation order, from the one with the oldest records to the most recent. Stations CHAT5 and YAM10 are not imputed, since they have complete records before 2017*.*

| Stations | 1st record | missing | estimate | ubar | b | t | dfcom | df | riv | lambda | fmi |
| --- | --- | --- | --- | --- | --- | --- | --- | --- | --- | --- | --- |
| YAM1 | 8/20/1965 | 0.83% | 8.1 | 6.1E-02 | 1.2E-03 | 6.2E-02 | 19122 | 5103 | 2.4E-02 | 2.4E-02 | 2.4% |
| YAM2 | 8/20/1965 | 1.42% | -3.4 | 1.2E-01 | 3.5E-03 | 1.2E-01 | 19122 | 3008 | 3.5E-02 | 3.3E-02 | 3.4% |
| YAM3 | 3/27/1968 | 5.97% | 1.0 | 1.8E-03 | 7.9E-05 | 1.9E-03 | 18171 | 1402 | 5.4E-02 | 5.1E-02 | 5.3% |
| YAM4 | 8/16/1968 | 5.51% | -0.2 | 6.0E-03 | 1.6E-04 | 6.2E-03 | 18028 | 3217 | 3.3E-02 | 3.2E-02 | 3.2% |
| YAM6 | 4/10/1969 | 2.65% | -0.2 | 1.8E-02 | 1.2E-03 | 1.9E-02 | 17790 | 736 | 7.8E-02 | 7.2E-02 | 7.5% |
| CHAT1 | 4/14/1970 | 0.14% | 2.7 | 4.4E-01 | 2.7E-02 | 4.7E-01 | 17420 | 821 | 7.3E-02 | 6.8E-02 | 7.0% |
| CHAT2 | 10/1/1973 | 0.93% | -0.9 | 1.4E-02 | 2.5E-03 | 1.7E-02 | 16152 | 118 | 2.2E-01 | 1.8E-01 | 19.7% |
| RICH1 | 10/1/1973 | 0.80% | 0.4 | 1.3E-02 | 6.9E-04 | 1.4E-02 | 16152 | 1101 | 6.2E-02 | 5.8E-02 | 6.0% |
| RICH2 | 8/23/1979 | 2.83% | 0.0 | 1.0E-02 | 2.5E-04 | 1.0E-02 | 13999 | 3596 | 2.9E-02 | 2.9E-02 | 2.9% |
| YAM7 | 11/4/1983 | 0.88% | 0.0 | 2.4E-03 | 4.1E-05 | 2.5E-03 | 12464 | 5536 | 2.0E-02 | 2.0E-02 | 2.0% |
| YAM9 | 10/26/1994 | 6.45% | 1.7 | 1.6E-01 | 1.0E-02 | 1.7E-01 | 8453 | 715 | 7.7E-02 | 7.1E-02 | 7.4% |
| BAIE1 | 7/8/1999 | 5.38% | 0.2 | 1.3E-03 | 7.8E-05 | 1.4E-03 | 6736 | 778 | 7.2E-02 | 6.7E-02 | 6.9% |
| BAIE3 | 10/31/2001 | 0.10% | 0.1 | 1.1E-03 | 1.4E-04 | 1.2E-03 | 5889 | 225 | 1.5E-01 | 1.3E-01 | 13.8% |
| BAIE2 | 11/1/2001 | 3.66% | 0.5 | 2.0E-02 | 7.9E-04 | 2.1E-02 | 5887 | 1406 | 4.8E-02 | 4.6E-02 | 4.8% |
| BAIE4 | 11/2/2001 | 0.61% | 0.0 | 1.3E-04 | 9.9E-06 | 1.4E-04 | 5885 | 532 | 9.0E-02 | 8.2E-02 | 8.6% |
| CHAT3 | 9/22/2004 | 0.27% | 3.2 | 5.4E-02 | 1.9E-03 | 5.6E-02 | 4829 | 1613 | 4.2E-02 | 4.0E-02 | 4.1% |
| CHAT4 | 7/12/2005 | 2.26% | 0.1 | 2.6E-04 | 1.2E-05 | 2.8E-04 | 4534 | 1145 | 5.3E-02 | 5.1E-02 | 5.2% |
| RICH3 | 7/25/2006 | 11.08% | -0.1 | 5.5E-04 | 4.1E-05 | 6.0E-04 | 4156 | 508 | 9.0E-02 | 8.3E-02 | 8.6% |

S1.2.2. After 2017

**Table S1.5.** Uncertainty parameters from the streamflow intercept obtained after applying pooling to multiple imputations for each hydrometric station after 2017.^[70]^ From top to bottom, the stations are presented according to their imputation order, from the one with the oldest records to the most recent. Missing stations have not been imputed, since they have complete records before 2017.

| Stations | 1st record | missing | estimate | ubar | b | t | dfcom | df | riv | lambda | fmi |
| --- | --- | --- | --- | --- | --- | --- | --- | --- | --- | --- | --- |
| BAIE2 | 1/1/2018 | 0.05% | 0.04 | 3.5E-02 | 4.3E-04 | 3.5E-02 | 2167 | 1917 | 1.5E-02 | 1.5E-02 | 1.6% |
| YAM10 | 1/1/2018 | 0.27% | 0.29 | 2.1E-02 | 1.6E-03 | 2.3E-02 | 2167 | 447 | 9.1E-02 | 8.3E-02 | 8.7% |
| YAM6 | 1/1/2018 | 5.93% | 0.67 | 9.2E-02 | 4.3E-04 | 9.3E-02 | 2167 | 2117 | 5.7E-03 | 5.6E-03 | 0.7% |
| YAM1 | 1/1/2018 | 0.96% | 0.72 | 7.9E-02 | 2.9E-03 | 8.3E-02 | 2167 | 1082 | 4.4E-02 | 4.2E-02 | 4.4% |
| YAM3 | 1/1/2018 | 0.18% | 0.59 | 7.1E-03 | 6.6E-05 | 7.1E-03 | 2167 | 2008 | 1.1E-02 | 1.1E-02 | 1.2% |
| BAIE4 | 1/1/2018 | 2.74% | 0.08 | 2.8E-04 | 2.7E-05 | 3.2E-04 | 2167 | 326 | 1.1E-01 | 1.0E-01 | 10.7% |
| RICH3 | 1/1/2018 | 37.70% | 0.06 | 6.9E-04 | 2.6E-04 | 1.0E-03 | 2167 | 40 | 4.6E-01 | 3.1E-01 | 34.6% |

S1.3. Piezometers

For imputation of missing groundwater level records at the piezometric stations, the imputed climate data (maximum & minimum temperature, and precipitation), and the imputed groundwater levels from older piezometric stations, are used as predictors. Since the climate data come from two different sources, the groundwater levels imputation was divided into two periods: data from the climate grids within the boundaries of each main watershed (where the piezometer is located) is used for a first imputation (1961-2017), while data from the nearest meteorological station were used for a second imputation (2017-2023). Due to the high percentage (> 40%) of daily missing values (missing), and the variance of the imputations associated with these missing values (fmi), records prior to 2000, and some piezometers with records between 2000-2023, were discarded for the analysis*.*

S1.3.1. Before 2000

**Table S1.6.** Uncertainty parameters from the groundwater levels intercept obtained after applying pooling to multiple imputations for each piezometer before 2000.^[70]^ From top to bottom, the stations are presented according to their imputation order, from the one with the oldest records to the most recent.

| Piezometer | 1st record | missing | estimate | ubar | b | t | dfcom | df | riv | lambda | fmi |
| --- | --- | --- | --- | --- | --- | --- | --- | --- | --- | --- | --- |
| CHAT25 | 8/23/1974 | 97.8% | 31.5 | 2.1E-04 | 6.3E-03 | 7.8E-03 | 6869 | 4 | 3.5E+01 | 9.7E-01 | 98.0% |
| YAM19 | 7/17/1975 | 98.2% | 64.9 | 9.7E-02 | 1.1E+01 | 1.3E+01 | 6539 | 4 | 1.4E+02 | 9.9E-01 | 99.5% |
| YAM18 | 7/30/1975 | 98.2% | 31.3 | 5.1E-01 | 1.1E+01 | 1.4E+01 | 6525 | 4 | 2.6E+01 | 9.6E-01 | 97.4% |
| YAM20 | 9/11/1975 | 98.2% | 161.8 | 2.0E-01 | 2.8E+01 | 3.4E+01 | 4590 | 4 | 1.7E+02 | 9.9E-01 | 99.6% |
| RICH10 | 4/24/1979 | 97.1% | 39.5 | 1.5E+02 | 6.6E+03 | 8.1E+03 | 5159 | 4 | 5.2E+01 | 9.8E-01 | 98.6% |
| CHAT28 | 11/5/1980 | 97.5% | -56.5 | 1.2E+03 | 2.5E+04 | 3.1E+04 | 3822 | 4 | 2.4E+01 | 9.6E-01 | 97.2% |
| CHAT29 | 1/9/1981 | 51.5% | 36.9 | 8.5E+01 | 7.5E+01 | 1.8E+02 | 3852 | 15 | 1.1E+00 | 5.1E-01 | 56.8% |
| CHAT30 | 9/22/1982 | 97.8% | 29.0 | 5.0E+01 | 2.1E+03 | 2.6E+03 | 3225 | 4 | 5.0E+01 | 9.8E-01 | 98.6% |
| CHAT31 | 9/30/1982 | 97.9% | -3.8 | 8.9E+00 | 8.2E+02 | 9.9E+02 | 3216 | 4 | 1.1E+02 | 9.9E-01 | 99.4% |
| CHAT32 | 6/8/1984 | 6.2% | 101.4 | 1.9E+02 | 7.7E+02 | 1.1E+03 | 3766 | 6 | 4.9E+00 | 8.3E-01 | 87.0% |
| CHAT26 | 9/7/1984 | 98.1% | -151.7 | 9.5E+01 | 2.1E+04 | 2.5E+04 | 2518 | 3 | 2.6E+02 | 1.0E+00 | 99.8% |
| CHAT27 | 9/7/1984 | 98.2% | 54.1 | 1.4E+01 | 1.6E+03 | 1.9E+03 | 2510 | 3 | 1.3E+02 | 9.9E-01 | 99.5% |

S1.3.2. Between 2000-2017

**Table S1.7.** Uncertainty parameters from the groundwater levels intercept obtained after applying pooling to multiple imputations for each piezometer between 2000-2017.^[70]^ From top to bottom, the stations are presented according to their imputation order, from the one with the oldest records to the most recent. Piezometers without missing values are not presented in this table.

| Piezometers | 1st record | missing | estimate | ubar | b | t | dfcom | df | riv | lambda | fmi |
| --- | --- | --- | --- | --- | --- | --- | --- | --- | --- | --- | --- |
| CHAT27 | 4/20/2000 | 0.7% | 30.9 | 2.9E-04 | 5.9E-07 | 2.9E-04 | 6461 | 6381 | 2.5E-03 | 2.5E-03 | 0.3% |
| CHAT28 | 5/16/2000 | 3.3% | 32.3 | 4.8 | 7.8E-01 | 5.7 | 6432 | 143 | 2.0E-01 | 1.7E-01 | 17.7% |
| CHAT30 | 5/16/2000 | 0.4% | 5.9 | 1.6E-01 | 1.9E-03 | 1.7E-01 | 6432 | 4920 | 1.4E-02 | 1.4E-02 | 1.4% |
| CHAT32 | 5/16/2000 | 7.7% | 24.7 | 1.6E-01 | 3.7E-02 | 2.0E-01 | 6432 | 80 | 2.9E-01 | 2.2E-01 | 24.1% |
| CHAT29 | 5/17/2000 | 0.5% | 3.5 | 1.8 | 6.8E-03 | 1.8 | 6430 | 6198 | 4.5E-03 | 4.5E-03 | 0.5% |
| CHAT3 | 6/16/2000 | 61.0% | -31.6 | 6.9E-01 | 3.3E-01 | 1.1 | 6399 | 30 | 0.6 | 3.7E-01 | 40.5% |
| CHAT31 | 7/7/2000 | 0.4% | 11.2 | 8.5E-03 | 2.0E-06 | 8.5E-03 | 6377 | 6372 | 2.8E-04 | 2.8E-04 | 0.1% |
| CHAT24 | 7/3/2001 | 0.2% | 17.0 | 2E-01 | 4.7E-05 | 2.1E-01 | 6015 | 6011 | 2.8E-04 | 2.8E-04 | 0.1% |
| CHAT25 | 10/31/2002 | 0.6% | 15.4 | 3.0E-01 | 1.9E-03 | 3.0E-01 | 5529 | 5070 | 7.8E-03 | 7.7E-03 | 0.8% |
| CHAT26 | 5/14/2003 | 14.3% | 7.4 | 2 | 1.6E-01 | 2.0 | 5333 | 431 | 1.0E-01 | 9.2E-02 | 10% |
| CHAT10 | 3/24/2004 | 4.5% | 7.4 | 4.2E-01 | 1.2E-02 | 4.3E-01 | 5015 | 1990 | 3.6E-02 | 3.4E-02 | 3.5% |
| CHAT5 | 3/24/2004 | 4.8% | -25.3 | 1.6 | 1.3E-01 | 1.7 | 5015 | 438 | 1.0E-01 | 9.1E-02 | 9.5% |
| CHAT9 | 3/24/2004 | 0.0% | 32.2 | 4E-01 | 2.9E-07 | 4.1E-01 | 5015 | 5012 | 8.6E-07 | 8.6E-07 | 0.04% |
| CHAT1 | 3/25/2004 | 3.1% | -6.0 | 3 | 2.2 | 6 | 5005 | 21 | 7.7E-01 | 4.3E-01 | 48.2% |
| CHAT12 | 3/25/2004 | 45.8% | -54.1 | 15 | 11 | 29 | 5005 | 18 | 0.9 | 4.8E-01 | 52.6% |
| CHAT13 | 3/25/2004 | 4.3% | 247.5 | 2 | 0.6 | 2 | 5005 | 44 | 4.3E-01 | 3.0E-01 | 33% |
| CHAT14 | 3/25/2004 | 77.4% | -8.4 | 16 | 83.6 | 116 | 5005 | 5 | 6.4 | 8.6E-01 | 89.7% |
| CHAT16 | 3/25/2004 | 0.2% | 52.3 | 8 | 6.6 | 16 | 5005 | 16 | 9.7E-01 | 4.9E-01 | 54.4% |
| CHAT2 | 3/25/2004 | 45.2% | -0.7 | 2 | 0.9 | 3 | 5005 | 35 | 0.5 | 3.3E-01 | 36.9% |
| CHAT21 | 3/25/2004 | 4.0% | 1.5 | 2 | 8.3E-01 | 3 | 5005 | 34 | 5.2E-01 | 3.4E-01 | 38.0% |
| CHAT22 | 3/25/2004 | 19.4% | 6.1 | 2 | 3.5 | 7 | 5005 | 9 | 1.9 | 6.5E-01 | 71% |
| CHAT8 | 3/25/2004 | 62.3% | -86.7 | 6 | 4.2 | 11 | 5005 | 19 | 0.8 | 4.5E-01 | 50% |
| CHAT6 | 5/5/2004 | 0.6% | -59.7 | 44 | 5.3E-01 | 44 | 4962 | 3919 | 1.4E-02 | 1.4E-02 | 1% |
| CHAT7 | 5/5/2004 | 8.5% | 15.9 | 5 | 0.2 | 6 | 4962 | 1130 | 0.1 | 5.2E-02 | 5.4% |
| CHAT23 | 5/6/2004 | 3.6% | -284.5 | 270 | 5.5 | 277 | 4960 | 2861 | 2.4E-02 | 2.4E-02 | 2.5% |
| YAM19 | 8/24/2006 | 0.0% | -114.0 | 19 | 0.7 | 20 | 4118 | 1462 | 4.3E-02 | 4.2E-02 | 4% |
| YAM20 | 8/24/2006 | 4.6% | 162.5 | 3 | 6.0E-02 | 3 | 4118 | 2369 | 2.7E-02 | 2.6E-02 | 2.7% |
| RICH10 | 9/13/2007 | 93.7% | 43.8 | 41 | 437.7 | 566 | 3732 | 5 | 12.9 | 9.3E-01 | 95% |
| RICH3 | 11/17/2010 | 8.5% | -131.4 | 113 | 5.3 | 120 | 2570 | 896 | 5.6E-02 | 5.3E-02 | 5.5% |
| YAM5 | 11/18/2010 | 2.0% | 126.7 | 12168 | 377.4 | 12621 | 2568 | 1377 | 3.7E-02 | 3.6E-02 | 3.7% |
| RICH1 | 11/21/2010 | 1.4% | -80.5 | 158 | 3.5E-01 | 158 | 2564 | 2544 | 2.6E-03 | 2.6E-03 | 0.3% |
| YAM9 | 11/23/2010 | 0.1% | 167.5 | 73 | 6.0E-02 | 73 | 2561 | 2555 | 9.8E-04 | 9.8E-04 | 0.2% |
| BAIE3 | 11/26/2010 | 0.0% | 46.5 | 123 | 2.1E-03 | 123 | 2557 | 2555 | 2.0E-05 | 2.0E-05 | 0.1% |
| BAIE1 | 12/11/2010 | 1.4% | -119.1 | 82 | 0.5 | 82 | 2540 | 2434 | 7.5E-03 | 7.5E-03 | 0.8% |
| BAIE2 | 12/11/2010 | 0.1% | 329.5 | 1204 | 7.1E-01 | 1205 | 2540 | 2535 | 7.0E-04 | 7.0E-04 | 0.1% |
| YAM8 | 2/1/2011 | 7.5% | 247.4 | 298 | 22.7 | 325 | 2487 | 455 | 9.2E-02 | 8.4E-02 | 8.8% |
| RICH2 | 3/1/2011 | 1.5% | -33.4 | 64 | 2.9E-01 | 64 | 2458 | 2398 | 5.5E-03 | 5.5E-03 | 0.6% |
| BAIE6 | 10/26/2011 | 0.4% | 76.4 | 33 | 1.4E-01 | 33 | 2218 | 2174 | 5.1E-03 | 5.1E-03 | 0.6% |
| YAM11 | 10/28/2011 | 0.0% | -45.3 | 294 | 7.8E-02 | 294 | 2214 | 2211 | 3.2E-04 | 3.2E-04 | 0.1% |
| YAM12 | 10/28/2011 | 0.0% | 0.8 | 32 | 1.8E-02 | 32 | 2214 | 2210 | 6.9E-04 | 6.9E-04 | 0.2% |
| BAIE7 | 6/6/2012 | 9.3% | 29.1 | 10 | 5.3E-01 | 11 | 1991 | 698 | 6.4E-02 | 6.0E-02 | 6% |
| VS2 | 8/6/2013 | 22.0% | 807.1 | 7119 | 3670 | 11523 | 1564 | 27 | 6.2E-01 | 3.8E-01 | 42% |

S1.3.3. After 2017

**Table S1.8.** Uncertainty parameters from the groundwater levels intercept obtained after applying pooling to multiple imputations for each piezometer between 2017-2023.^[70]^ From top to bottom, the stations are presented according to their imputation order, from the one with the oldest records to the most recent. Piezometers without missing values are not presented in this table.

| Piezometers | 1st record | missing | estimate | ubar | b | t | dfcom | df | riv | lambda | fmi |
| --- | --- | --- | --- | --- | --- | --- | --- | --- | --- | --- | --- |
| CHAT16 | 1/1/2018 | 15.84% | 22 | 4.3 | 2.0 | 6.8 | 1173 | 29 | 0.57 | 0.36 | 40.2% |
| YAM2 | 1/1/2018 | 4.78% | -7.4 | 7.3 | 0.5 | 7.8 | 1404 | 471 | 0.08 | 0.07 | 7.7% |
| BAIE5 | 1/1/2018 | 9.75% | 49.3 | 5.6 | 1.2 | 7.1 | 1929 | 89 | 0.26 | 0.21 | 22.3% |
| CHAT19 | 1/1/2018 | 9.59% | 63.4 | 126.6 | 75.4 | 217.1 | 1929 | 23 | 0.72 | 0.42 | 46.3% |
| CHAT11 | 1/1/2018 | 9.64% | -40.0 | 7.3 | 5.2 | 13.5 | 1930 | 18 | 0.86 | 0.46 | 51.1% |
| CHAT23 | 1/1/2018 | 12.67% | -131.6 | 170 | 91.0 | 279.1 | 1930 | 26 | 0.64 | 0.39 | 43.4% |
| CHAT26 | 1/1/2018 | 8.15% | 9.7 | 3.1 | 1.7 | 5.2 | 1930 | 25 | 0.65 | 0.39 | 43.7% |
| YAM17 | 1/1/2018 | 7.87% | 11.4 | 0.8 | 0.7 | 1.6 | 2103 | 14 | 1.15 | 0.53 | 59.0% |
| YAM5 | 1/1/2018 | 0.61% | 456.5 | 883.7 | 638.2 | 1649.6 | 2103 | 18 | 0.87 | 0.46 | 51.5% |
| YAM8 | 1/1/2018 | 9.33% | 76.5 | 12.8 | 11.1 | 26.2 | 2103 | 15 | 1.04 | 0.51 | 56.3% |
| RICH1 | 1/1/2018 | 8.87% | -86.2 | 19.3 | 19.1 | 42.2 | 2110 | 13 | 1.19 | 0.54 | 59.8% |
| RICH2 | 1/1/2018 | 7.09% | -22.2 | 4.7 | 3.3 | 8.6 | 2110 | 19 | 0.84 | 0.46 | 50.5% |
| RICH7 | 1/1/2018 | 0.05% | 5.7 | 1.8 | 0.4 | 2.3 | 2110 | 83 | 0.27 | 0.21 | 23.2% |
| YAM1 | 1/1/2018 | 7.84% | 5.5 | 0.4 | 0.4 | 0.9 | 2110 | 16 | 1.01 | 0.50 | 55.7% |
| YAM12 | 1/1/2018 | 9.15% | -1.6 | 2.4 | 0.6 | 3.1 | 2110 | 81 | 0.28 | 0.22 | 23.5% |
| CHAT25 | 1/1/2018 | 15.21% | -15.1 | 5.8 | 2.1 | 8.4 | 2123 | 42 | 0.44 | 0.31 | 33.7% |
| RICH4 | 1/1/2018 | 5.32% | -67.8 | 11.4 | 2.6 | 14.5 | 2123 | 84 | 0.27 | 0.21 | 23.1% |

**S2. Trends Magnitudes (Sen’s-Slope & Mann-Kendall)**

**S2.1. Temperature**

S2.1.1 Mann-Kendall Tau (τ) values

**Table S2.1.** Mann-Kendall tau (MK(τ)) values of historical and seasonal temperature anomalies (maximum, minimum and mean) between 1980-2023 for the sub-watersheds of Montérégie. Positive trends are shown in blue-gradient colors, and negative trends are shown in red-gradient colors. Significant trends (p-value <= 0.05) are shown in bold.

| Watershed | Tmin | | | | | Tmax | | | | | Tavg | | | | |
| --- | --- | --- | --- | --- | --- | --- | --- | --- | --- | --- | --- | --- | --- | --- | --- |
|  | Overall | Winter | Spring | Summer | Autumn | Overall | Winter | Spring | Summer | Autumn | Overall | Winter | Spring | Summer | Autumn |
| BAIE2 | **0.18** | **0.32** | 0.10 | **0.41** | **0.43** | **0.11** | 0.17 | 0.04 | 0.05 | **0.34** | **0.15** | **0.25** | 0.06 | **0.21** | **0.39** |
| BAIE3 | **0.14** | **0.27** | 0.04 | **0.28** | **0.37** | **0.08** | 0.15 | -0.01 | -0.04 | **0.31** | **0.11** | **0.24** | 0.01 | 0.12 | **0.36** |
| BAIE1 | **0.16** | **0.29** | 0.06 | **0.32** | **0.39** | **0.11** | 0.20 | 0.07 | 0.04 | **0.36** | **0.14** | **0.25** | 0.05 | 0.15 | **0.38** |
| BAIE4 | **0.16** | **0.29** | 0.07 | **0.29** | **0.37** | **0.11** | **0.22** | 0.08 | 0.00 | **0.35** | **0.14** | **0.24** | 0.07 | 0.15 | **0.38** |
| CHAT5 | **0.14** | **0.24** | 0.10 | **0.25** | **0.41** | **0.18** | 0.19 | 0.18 | **0.23** | **0.42** | **0.17** | **0.23** | 0.15 | **0.25** | **0.42** |
| CHAT4 | **0.14** | **0.22** | 0.12 | **0.29** | **0.37** | **0.13** | 0.13 | 0.10 | 0.16 | **0.38** | **0.14** | 0.19 | 0.10 | **0.23** | **0.38** |
| CHAT2 | **0.13** | **0.24** | 0.07 | **0.21** | **0.38** | **0.16** | 0.18 | 0.15 | **0.21** | **0.41** | **0.15** | **0.22** | 0.12 | **0.24** | **0.41** |
| CHAT1 | **0.23** | **0.28** | **0.23** | **0.40** | **0.50** | **0.17** | 0.16 | 0.16 | **0.26** | **0.42** | **0.21** | **0.24** | 0.19 | **0.34** | **0.49** |
| CHAT3 | **0.23** | **0.28** | **0.21** | **0.38** | **0.48** | **0.20** | 0.21 | 0.19 | **0.28** | **0.45** | **0.22** | **0.26** | 0.19 | **0.33** | **0.49** |
| RICH3 | **0.20** | **0.28** | 0.18 | **0.49** | **0.44** | **0.16** | 0.19 | 0.11 | 0.20 | **0.41** | **0.18** | **0.24** | 0.15 | **0.36** | **0.45** |
| RICH2 | **0.16** | **0.21** | 0.12 | **0.40** | **0.36** | **0.16** | 0.16 | 0.15 | **0.27** | **0.41** | **0.17** | 0.19 | 0.14 | **0.35** | **0.42** |
| RICH1 | **0.21** | **0.28** | 0.19 | **0.51** | **0.45** | **0.16** | 0.20 | 0.12 | **0.21** | **0.41** | **0.19** | **0.24** | 0.16 | **0.36** | **0.45** |
| YAM4 | **0.25** | **0.34** | **0.26** | **0.55** | **0.52** | **0.13** | 0.18 | 0.08 | 0.16 | **0.36** | **0.20** | **0.27** | 0.16 | **0.41** | **0.46** |
| YAM3 | **0.33** | **0.37** | **0.32** | **0.61** | **0.64** | **0.20** | **0.23** | 0.12 | **0.33** | **0.44** | **0.27** | **0.32** | 0.25 | **0.57** | **0.59** |
| YAM1 | **0.29** | **0.35** | **0.30** | **0.63** | **0.58** | **0.19** | 0.17 | 0.15 | **0.33** | **0.43** | **0.25** | **0.28** | 0.22 | **0.52** | **0.54** |
| YAM7 | **0.31** | **0.37** | **0.28** | **0.62** | **0.61** | **0.18** | 0.20 | 0.12 | **0.30** | **0.41** | **0.25** | **0.31** | 0.22 | **0.55** | **0.54** |
| YAM2 | **0.26** | **0.34** | **0.20** | **0.57** | **0.59** | **0.18** | **0.24** | 0.11 | **0.28** | **0.43** | **0.23** | **0.30** | 0.15 | **0.51** | **0.54** |
| YAM9 | **0.26** | **0.32** | **0.25** | **0.60** | **0.55** | **0.18** | 0.18 | 0.15 | **0.32** | **0.43** | **0.23** | **0.27** | 0.19 | **0.51** | **0.52** |
| YAM10 | **0.17** | **0.30** | 0.05 | **0.33** | **0.44** | **0.13** | 0.19 | 0.06 | 0.19 | **0.33** | **0.16** | **0.27** | 0.05 | **0.31** | **0.43** |
| YAM6 | **0.20** | **0.29** | 0.13 | **0.52** | **0.42** | **0.17** | 0.20 | 0.10 | **0.27** | **0.46** | **0.19** | **0.25** | 0.13 | **0.43** | **0.47** |

S2.1.2 Sen’s-Slope values

**Table S2.2.** Sen’s-Slope (SS) values of historical (°C month^-1^) and seasonal (°C season^-1^ year^-1^) temperature anomalies (maximum, minimum and mean) between 1980-2023 for the sub-watersheds of Montérégie. Positive trends are shown in blue-gradient colors, and negative trends are shown in red-gradient colors. Significant trends (p-value <= 0.05) are shown in bold.

| Watershed | Tmin | | | | | Tmax | | | | | Tavg | | | | |
| --- | --- | --- | --- | --- | --- | --- | --- | --- | --- | --- | --- | --- | --- | --- | --- |
|  | Overall | Winter | Spring | Summer | Autumn | Overall | Winter | Spring | Summer | Autumn | Overall | Winter | Spring | Summer | Autumn |
| BAIE2 | **0.003** | **0.073** | 0.016 | **0.033** | **0.051** | **0.002** | 0.037 | 0.006 | 0.005 | **0.043** | **0.003** | **0.053** | 0.011 | **0.017** | **0.051** |
| BAIE3 | **0.002** | **0.063** | 0.007 | **0.025** | **0.043** | **0.001** | 0.027 | -0.001 | -0.003 | **0.038** | **0.002** | **0.042** | 0.001 | 0.009 | **0.044** |
| BAIE1 | **0.003** | **0.071** | 0.011 | **0.024** | **0.046** | **0.002** | 0.042 | 0.009 | 0.002 | **0.044** | **0.003** | **0.054** | 0.009 | 0.012 | **0.049** |
| BAIE4 | **0.003** | **0.070** | 0.013 | **0.026** | **0.047** | **0.002** | 0.044 | 0.010 | 0.001 | **0.046** | **0.003** | **0.056** | 0.010 | 0.012 | **0.049** |
| CHAT5 | **0.002** | **0.067** | 0.014 | **0.018** | **0.044** | **0.004** | 0.046 | 0.028 | **0.032** | **0.057** | **0.003** | **0.054** | 0.022 | **0.026** | **0.050** |
| CHAT4 | **0.003** | **0.060** | 0.016 | **0.025** | **0.041** | **0.003** | 0.027 | 0.017 | 0.019 | **0.051** | **0.003** | **0.042** | 0.015 | **0.023** | **0.047** |
| CHAT2 | **0.002** | **0.062** | 0.009 | **0.015** | **0.040** | **0.003** | 0.040 | 0.025 | **0.027** | **0.054** | **0.003** | **0.049** | 0.018 | **0.021** | **0.048** |
| CHAT1 | **0.004** | **0.084** | **0.037** | **0.042** | **0.060** | **0.003** | 0.039 | 0.025 | **0.034** | **0.058** | **0.004** | **0.057** | 0.029 | **0.039** | **0.059** |
| CHAT3 | **0.004** | **0.084** | **0.036** | **0.043** | **0.060** | **0.004** | 0.048 | 0.032 | **0.035** | **0.064** | **0.004** | **0.067** | 0.030 | **0.040** | **0.061** |
| RICH3 | **0.004** | **0.069** | 0.029 | **0.038** | **0.058** | **0.003** | 0.039 | 0.021 | 0.023 | **0.060** | **0.003** | **0.050** | 0.022 | **0.030** | **0.060** |
| RICH2 | **0.003** | **0.047** | 0.016 | **0.030** | **0.044** | **0.003** | 0.028 | 0.025 | **0.034** | **0.058** | **0.003** | **0.035** | 0.020 | **0.031** | **0.052** |
| RICH1 | **0.004** | **0.069** | 0.029 | **0.041** | **0.058** | **0.003** | 0.040 | 0.020 | **0.024** | **0.059** | **0.003** | **0.052** | 0.022 | **0.031** | **0.061** |
| YAM4 | **0.005** | **0.075** | **0.031** | **0.051** | **0.069** | **0.003** | 0.043 | 0.011 | 0.014 | **0.051** | **0.004** | **0.062** | 0.023 | **0.033** | **0.060** |
| YAM3 | **0.007** | **0.101** | **0.049** | **0.079** | **0.100** | **0.004** | **0.052** | 0.021 | **0.039** | **0.062** | **0.005** | **0.074** | **0.034** | **0.058** | **0.081** |
| YAM1 | **0.005** | **0.080** | **0.044** | **0.063** | **0.080** | **0.004** | 0.036 | 0.026 | **0.041** | **0.061** | **0.005** | **0.060** | **0.034** | **0.050** | **0.070** |
| YAM7 | **0.006** | **0.094** | **0.045** | **0.069** | **0.085** | **0.003** | 0.037 | 0.019 | **0.035** | **0.057** | **0.005** | **0.067** | **0.031** | **0.050** | **0.073** |
| YAM2 | **0.005** | **0.083** | **0.030** | **0.057** | **0.080** | **0.004** | **0.053** | 0.018 | **0.031** | **0.063** | **0.004** | **0.064** | 0.022 | **0.044** | **0.071** |
| YAM9 | **0.005** | **0.075** | **0.038** | **0.057** | **0.073** | **0.004** | 0.036 | 0.023 | **0.035** | **0.062** | **0.004** | **0.056** | 0.031 | **0.045** | **0.069** |
| YAM10 | **0.003** | **0.073** | 0.009 | **0.032** | **0.061** | **0.003** | 0.036 | 0.009 | 0.018 | **0.044** | **0.003** | **0.053** | 0.009 | **0.024** | **0.055** |
| YAM6 | **0.004** | **0.063** | 0.022 | **0.041** | **0.059** | **0.003** | 0.037 | 0.017 | **0.033** | **0.066** | **0.003** | **0.052** | 0.018 | **0.036** | **0.064** |

**S2.2. PET, Non-freezing and Growing Seasons**

S2.2.1 Mann-Kendall Tau (τ) values

**Table S2.3.** Mann-Kendall tau (MK(τ)) values of historical and seasonal PET anomalies, as well as the beginning (Last (Freeze)/Start), end (First (Freeze)/End) and number of days (Days) of the Non-Freezing & Growing seasons, between 1980-2023 for the sub-watersheds of Montérégie. Positive trends are shown in blue-gradient colors, and negative trends are shown in red-gradient colors. Significant trends (p-value <= 0.05) are shown in bold.

| Watershed | PET | | | | | Non-Freezing Season | | | Growing Season | | |
| --- | --- | --- | --- | --- | --- | --- | --- | --- | --- | --- | --- |
|  | Overall | Winter | Spring | Summer | Autumn | Last | First | Days | Start | End | Days |
| BAIE2 | **0.12** | 0.13 | 0.05 | **0.21** | **0.42** | 0.03 | **0.26** | 0.09 | 0.05 | 0.19 | 0.01 |
| BAIE3 | **0.09** | 0.10 | 0.00 | 0.10 | **0.36** | 0.07 | **0.27** | 0.09 | 0.04 | 0.16 | 0.03 |
| BAIE1 | **0.12** | 0.16 | 0.04 | 0.14 | **0.42** | 0.07 | **0.26** | 0.07 | 0.04 | 0.20 | 0.02 |
| BAIE4 | **0.12** | 0.17 | 0.05 | 0.14 | **0.41** | 0.05 | **0.24** | 0.07 | -0.01 | 0.17 | 0.04 |
| CHAT5 | **0.15** | 0.13 | 0.13 | **0.26** | **0.49** | 0.00 | **0.28** | 0.14 | -0.08 | 0.14 | 0.08 |
| CHAT4 | **0.12** | 0.08 | 0.08 | **0.22** | **0.44** | 0.10 | **0.25** | 0.03 | -0.07 | 0.09 | 0.06 |
| CHAT2 | **0.13** | 0.12 | 0.10 | **0.24** | **0.46** | 0.09 | **0.23** | 0.03 | -0.07 | 0.11 | 0.06 |
| CHAT1 | **0.19** | 0.12 | 0.20 | **0.33** | **0.53** | -0.06 | **0.30** | 0.17 | -0.12 | 0.11 | 0.06 |
| CHAT3 | **0.20** | 0.14 | **0.21** | **0.33** | **0.55** | -0.01 | **0.42** | **0.23** | -0.15 | 0.21 | 0.13 |
| RICH3 | **0.17** | 0.14 | 0.14 | **0.34** | **0.47** | 0.00 | **0.21** | 0.12 | 0.00 | **0.23** | 0.06 |
| RICH2 | **0.16** | 0.08 | 0.14 | **0.35** | **0.44** | 0.05 | 0.20 | 0.06 | -0.07 | 0.13 | 0.07 |
| RICH1 | **0.17** | 0.15 | 0.14 | **0.36** | **0.47** | 0.01 | **0.26** | 0.13 | -0.02 | **0.23** | 0.07 |
| YAM4 | **0.17** | 0.13 | 0.16 | **0.41** | **0.49** | 0.00 | **0.36** | 0.16 | -0.03 | **0.23** | 0.09 |
| YAM3 | **0.27** | 0.20 | **0.25** | **0.56** | **0.62** | -0.07 | **0.43** | **0.28** | -0.07 | **0.35** | **0.21** |
| YAM1 | **0.24** | 0.12 | **0.25** | **0.53** | **0.59** | -0.06 | **0.37** | **0.23** | -0.03 | **0.25** | 0.10 |
| YAM7 | **0.24** | 0.14 | **0.21** | **0.56** | **0.58** | -0.02 | **0.39** | **0.20** | -0.03 | **0.24** | 0.10 |
| YAM2 | **0.22** | 0.19 | 0.16 | **0.50** | **0.58** | -0.02 | **0.39** | **0.21** | -0.01 | **0.30** | 0.16 |
| YAM9 | **0.22** | 0.10 | **0.21** | **0.49** | **0.54** | -0.01 | **0.36** | 0.20 | -0.04 | **0.26** | 0.10 |
| YAM10 | **0.14** | 0.09 | 0.04 | **0.30** | **0.44** | 0.04 | **0.35** | 0.13 | 0.03 | **0.27** | 0.15 |
| YAM6 | **0.19** | 0.09 | 0.13 | **0.42** | **0.49** | 0.03 | **0.31** | 0.14 | -0.06 | **0.24** | 0.16 |

S2.2.2 Sen’s-Slope values

**Table S2.4.** Sen’s-Slope (SS) values of historical (mm month^-1^) and seasonal (mm season^-1^) PET anomalies, as well as the beginning (Last (Freeze)/Start), end (First (Freeze)/End) and number of days (Days) of the Non-Freezing & Growing seasons, between 1980-2023 for the sub-watersheds of Montérégie. Positive trends are shown in blue-gradient colors, and negative trends are shown in red-gradient colors. Significant trends (p-value <= 0.05) are shown in bold.

| Watershed | PET | | | | | Non-Freezing Season | | | Growing Season | | |
| --- | --- | --- | --- | --- | --- | --- | --- | --- | --- | --- | --- |
|  | Overall | Winter | Spring | Summer | Autumn | Last | First | Days | Start | End | Days |
| BAIE2 | **0.005** | 0.067 | 0.088 | **0.234** | **0.390** | 0.05 | **0.31** | 0.21 | 0.07 | 0.25 | 0.00 |
| BAIE3 | **0.004** | 0.051 | -0.002 | 0.122 | **0.353** | 0.11 | **0.32** | 0.21 | 0.07 | 0.23 | 0.04 |
| BAIE1 | **0.005** | 0.069 | 0.058 | 0.172 | **0.394** | 0.12 | **0.30** | 0.16 | 0.05 | 0.26 | 0.01 |
| BAIE4 | **0.005** | 0.075 | 0.081 | 0.174 | **0.394** | 0.08 | **0.30** | 0.16 | 0 | 0.22 | 0.07 |
| CHAT5 | **0.006** | 0.054 | 0.184 | **0.354** | **0.411** | 0 | **0.29** | 0.28 | -0.09 | 0.19 | 0.17 |
| CHAT4 | **0.005** | 0.032 | 0.133 | **0.317** | **0.363** | 0.17 | **0.26** | 0.06 | -0.09 | 0.09 | 0.13 |
| CHAT2 | **0.005** | 0.055 | 0.142 | **0.294** | **0.370** | 0.14 | **0.24** | 0.06 | -0.09 | 0.12 | 0.13 |
| CHAT1 | **0.008** | 0.051 | 0.302 | **0.559** | **0.476** | -0.09 | **0.32** | 0.36 | -0.15 | 0.11 | 0.12 |
| CHAT3 | **0.008** | 0.060 | **0.332** | **0.574** | **0.490** | 0 | **0.50** | **0.45** | -0.18 | 0.25 | 0.22 |
| RICH3 | **0.007** | 0.058 | 0.218 | **0.441** | **0.443** | 0 | **0.20** | 0.25 | 0 | **0.28** | 0.13 |
| RICH2 | **0.006** | 0.032 | 0.192 | **0.452** | **0.393** | 0.07 | 0.19 | 0.11 | -0.09 | 0.13 | 0.13 |
| RICH1 | **0.007** | 0.055 | 0.213 | **0.445** | **0.435** | 0 | **0.28** | 0.26 | 0 | **0.28** | 0.14 |
| YAM4 | **0.007** | 0.060 | 0.216 | **0.451** | **0.475** | 0 | **0.44** | 0.38 | 0 | **0.33** | 0.20 |
| YAM3 | **0.012** | 0.083 | **0.360** | **0.843** | **0.608** | -0.14 | **0.50** | **0.61** | -0.10 | **0.47** | **0.47** |
| YAM1 | **0.010** | 0.054 | **0.353** | **0.749** | **0.552** | -0.11 | **0.44** | **0.50** | -0.05 | **0.33** | 0.22 |
| YAM7 | **0.010** | 0.059 | **0.323** | **0.744** | **0.560** | -0.03 | **0.44** | 0.47 | 0 | **0.33** | 0.21 |
| YAM2 | **0.009** | 0.084 | 0.225 | **0.639** | **0.537** | 0 | **0.45** | **0.50** | 0 | **0.39** | 0.32 |
| YAM9 | **0.009** | 0.044 | **0.310** | **0.652** | **0.518** | 0 | **0.42** | 0.44 | 0 | **0.33** | 0.23 |
| YAM10 | **0.006** | 0.034 | 0.070 | **0.374** | **0.429** | 0.05 | **0.39** | 0.32 | 0.03 | **0.33** | 0.23 |
| YAM6 | **0.007** | 0.030 | 0.196 | **0.524** | **0.493** | 0.04 | **0.33** | 0.30 | -0.07 | **0.30** | 0.27 |

**S2.3. Precipitation**

S2.3.1 Mann-Kendall Tau (τ) values

**Table S2.5.** Mann-Kendall tau (MK(τ)) values of historical and seasonal Precipitation & Intensity anomalies, yearly percentiles of high (P98) and low (P70) precipitation amounts, and the day of the year when the highest precipitation event occurs (Pday) between 1980-2023 for the sub-watersheds of Montérégie. Positive trends are shown in blue-gradient colors, and negative trends are shown in red-gradient colors. Significant trends (p-value <= 0.05) are shown in bold.

| Watershed | Prcp | | | | | | | | Intns | | | | |
| --- | --- | --- | --- | --- | --- | --- | --- | --- | --- | --- | --- | --- | --- |
|  | Overall | Winter | Spring | Summer | Autumn | P70 | P98 | Pday | Overall | Winter | Spring | Summer | Autumn |
| BAIE2 | **0.07** | **0.21** | 0.15 | 0.15 | 0.03 | 0.09 | **0.30** | -0.02 | **0.15** | 0.16 | **0.37** | **0.23** | 0.19 |
| BAIE3 | **0.07** | 0.13 | 0.14 | 0.18 | 0.03 | 0.02 | **0.30** | -0.03 | **0.12** | 0.01 | **0.30** | **0.21** | 0.15 |
| BAIE1 | **0.10** | **0.29** | 0.19 | 0.20 | 0.05 | 0.13 | **0.37** | -0.13 | **0.16** | 0.18 | **0.36** | **0.27** | 0.15 |
| BAIE4 | **0.09** | **0.23** | 0.15 | 0.17 | 0.07 | 0.08 | **0.36** | -0.05 | **0.14** | 0.17 | **0.36** | **0.27** | 0.14 |
| CHAT5 | 0.02 | 0.18 | 0.04 | 0.10 | -0.11 | **-0.30** | **0.24** | -0.16 | **0.16** | **0.31** | **0.36** | **0.21** | **0.23** |
| CHAT4 | **0.08** | **0.32** | 0.11 | 0.17 | -0.03 | 0.08 | **0.26** | **-0.28** | **0.12** | **0.24** | **0.26** | **0.22** | 0.08 |
| CHAT2 | 0.01 | 0.17 | 0.05 | 0.09 | -0.12 | **-0.28** | **0.25** | **-0.17** | **0.16** | **0.33** | **0.36** | 0.18 | **0.21** |
| CHAT1 | -0.04 | -0.02 | -0.07 | -0.03 | -0.17 | **-0.40** | **0.26** | **-0.25** | **0.15** | **0.21** | **0.33** | 0.19 | 0.18 |
| CHAT3 | 0.02 | 0.11 | 0.03 | 0.07 | -0.08 | **-0.22** | **0.30** | **-0.22** | **0.13** | **0.21** | **0.24** | **0.21** | 0.13 |
| RICH3 | **0.07** | 0.15 | 0.10 | 0.15 | -0.03 | 0.01 | **0.30** | 0.08 | **0.12** | **0.21** | **0.26** | **0.25** | 0.14 |
| RICH2 | 0.00 | 0.12 | 0.03 | 0.03 | -0.10 | **-0.30** | **0.36** | -0.10 | **0.12** | **0.32** | **0.36** | 0.19 | 0.10 |
| RICH1 | **0.06** | 0.12 | 0.09 | 0.14 | -0.05 | -0.09 | **0.26** | 0.12 | **0.14** | 0.20 | **0.29** | **0.29** | 0.17 |
| YAM4 | **0.07** | 0.20 | 0.16 | 0.10 | 0.05 | 0.02 | 0.20 | -0.04 | **0.11** | 0.10 | **0.29** | 0.19 | 0.19 |
| YAM3 | -0.02 | -0.10 | 0.05 | -0.13 | -0.06 | **-0.27** | 0.06 | 0.04 | 0.05 | -0.09 | **0.22** | 0.04 | 0.13 |
| YAM1 | **-0.07** | -0.08 | -0.03 | -0.13 | -0.17 | **-0.34** | 0.09 | -0.01 | **0.09** | 0.10 | **0.31** | 0.08 | 0.14 |
| YAM7 | -0.01 | -0.06 | 0.02 | -0.08 | -0.04 | **-0.24** | 0.07 | 0.09 | **0.09** | 0.08 | **0.28** | 0.05 | 0.16 |
| YAM2 | 0.05 | 0.09 | 0.14 | 0.07 | 0.02 | -0.09 | **0.22** | 0.12 | **0.14** | 0.11 | **0.34** | **0.25** | **0.25** |
| YAM9 | 0.01 | 0.07 | 0.07 | 0.04 | -0.07 | **-0.32** | 0.18 | 0.18 | **0.13** | 0.14 | **0.29** | **0.22** | 0.20 |
| YAM10 | -0.05 | 0.09 | -0.09 | -0.11 | -0.10 | **-0.31** | -0.07 | 0.04 | **0.08** | 0.19 | 0.17 | 0.07 | 0.15 |
| YAM6 | 0.06 | **0.31** | 0.09 | 0.13 | -0.05 | -0.09 | **0.26** | -0.01 | **0.14** | **0.37** | **0.27** | **0.32** | **0.24** |

S2.3.2 Sen’s-Slope values

**Table S2.6.** Sen’s-Slope (SS) values of historical (mm month^-1^) and seasonal (mm season^-1^) Precipitation & Intensity anomalies, yearly percentiles of high (P98) and low (P70) precipitation amounts, and the day of the year when the highest precipitation event occurs (Pday) between 1980-2023 for the sub-watersheds of Montérégie. Positive trends are shown in blue-gradient colors, and negative trends are shown in red-gradient colors. Significant trends (p-value <= 0.05) are shown in bold.

| Watershed | Prcp | | | | | | | | Intns | | | | |
| --- | --- | --- | --- | --- | --- | --- | --- | --- | --- | --- | --- | --- | --- |
|  | Overall | Winter | Spring | Summer | Autumn | P70 | P98 | Pday | Overall | Winter | Spring | Summer | Autumn |
| BAIE2 | **0.025** | **1.25** | 1.30 | 1.12 | 0.28 | 0.005 | **0.108** | -0.154 | **0.003** | 0.019 | **0.049** | **0.038** | 0.034 |
| BAIE3 | **0.024** | 0.95 | 1.16 | 1.27 | 0.27 | 0.001 | **0.115** | -0.278 | **0.002** | 0.002 | **0.038** | **0.036** | 0.025 |
| BAIE1 | **0.034** | **1.72** | 1.62 | 1.27 | 0.38 | 0.008 | **0.136** | -1.030 | **0.003** | 0.019 | **0.045** | **0.042** | 0.028 |
| BAIE4 | **0.031** | **1.29** | 1.23 | 1.22 | 0.52 | 0.005 | **0.122** | -0.500 | **0.003** | 0.023 | **0.040** | **0.039** | 0.026 |
| CHAT5 | 0.006 | 0.80 | 0.40 | 0.58 | -0.70 | **-0.015** | **0.093** | -1.625 | **0.003** | **0.043** | **0.050** | **0.029** | **0.039** |
| CHAT4 | **0.025** | **1.42** | 0.82 | 1.42 | -0.19 | 0.004 | **0.109** | **-3.098** | **0.002** | **0.028** | **0.034** | **0.034** | 0.011 |
| CHAT2 | 0.003 | 0.72 | 0.38 | 0.70 | -0.74 | **-0.015** | **0.092** | **-1.625** | **0.003** | **0.046** | **0.052** | 0.030 | **0.034** |
| CHAT1 | -0.013 | -0.08 | -0.59 | -0.19 | -1.33 | **-0.026** | **0.079** | **-2.278** | **0.003** | **0.034** | **0.052** | 0.027 | 0.029 |
| CHAT3 | 0.007 | 0.48 | 0.30 | 0.47 | -0.54 | **-0.010** | **0.116** | **-2.222** | **0.002** | **0.029** | **0.037** | **0.030** | 0.022 |
| RICH3 | **0.022** | 1.08 | 0.96 | 1.27 | -0.31 | 0 | **0.106** | 0.700 | **0.003** | **0.019** | **0.037** | **0.038** | 0.027 |
| RICH2 | 0.001 | 0.50 | 0.25 | 0.37 | -0.84 | **-0.013** | **0.113** | -0.938 | **0.002** | **0.036** | **0.045** | 0.029 | 0.016 |
| RICH1 | **0.020** | 0.76 | 0.86 | 1.36 | -0.35 | -0.003 | **0.109** | 1.033 | **0.003** | 0.018 | **0.037** | **0.045** | 0.030 |
| YAM4 | **0.025** | 1.13 | 1.34 | 0.71 | 0.48 | 0.001 | 0.090 | -0.360 | **0.002** | 0.011 | **0.040** | 0.029 | 0.029 |
| YAM3 | -0.008 | -0.45 | 0.44 | -0.93 | -0.63 | **-0.020** | 0.035 | 0.350 | 0.001 | -0.010 | **0.028** | 0.003 | 0.028 |
| YAM1 | **-0.023** | -0.55 | -0.19 | -1.00 | -1.40 | **-0.033** | 0.031 | -0.065 | **0.002** | 0.014 | **0.035** | 0.007 | 0.022 |
| YAM7 | -0.003 | -0.36 | 0.09 | -0.60 | -0.41 | **-0.016** | 0.031 | 0.714 | **0.002** | 0.010 | **0.033** | 0.006 | 0.030 |
| YAM2 | 0.015 | 0.44 | 0.93 | 0.60 | 0.12 | -0.007 | **0.091** | 1.143 | **0.003** | 0.014 | **0.041** | **0.039** | **0.045** |
| YAM9 | 0.004 | 0.45 | 0.61 | 0.42 | -0.62 | **-0.017** | 0.061 | 1.318 | **0.002** | 0.015 | **0.034** | **0.028** | 0.033 |
| YAM10 | -0.017 | 0.53 | -0.62 | -1.07 | -0.84 | **-0.020** | -0.029 | 0.350 | **0.002** | 0.026 | 0.015 | 0.015 | 0.024 |
| YAM6 | 0.019 | **1.76** | 0.63 | 0.99 | -0.38 | -0.005 | **0.098** | -0.097 | **0.003** | **0.038** | **0.029** | **0.050** | **0.036** |

**S2.4. Streamflow**

S2.4.1 Mann-Kendall Tau (τ) values

**Table S2.7.** Mann-Kendall tau (MK(τ)) values of historical and seasonal Streamflow anomalies, percentiles of high (Q95) and low (Q5) streamflow during Winter/Spring and Summer/Autumn seasons, and the days of the year when the highest and lowest discharge occurs during these two seasons, between 1980-2023 for the sub-watersheds of Montérégie. Positive trends are shown in blue-gradient colors, and negative trends are shown in red-gradient colors. Significant trends (p-value <= 0.05) are shown in bold.

| Watershed | Streamflow | | | | | Streamflow Q95 | | Streamflow Q5 | | Highest flow day | | Lowest flow day | |
| --- | --- | --- | --- | --- | --- | --- | --- | --- | --- | --- | --- | --- | --- |
|  | Overall | Winter | Spring | Summer | Autumn | Win/Spr | Sum/Aut | Win/Spr | Sum/Aut | Win/Spr | Sum/Aut | Win/Spr | Sum/Aut |
| BAIE2 | -0.02 | 0.16 | -0.07 | -0.18 | -0.14 | -0.13 | -0.27 | 0.03 | -0.25 | -0.10 | 0.07 | 0.16 | -0.29 |
| BAIE3 | 0.01 | 0.15 | -0.02 | -0.13 | -0.13 | 0.11 | -0.19 | 0.05 | -0.14 | -0.16 | 0.06 | 0.17 | -0.25 |
| BAIE1 | 0.01 | 0.25 | 0.08 | -0.22 | 0.10 | 0.17 | 0.05 | 0.03 | -0.21 | -0.04 | 0.06 | 0.13 | -0.23 |
| BAIE4 | 0.05 | 0.22 | -0.01 | -0.06 | 0.04 | 0.06 | -0.05 | 0.26 | 0.06 | 0.07 | 0.10 | 0.00 | -0.18 |
| CHAT5 | -0.08 | -0.04 | 0.04 | -0.09 | -0.14 | 0.00 | -0.12 | -0.09 | -0.23 | -0.07 | 0.05 | 0.03 | -0.14 |
| CHAT4 | -0.02 | -0.01 | 0.03 | -0.07 | 0.02 | -0.19 | -0.08 | -0.04 | -0.28 | 0.12 | 0.11 | -0.11 | -0.08 |
| CHAT2 | **0.08** | 0.17 | 0.15 | 0.11 | -0.03 | 0.17 | -0.01 | **0.23** | 0.05 | 0.10 | -0.16 | 0.11 | 0.17 |
| CHAT1 | **0.07** | 0.14 | 0.12 | 0.16 | -0.07 | 0.07 | 0.03 | **0.22** | 0.00 | 0.04 | -0.12 | 0.05 | **0.39** |
| CHAT3 | -0.05 | -0.10 | 0.06 | -0.20 | -0.24 | -0.12 | -0.22 | -0.14 | -0.24 | 0.25 | 0.23 | -0.02 | 0.11 |
| RICH3 | 0.04 | 0.20 | 0.22 | -0.32 | 0.13 | 0.20 | 0.12 | -0.05 | 0.00 | -0.14 | 0.35 | 0.20 | -0.16 |
| RICH2 | **0.12** | **0.25** | 0.14 | **0.25** | 0.11 | **0.21** | 0.13 | **0.23** | 0.01 | 0.04 | -0.16 | 0.05 | **0.27** |
| RICH1 | **0.10** | **0.21** | 0.11 | 0.18 | 0.00 | 0.12 | 0.12 | 0.19 | **0.24** | 0.06 | -0.04 | 0.03 | **0.37** |
| YAM4 | **0.11** | **0.28** | 0.10 | 0.18 | 0.04 | 0.16 | 0.09 | **0.37** | 0.17 | 0.00 | 0.04 | **0.25** | 0.19 |
| YAM3 | **0.06** | 0.06 | 0.17 | 0.12 | 0.07 | 0.13 | 0.18 | 0.02 | 0.01 | 0.02 | -0.08 | 0.05 | 0.06 |
| YAM1 | **0.06** | 0.16 | 0.05 | 0.08 | 0.01 | -0.11 | 0.06 | 0.06 | 0.13 | -0.03 | -0.10 | 0.11 | 0.07 |
| YAM7 | 0.06 | 0.16 | 0.07 | 0.07 | 0.03 | 0.05 | 0.07 | -0.03 | 0.03 | -0.09 | -0.14 | 0.09 | 0.15 |
| YAM2 | **0.08** | 0.16 | 0.17 | 0.12 | 0.04 | 0.17 | 0.03 | 0.16 | 0.00 | -0.04 | -0.02 | 0.16 | 0.09 |
| YAM9 | 0.06 | 0.16 | 0.03 | -0.01 | 0.14 | 0.08 | 0.00 | 0.03 | 0.10 | 0.05 | -0.08 | 0.05 | 0.07 |
| YAM10 | 0.06 | 0.21 | 0.03 | -0.16 | 0.02 | 0.04 | -0.20 | 0.09 | 0.04 | 0.11 | 0.16 | 0.14 | -0.10 |
| YAM6 | **0.11** | 0.19 | **0.21** | **0.25** | 0.10 | 0.15 | **0.21** | 0.16 | -0.01 | 0.10 | -0.01 | 0.01 | -0.01 |

S2.4.2 Sen’s-Slope values

**Table S2.8.** Sen’s-Slope (SS) values of historical (mm month^-1^) and seasonal (mm season^-1^) Streamflow anomalies, percentiles of high (Q95) and low (Q5) streamflow during Winter/Spring and Summer/Autumn seasons, and the days of the year when the highest and lowest discharge occurs during these two seasons, between 1980-2023 for the sub-watersheds of Montérégie. Positive trends are shown in blue-gradient colors, and negative trends are shown in red-gradient colors. Significant trends (p-value <= 0.05) are shown in bold.

| Watershed | Streamflow | | | | | Streamflow Q95 | | Streamflow Q5 | | Highest flow day | | Lowest flow day | |
| --- | --- | --- | --- | --- | --- | --- | --- | --- | --- | --- | --- | --- | --- |
|  | Overall | Winter | Spring | Summer | Autumn | Win/Spr | Sum/Aut | Win/Spr | Sum/Aut | Win/Spr | Sum/Aut | Win/Spr | Sum/Aut |
| BAIE2 | -0.01 | 2.16 | -0.99 | -2.21 | -2.26 | -0.050 | -0.111 | 0.0010 | -0.0027 | -0.53 | 0.67 | 1.10 | -1.75 |
| BAIE3 | 0.01 | 1.76 | -0.27 | -1.25 | -1.64 | 0.055 | -0.086 | 0.0006 | -0.0007 | -1.90 | 0.57 | 0.46 | -1.22 |
| BAIE1 | 0.00 | 2.51 | 0.84 | -1.51 | 1.31 | 0.074 | 0.008 | 0.0005 | -0.0012 | -0.15 | 0.40 | 1.13 | -0.75 |
| BAIE4 | 0.02 | 2.54 | -0.17 | -0.61 | 1.12 | 0.039 | -0.030 | 0.0052 | 0.0006 | 0.49 | 0.75 | 0 | -1.09 |
| CHAT5 | -0.04 | -0.54 | 1.05 | -1.27 | -1.25 | -0.004 | -0.032 | -0.0018 | -0.0019 | -0.55 | 0.56 | 0 | -1.07 |
| CHAT4 | -0.01 | -0.45 | 0.79 | -0.63 | 0.04 | -0.116 | -0.011 | -0.0023 | -0.0022 | 0.86 | 1.11 | -1.46 | -0.50 |
| CHAT2 | **0.01** | 0.99 | 1.12 | 0.34 | -0.12 | 0.030 | -0.002 | **0.0021** | 0.0003 | 0.31 | -0.57 | 0.29 | **0.60** |
| CHAT1 | **0.01** | 0.79 | 0.79 | 0.56 | -0.28 | 0.012 | 0.005 | **0.0030** | 0.0000 | 0.13 | -0.43 | 0.14 | 1.15 |
| CHAT3 | -0.02 | -1.75 | 0.92 | -2.01 | -2.53 | -0.044 | -0.079 | -0.0053 | -0.0023 | 4.00 | 1.57 | 0 | 0.67 |
| RICH3 | 0.03 | 2.74 | 5.67 | -4.75 | 2.13 | 0.185 | 0.030 | -0.0025 | 0.0000 | -1.56 | 3.48 | 3.83 | -1.00 |
| RICH2 | **0.02** | **1.18** | 1.13 | **0.88** | 0.71 | **0.057** | 0.026 | **0.0016** | 0.0000 | 0.12 | -0.65 | 0.17 | **0.89** |
| RICH1 | **0.02** | **1.41** | 0.88 | 0.66 | 0.03 | 0.039 | 0.025 | 0.0017 | **0.0006** | 0.17 | -0.15 | 0.10 | **1.19** |
| YAM4 | **0.03** | **1.98** | 1.00 | 0.93 | 0.37 | 0.046 | 0.019 | **0.0074** | 0.0019 | 0 | 0.23 | **1.06** | 0.88 |
| YAM3 | **0.01** | 0.49 | 1.51 | 0.42 | 0.33 | 0.026 | 0.035 | 0.0005 | 0.0001 | 0.06 | -0.27 | 0.17 | 0.43 |
| YAM1 | **0.02** | 0.89 | 0.57 | 0.36 | 0.03 | -0.026 | 0.014 | 0.0012 | 0.0012 | -0.06 | -0.37 | 0.21 | 0.29 |
| YAM7 | 0.01 | 1.20 | 0.64 | 0.39 | 0.14 | 0.015 | 0.016 | -0.0005 | 0.0004 | -0.58 | -0.75 | 0.23 | 0.92 |
| YAM2 | **0.02** | 0.80 | 1.31 | 0.46 | 0.20 | 0.037 | 0.006 | 0.0020 | -0.0001 | -0.09 | -0.08 | 0.42 | 0.33 |
| YAM9 | 0.02 | 1.15 | 0.40 | -0.03 | 1.28 | 0.028 | 0.000 | 0.0004 | 0.0007 | 0.29 | -0.60 | 0.04 | 0.37 |
| YAM10 | 0.03 | 2.51 | 0.77 | -1.57 | 0.23 | 0.019 | -0.069 | 0.0020 | 0.0001 | 0.42 | 1.53 | 0.50 | -0.50 |
| YAM6 | **0.02** | 0.86 | **1.80** | **0.84** | 0.58 | 0.035 | **0.044** | 0.0017 | 0.0000 | 0.13 | -0.06 | 0 | 0 |

**S2.5. Simulated Ps and Percolation**

S2.5.1. Mann-Kendall Tau (τ) values

**Table S2.9.** Mann-Kendall tau (MK(τ)) values of yearly simulated effective precipitation (Ps) and percolation for three different periods (the overall monthly time-series, winter-spring, and summer-autumn), as well as for the days on which their peaks occur during the year, between 1980-2023 for the sub-watersheds of Montérégie. Positive trends are shown in blue-gradient colors, and negative trends are shown in red-gradient colors. Significant trends (p-value <= 0.05) are shown in bold.

| Watershed | Percolation | | | Percolation peak | | Ps | | | Ps peak | |
| --- | --- | --- | --- | --- | --- | --- | --- | --- | --- | --- |
|  | Overall | Win/Spr | Sum/Aut | Win/Spr | Sum/Aut | Overall | Win/Spr | Sum/Aut | Win/Spr | Sum/Aut |
| BAIE2 | **0.15** | 0.30 | 0.08 | 0.26 | 0.14 | 0.06 | 0.19 | 0.16 | -0.21 | 0.00 |
| BAIE3 | **0.17** | 0.29 | 0.11 | 0.26 | 0.16 | 0.05 | **0.31** | 0.03 | -0.21 | 0.09 |
| BAIE1 | **0.19** | **0.42** | 0.22 | -0.02 | -0.09 | 0.07 | 0.12 | 0.28 | -0.12 | 0.05 |
| BAIE4 | **0.18** | **0.39** | 0.09 | 0.04 | 0.13 | 0.05 | 0.16 | 0.15 | -0.28 | -0.01 |
| CHAT5 | **-0.16** | -0.21 | -0.31 | 0.06 | -0.06 | -0.02 | -0.04 | -0.05 | -0.20 | 0.20 |
| CHAT4 | 0.02 | 0.13 | -0.08 | -0.01 | -0.13 | 0.03 | 0.04 | 0.16 | **-0.37** | **-0.44** |
| CHAT2 | -0.01 | 0.07 | -0.10 | 0.05 | -0.08 | 0.05 | 0.20 | 0.06 | 0.01 | 0.19 |
| CHAT1 | **-0.11** | -0.11 | **-0.24** | -0.10 | -0.05 | 0.02 | 0.12 | -0.05 | 0.05 | 0.10 |
| CHAT3 | **-0.09** | -0.03 | **-0.37** | -0.07 | 0.24 | 0.03 | 0.15 | 0.15 | -0.17 | -0.13 |
| RICH3 | -0.01 | 0.12 | -0.35 | 0.13 | 0.27 | 0.00 | **-0.47** | 0.06 | -0.29 | 0.14 |
| RICH2 | -0.03 | 0.02 | -0.17 | 0.01 | -0.11 | 0.03 | 0.15 | 0.02 | 0.01 | **0.22** |
| RICH1 | 0.04 | 0.16 | -0.09 | 0.11 | -0.05 | **0.07** | 0.17 | **0.22** | -0.09 | 0.06 |
| YAM4 | **0.06** | 0.16 | 0.01 | 0.14 | 0.06 | **0.07** | **0.29** | 0.18 | -0.09 | 0.14 |
| YAM3 | **-0.11** | -0.10 | **-0.26** | 0.19 | -0.04 | 0.02 | 0.08 | 0.02 | 0.02 | **0.28** |
| YAM1 | **-0.15** | -0.15 | **-0.32** | 0.10 | -0.04 | 0.02 | 0.14 | -0.02 | 0.01 | 0.20 |
| YAM7 | **-0.07** | -0.05 | -0.16 | 0.13 | 0.13 | 0.02 | 0.02 | 0.13 | -0.07 | **0.25** |
| YAM2 | 0.03 | 0.11 | -0.05 | 0.13 | 0.03 | **0.06** | 0.21 | **0.22** | -0.06 | **0.21** |
| YAM9 | -0.06 | -0.04 | -0.20 | 0.10 | -0.07 | 0.02 | 0.01 | 0.14 | -0.12 | 0.14 |
| YAM10 | **-0.09** | -0.05 | -0.30 | -0.01 | -0.20 | -0.07 | -0.14 | -0.26 | **-0.31** | 0.03 |
| YAM6 | 0.02 | 0.15 | -0.09 | -0.01 | -0.08 | 0.04 | 0.17 | 0.19 | -0.11 | 0.00 |

S2.5.2. Sen’s-Slope values

**Table S2.10.** Sen’s-Slope (SS) values of yearly simulated effective precipitation (Ps; mm year^-1^)) and percolation (mm year^-1^) for three different periods (the overall monthly time-series, winter-spring, and summer-autumn), as well as for the days on which their peaks occur during the year, between 1980-2023 for the sub-watersheds of Montérégie. Positive trends are shown in blue-gradient colors, and negative trends are shown in red-gradient colors. Significant trends (p-value <= 0.05) are shown in bold.

| Watershed | Percolation | | | Percolation peak | | Ps | | | Ps peak | |
| --- | --- | --- | --- | --- | --- | --- | --- | --- | --- | --- |
|  | Overall | Win/Spr | Sum/Aut | Win/Spr | Sum/Aut | Overall | Win/Spr | Sum/Aut | Win/Spr | Sum/Aut |
| BAIE2 | **0.01** | 1.47 | 0.36 | 0.89 | 0.17 | 0.02 | 1.03 | 0.93 | -2.06 | 0.00 |
| BAIE3 | **0.02** | 1.56 | 0.38 | 0.74 | 0.17 | 0.02 | **1.92** | 0.27 | -2.54 | 0.33 |
| BAIE1 | **0.01** | **1.70** | 0.70 | 0.00 | -0.06 | 0.02 | 0.56 | 1.81 | -1.00 | 0.17 |
| BAIE4 | **0.01** | **1.39** | 0.30 | 0.18 | 0.40 | 0.01 | 0.88 | 1.11 | -2.38 | -0.06 |
| CHAT5 | **-0.01** | -1.09 | -0.87 | 0.22 | -0.33 | -0.01 | -0.27 | -0.25 | -4.11 | 2.50 |
| CHAT4 | 0.00 | 0.34 | -0.09 | 0.00 | -0.30 | 0.01 | 0.05 | 2.13 | **-7.26** | **-5.50** |
| CHAT2 | 0.00 | 0.10 | -0.12 | 0.076923 | -0.13 | 0.01 | 0.50 | 0.18 | 0.02 | 0.90 |
| CHAT1 | **0.00** | -0.28 | **-0.34** | -0.17 | -0.08 | 0.00 | 0.32 | -0.20 | 0.26 | 0.51 |
| CHAT3 | **-0.01** | -0.22 | **-1.28** | -0.17 | 1.00 | 0.01 | 1.52 | 1.44 | -1.63 | -2.00 |
| RICH3 | 0.00 | 0.27 | -0.26 | 0.84 | 1.95 | 0.00 | **-2.16** | 0.54 | -1.20 | 1.35 |
| RICH2 | 0.00 | 0.03 | -0.19 | 0 | -0.07 | 0.00 | 0.34 | 0.06 | 0.00 | **0.95** |
| RICH1 | 0.00 | 0.29 | -0.11 | 0.188345 | -0.04 | **0.01** | 0.45 | **0.58** | -0.25 | 0.22 |
| YAM4 | **0.00** | 0.31 | 0.01 | 0.24 | 0.05 | **0.01** | **0.59** | 0.55 | -0.48 | 0.48 |
| YAM3 | **-0.01** | -0.30 | **-0.55** | 0.41 | -0.01 | 0.00 | 0.37 | 0.05 | 0.08 | **1.24** |
| YAM1 | **-0.01** | -0.38 | **-0.59** | 0.17 | -0.04 | 0.00 | 0.33 | -0.09 | 0.04 | 0.81 |
| YAM7 | **0.00** | -0.16 | -0.35 | 0.21 | 0.11 | 0.00 | 0.07 | 0.73 | -0.43 | **1.33** |
| YAM2 | 0.00 | 0.18 | -0.09 | 0.20 | 0.00 | **0.01** | 0.44 | **0.87** | -0.33 | **0.85** |
| YAM9 | 0.00 | -0.16 | -0.42 | 0.21 | -0.04 | 0.00 | 0.05 | 0.70 | -1.44 | 0.96 |
| YAM10 | **0.00** | -0.09 | -0.66 | 0.00 | -1.17 | -0.02 | -0.58 | -2.19 | **-3.32** | 0.21 |
| YAM6 | 0.00 | 0.23 | -0.12 | 0 | -0.12 | 0.01 | 0.35 | 0.65 | -0.45 | 0.00 |

**S2.6. Piezometers**

S2.6.1. Mann-Kendall Tau (τ) values

**Table S2.11.** Mann-Kendall p-values (MK(pval)) of historical and seasonal GW levels anomalies, and the days on which GW levels peaks occur during two periods of the year (winter-spring and summer-autumn), between 2000-2023 for the piezometers of Montérégie. Positive trends are shown in blue-gradient colors, and negative trends are shown in red-gradient colors. Significant trends (p-value <= 0.05) are shown in bold.

| Piezometer | Groundwater levels | | | | | Recharge peak | |
| --- | --- | --- | --- | --- | --- | --- | --- |
|  | Historical | Winter | Spring | Summer | Autumn | Win/Spr | Sum/Aut |
| BAIE4 | **-0.22** | -0.21 | -0.03 | -0.12 | -0.16 | -0.01 | 0.30 |
| BAIE3 | -0.06 | 0.00 | 0.12 | 0.15 | 0.21 | -0.14 | -0.18 |
| BAIE6 | 0.01 | 0.05 | 0.27 | -0.20 | -0.02 | -0.12 | -0.07 |
| BAIE7 | -0.08 | -0.11 | -0.07 | -0.27 | -0.05 | -0.16 | 0.00 |
| BAIE2 | -0.05 | 0.00 | 0.06 | -0.06 | -0.09 | -0.25 | 0.02 |
| BAIE1 | **-0.16** | -0.03 | 0.18 | -0.18 | -0.35 | 0.05 | 0.11 |
| BAIE5 | **-0.24** | -0.02 | 0.18 | -0.27 | -0.31 | 0.00 | 0.39 |
| CHAT13 | **-0.64** | **-0.70** | **-0.68** | **-0.82** | **-0.82** | -0.09 | -0.10 |
| CHAT15 | **-0.17** | -0.32 | 0.02 | -0.15 | -0.33 | 0.12 | -0.34 |
| CHAT9 | **-0.15** | -0.03 | 0.15 | -0.25 | -0.22 | **0.49** | 0.16 |
| CHAT4 | **-0.45** | **-0.62** | **-0.50** | **-0.50** | **-0.45** | 0.08 | 0.02 |
| CHAT5 | **-0.57** | **-0.71** | **-0.56** | **-0.61** | **-0.62** | 0.07 | -0.01 |
| CHAT21 | **-0.12** | -0.06 | 0.22 | -0.31 | -0.27 | 0.05 | 0.12 |
| CHAT10 | **-0.26** | **-0.40** | -0.30 | **-0.43** | **-0.37** | -0.06 | 0.17 |
| CHAT20 | **-0.64** | **-0.79** | **-0.63** | **-0.70** | **-0.75** | 0.16 | 0.09 |
| CHAT28 | **-0.58** | **-0.49** | **-0.51** | **-0.43** | **-0.42** | 0.21 | -0.04 |
| CHAT17 | **-0.61** | **-0.75** | **-0.61** | **-0.67** | **-0.69** | 0.01 | 0.08 |
| CHAT18 | **-0.63** | **-0.78** | **-0.60** | **-0.70** | **-0.74** | 0.02 | 0.09 |
| CHAT6 | **-0.28** | -0.27 | -0.16 | -0.29 | -0.24 | -0.20 | -0.18 |
| CHAT24 | 0.01 | -0.05 | 0.06 | -0.09 | 0.04 | -0.20 | 0.00 |
| CHAT29 | **-0.32** | 0.32 | 0.06 | -0.02 | -0.08 | -0.05 | -0.01 |
| CHAT7 | -0.01 | **-0.55** | -0.30 | -0.15 | -0.10 | 0.03 | 0.23 |
| CHAT30 | **0.17** | 0.07 | -0.18 | -0.18 | 0.08 | -0.14 | -0.09 |
| CHAT32 | **0.17** | -0.05 | 0.08 | 0.12 | 0.12 | 0.09 | -0.08 |
| CHAT25 | **0.59** | **0.36** | **0.39** | **0.36** | **0.41** | -0.12 | 0.05 |
| CHAT31 | **0.20** | 0.09 | -0.01 | 0.15 | 0.22 | -0.09 | -0.12 |
| CHAT27 | **0.59** | **0.56** | **0.56** | **0.67** | **0.69** | -0.07 | 0.15 |
| RICH3 | **-0.36** | -0.36 | -0.36 | -0.42 | **-0.53** | 0.31 | -0.27 |
| RICH9 | **-0.12** | -0.21 | 0.00 | -0.12 | 0.16 | -0.03 | 0.35 |
| RICH7 | **0.58** | **0.70** | **0.67** | **0.82** | **0.78** | 0.22 | -0.06 |
| RICH8 | **0.22** | 0.33 | **0.64** | 0.39 | 0.31 | 0.03 | 0.22 |
| RICH4 | 0.07 | 0.21 | **0.45** | 0.09 | 0.36 | -0.08 | 0.30 |
| VS3 | **-0.54** | **-0.61** | **-0.83** | 0.33 | **-0.61** | -0.24 | -0.14 |
| VS1 | 0.06 | 0.17 | 0.00 | 0.07 | 0.11 | -0.42 | 0.11 |
| YAM9 | **0.14** | 0.39 | 0.39 | 0.03 | 0.20 | -0.31 | 0.32 |
| YAM10 | **-0.22** | -0.27 | **-0.45** | -0.24 | -0.21 | -0.31 | 0.39 |
| YAM20 | **-0.34** | 0.00 | 0.22 | 0.20 | 0.07 | -0.07 | -0.07 |
| YAM16 | **-0.43** | **-0.49** | -0.24 | -0.38 | -0.31 | 0.00 | -0.37 |
| YAM15 | -0.08 | -0.13 | 0.20 | -0.13 | -0.09 | 0.05 | 0.20 |
| YAM14 | **0.14** | 0.24 | 0.27 | 0.13 | 0.24 | -0.24 | 0.29 |
| YAM13 | **0.19** | 0.27 | 0.38 | 0.24 | 0.20 | 0.15 | -0.12 |
| YAM18 | **-0.24** | -0.16 | 0.09 | -0.03 | -0.06 | 0.08 | -0.36 |
| YAM19 | **-0.11** | **-0.40** | -0.12 | **-0.40** | -0.32 | 0.06 | 0.04 |
| YAM7 | 0.06 | 0.15 | 0.15 | 0.06 | 0.24 | -0.13 | 0.31 |
| YAM4 | 0.02 | 0.18 | 0.24 | 0.15 | 0.20 | -0.38 | **0.52** |
| YAM12 | **-0.33** | -0.27 | -0.20 | **-0.53** | **-0.51** | 0.44 | 0.00 |
| YAM11 | 0.01 | 0.24 | 0.35 | -0.38 | -0.29 | -0.08 | **0.64** |
| YAM3 | **-0.28** | -0.16 | -0.09 | -0.35 | -0.09 | 0.18 | -0.26 |
| YAM2 | **-0.47** | -0.33 | -0.24 | **-0.60** | -0.47 | 0.15 | -0.18 |
| YAM6 | **0.33** | **0.48** | **0.55** | **0.45** | 0.45 | 0.38 | -0.14 |

S2.6.2. Sen’s-Slope values

**Table S2.12.** Sen’s-Slope (SS) values of historical (cm month^-1^) and seasonal (cm season^-1^)

GW levels anomalies, and the days on which GW levels peaks occur during two periods of the year (winter-spring and summer-autumn), between 2000-2023 for the piezometers of Montérégie. Positive trends are shown in blue-gradient colors, and negative trends are shown in red-gradient colors. Significant trends (p-value <= 0.05) are shown in bold.

| Piezometer | Groundwater levels | | | | | Recharge peak | |
| --- | --- | --- | --- | --- | --- | --- | --- |
|  | Historical | Winter | Spring | Summer | Autumn | Win/Spr | Sum/Aut |
| BAIE4 | **-0.1** | -1.3 | -0.2 | -1.0 | -1.5 | -0.1 | 7.0 |
| BAIE3 | -0.1 | -0.3 | 0.8 | 2.0 | 2.9 | 0.0 | -0.6 |
| BAIE6 | 0.0 | 0.1 | 0.4 | -0.7 | 0.0 | -2.3 | -0.3 |
| BAIE7 | 0.0 | -0.4 | -0.1 | -1.2 | -0.4 | -3.7 | 0.0 |
| BAIE2 | -0.1 | -0.3 | 1.9 | -3.8 | -2.2 | -3.9 | 0.0 |
| BAIE1 | **-0.1** | -0.1 | 0.4 | -0.9 | -2.0 | 0.4 | 1.4 |
| BAIE5 | **-0.1** | -0.1 | 0.4 | -1.7 | -3.1 | 0.0 | 4.7 |
| CHAT13 | **-0.6** | -8.5 | -6.8 | -8.2 | -8.5 | -0.5 | -0.1 |
| CHAT15 | **-0.1** | -2.5 | 0.2 | -1.2 | -2.1 | 0.7 | -0.7 |
| CHAT9 | **-0.1** | -0.1 | 0.6 | -2.4 | -1.6 | **4.5** | 0.7 |
| CHAT4 | **-0.4** | -4.0 | -2.7 | -7.6 | -5.3 | 0.7 | 0.1 |
| CHAT5 | **-0.7** | -8.2 | -5.8 | -12.4 | -10.0 | 0.7 | 0.0 |
| CHAT21 | **-0.1** | -0.3 | 1.1 | -1.8 | -2.2 | 0.3 | 0.4 |
| CHAT10 | **-0.1** | -2.2 | -0.6 | -2.5 | -3.1 | -0.6 | 1.0 |
| CHAT20 | **-1.4** | -18.2 | -12.5 | -18.3 | -20.2 | 1.0 | 0.2 |
| CHAT28 | **-1.5** | -23.4 | -17.6 | -20.3 | -17.2 | 2.2 | -0.2 |
| CHAT17 | **-0.9** | -11.8 | -7.7 | -15.2 | -15.2 | 0.0 | 0.2 |
| CHAT18 | **-1.2** | -15.8 | -10.2 | -17.9 | -18.5 | 0.1 | 0.2 |
| CHAT6 | **-0.3** | -3.9 | -1.5 | -2.9 | -3.2 | -0.8 | -0.4 |
| CHAT24 | 0.0 | -0.3 | 0.1 | -0.3 | 0.4 | -0.9 | 0.0 |
| CHAT29 | **-0.3** | 1.5 | 0.4 | -0.4 | -0.9 | -0.3 | 0.0 |
| CHAT7 | 0.0 | **-1.8** | -0.7 | -0.4 | -0.8 | 0.5 | 1.2 |
| CHAT30 | **0.1** | 0.8 | -0.4 | -0.6 | 1.1 | -1.7 | -0.1 |
| CHAT32 | **0.1** | -0.9 | 0.7 | 0.7 | 0.8 | 0.3 | -0.3 |
| CHAT25 | **0.5** | **1.9** | **2.9** | **2.2** | **2.4** | -1.1 | 0.2 |
| CHAT31 | **0.1** | 0.8 | -0.1 | 0.4 | 1.8 | -1.5 | -0.4 |
| CHAT27 | **0.3** | **2.0** | **2.1** | **5.0** | **5.2** | -0.3 | 0.8 |
| RICH3 | **-0.3** | -4.0 | -3.3 | -4.6 | **-5.2** | 9.4 | -1.1 |
| RICH9 | **-0.1** | -1.2 | 0.0 | -1.9 | 2.6 | -0.5 | 10.5 |
| RICH7 | **1.3** | **16.3** | **15.2** | **16.0** | **18.4** | 3.3 | -0.6 |
| RICH8 | **0.2** | 2.2 | **4.4** | 4.0 | 4.1 | 1.1 | 1.0 |
| RICH4 | 0.1 | 1.4 | **1.8** | 0.6 | 9.7 | -0.8 | 4.8 |
| VS3 | **-0.9** | **-9.4** | **-9.1** | 1.8 | **-11.1** | -3.4 | -0.4 |
| VS1 | 0.0 | 1.2 | 0.3 | 0.3 | 0.7 | -2.7 | 2.0 |
| YAM9 | **0.1** | 1.1 | 1.3 | 0.2 | 0.9 | -2.3 | 4.7 |
| YAM10 | **-0.1** | -0.6 | **-0.9** | -1.2 | -0.4 | -3.1 | 12.5 |
| YAM20 | **0.0** | 0.0 | 0.7 | 0.5 | 0.1 | -0.7 | -1.5 |
| YAM16 | **-0.4** | **-3.6** | -1.9 | -5.1 | -4.8 | 0.0 | -1.5 |
| YAM15 | -0.1 | -0.7 | 1.5 | -1.2 | -0.9 | 0.4 | 8.5 |
| YAM14 | **0.1** | 2.4 | 1.4 | 0.5 | 2.4 | -3.9 | 2.3 |
| YAM13 | **0.1** | 3.1 | 3.3 | 1.3 | 1.8 | 1.2 | 0.0 |
| YAM18 | **-0.1** | -1.8 | 1.1 | -0.6 | -0.6 | 0.9 | -0.9 |
| YAM19 | **0.0** | **-2.1** | -0.5 | **-1.9** | -2.4 | 0.5 | 0.3 |
| YAM7 | 0.1 | 2.8 | 2.4 | 1.1 | 3.9 | -1.5 | 7.4 |
| YAM4 | 0.0 | 0.6 | 1.0 | 1.6 | 3.7 | -2.6 | **18.0** |
| YAM12 | **-0.2** | -1.8 | -0.7 | **-1.4** | **-3.5** | 11.0 | 0.0 |
| YAM11 | 0.0 | 1.2 | 1.4 | -5.1 | -4.6 | -0.8 | **13.0** |
| YAM3 | **-0.2** | -2.1 | -0.9 | -1.1 | -0.5 | 1.0 | -3.4 |
| YAM2 | **-0.2** | -2.0 | -1.3 | **-2.0** | -1.7 | 0.6 | -0.6 |
| YAM6 | **0.3** | **2.8** | **5.9** | **4.1** | 7.4 | 1.0 | -1.5 |

**S3. MICE Imputation Results**

In this paper, we decided to discard the time series that yield FMI values of more than 40% after imputations, as they show the largest differences between the standard deviation values before and after imputations (Figure S3). The dates of the first record and the percentage of missing data for each meteorological, hydrometric, and piezometric stations that have been imputed, as well as their obtained pooling results are shown in supplementary section S1. Groundwater level records prior to 2000 contain the highest number of missing daily values (~86%) and the highest FMI values (~94%) (Table S6). Similarly, some piezometers with records after 2000 still show high average FMI values (~60%) (Table S7 and Table S8). All these piezometric records have been discarded from the statistical analysis (Figure S3).

**Figure S3.** Comparison of the standard deviation (SD) values of the groundwater levels of the different piezometers compiled in this work (circles), before and after the imputations of missing values. Piezometers that were discarded because the FMI values were too high are shown in red.


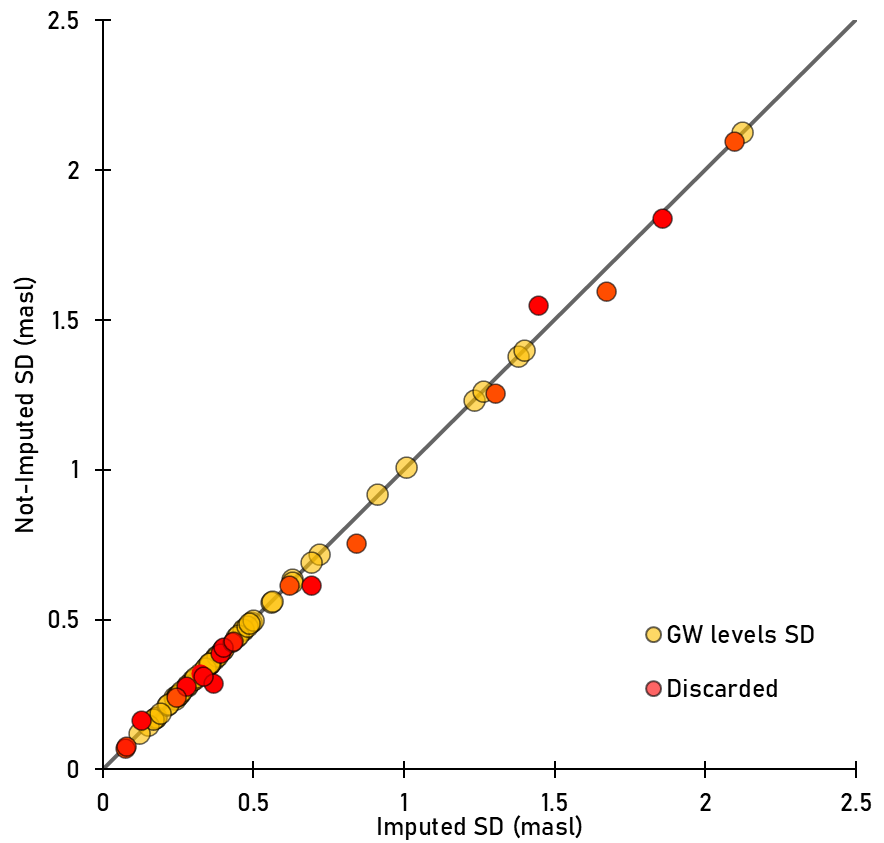


In the case of imputations from meteorological and hydrometric station records, the percentages of missing values, and the FMI values, are well below 40%. For meteorological stations (Table S1, Table S2, and Table S3), an average of 3.5% of the daily values and 6% of FMI are missing from temperature and precipitation data. FARN and GRAN stations have the highest percentage of missing values and FMI values (~11.5% and ~26%, respectively). For hydrometric stations (Table S4 and Table S5), the imputed streamflow values show an average of ~3.2% missing data and ~7% FMI values. RICH3 station has the highest ratio of missing data (~14%) and CHAT2 the highest FMI values (~20%). All imputations made at the meteorological and hydrometric stations were considered in the statistical analysis.

**Figure S4.** Representative comparison of observed and simulated hydrological variables for watershed YAM9 using the GR4J+CemaNeige model.

From top to bottom: (a) daily solid and liquid precipitation inputs, (b) air temperature time series, (c) simulated snowpack evolution, (d) daily observed (black) and simulated (orange) streamflow, (e) monthly climatology of precipitation and 30‑day rolling mean streamflow, (f) flow‑duration curves of observed and simulated discharge (log scale), and (g) scatter plot of observed versus simulated daily streamflow (log scale). This figure illustrates the overall performance of the GR4J+CemaNeige model calibration for a representative watershed and supports the interpretation of simulated effective precipitation and percolation trends. Plots were generated using the plot() function of the airGR R package.


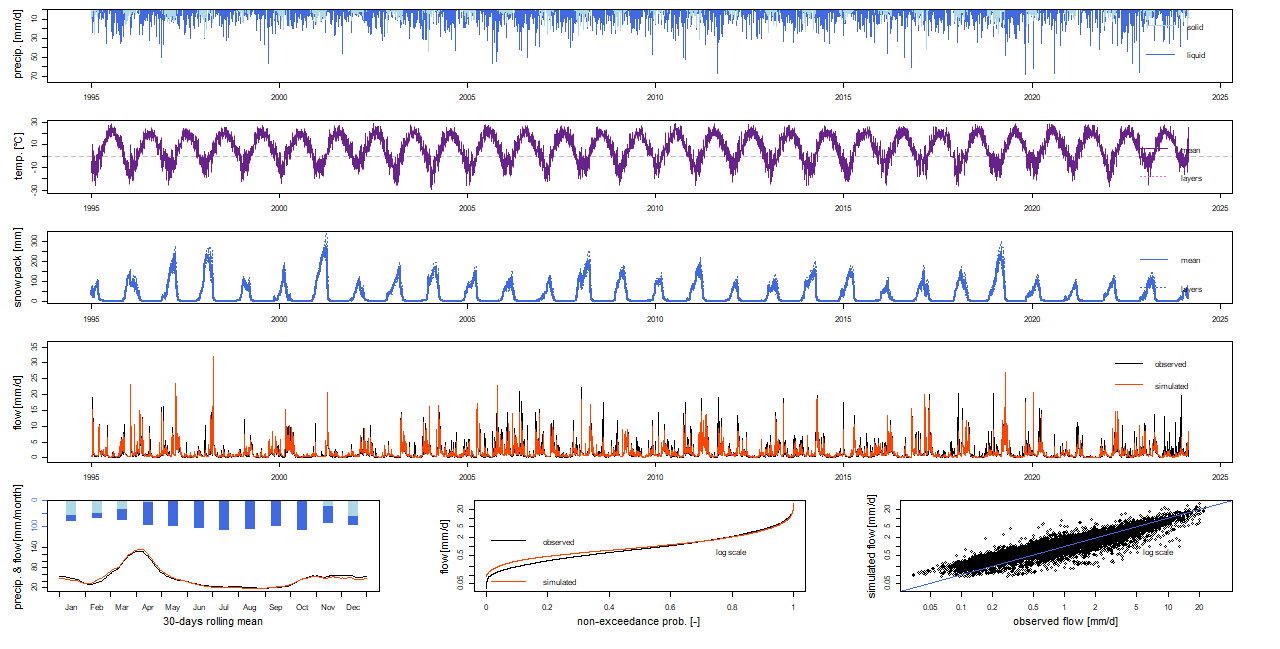


a)

b)

c)

d)

e)

f)

g)
